# Molecularly matched targeted therapies plus radiotherapy in glioblastoma: the phase 1/2a N^2^M^2^ umbrella trial

**DOI:** 10.1038/s41591-025-03928-9

**Published:** 2025-09-05

**Authors:** Wolfgang Wick, Lisa-Marie Lanz, Antje Wick, Inga Harting, Susan Dettmer, Abigail K. Suwala, Ralf Ketter, Ghazaleh Tabatabai, Corinna Seliger, Martin Glas, Michael C. Burger, Marco Timmer, Florian A. Ringel, Iris Mildenberger, Walter J. Schulz-Schaeffer, Frank Winkler, Laila König, Christel Herold-Mende, Andreas Eisenmenger, Stefan M. Pfister, Mirjam Renovanz, Martin Bendszus, Felix Sahm, Michael Platten, Tobias Kessler

**Affiliations:** 1 Neurology, University Clinic Heidelberg, Heidelberg University & German Cancer Consortium (DKTK) and CCU Neurooncology, German Cancer Research Center, Heidelberg, Germany; 2 NCT Trial Center, University Clinic Heidelberg, Heidelberg University & German Cancer Research Center, Heidelberg, Germany; 3 Neuroradiology, University Clinic Heidelberg, Heidelberg University, Heidelberg, Germany; 4 Neuropathology, University Clinic Heidelberg, Heidelberg University & German Cancer Research Center, Heidelberg, Germany; 5 Neurosurgery, Saarland University, Homburg, Germany; 6 Department of Neurology & Interdisciplinary Neuro-Oncology, University Hospital Tübingen, Hertie Institute for Clinical Brain Research, Eberhard Karls University, Tübingen, Germany; 7 Centre for Neuro-Oncology, Comprehensive Cancer Center Tübingen–Stuttgart, Tübingen, Germany; 8 Department of Neurology, University Hospital Knappschaftskrankenhaus Bochum, Ruhr University Bochum, Bochum, Germany; 9 Division of Clinical Neurooncology, Department of Neurology and Center for Translational Neuro- and Behavioral Sciences (C-TNBS), University Medicine Essen, University Duisburg–Essen, Essen, Germany; 10 Dr. Senckenberg Institute of Neurooncology, Goethe University Frankfurt and Goethe University Hospital, Frankfurt, Germany; 11 Department of Neurosurgery, University Hospital Cologne, Cologne, Germany; 12 Neurosurgery, University Medicine Mainz, Mainz, Germany; 13 German Cancer Consortium (DKTK) & CCU Neuroimmunology and Brain Tumor Immunology, German Cancer Research Center Heidelberg, Heidelberg, Germany; 14 Department of Neurology, Medical Faculty Mannheim, Mannheim Center for Translational Neuroscience (MCTN), Heidelberg University & DKFZ Hector Cancer Institute at the University Medical Center Mannheim, Mannheim, Germany; 15 Neuropathology, Saarland University, Homburg, Germany; 16 Department of Radiation Oncology, University Clinic Heidelberg, Heidelberg University, Heidelberg, Germany; 17 Division of Experimental Neurosurgery, Neurosurgery Clinic, University Clinic Heidelberg, Heidelberg University, Heidelberg, Germany; 18 Hopp Children’s Cancer Center Heidelberg (KiTZ), German Cancer Research Center (DKFZ) and German Cancer Consortium (DKTK), Division of Pediatric Neurooncology, Heidelberg University Hospital, Department of Pediatric Hematology and Oncology, National Center for Tumor Dieseases (NCT), Heidelberg, Germany; 19 Department of Neurosurgery, University Hospital Tübingen, Tübingen, Germany; 20 Present Address: Neurosurgery Clinic, Ludwig Maximilians University (LMU), Munich, Germany

**Keywords:** Drug development, Predictive markers

## Abstract

Advances in molecular understanding and diagnostic precision of glioblastoma enable the identification of key genetic alterations in a timely manner and, in principle, allow treatments with targeted compounds based on molecular markers. Here we report the results of the phase 1/2 umbrella trial NCT Neuro Master Match (N^2^M^2^), which evaluated targeted treatments in 228 patients with newly diagnosed glioblastoma without O6-methylguanine DNA-methyltransferase promoter hypermethylation. Stratification for treatment was conducted by a trial-specific molecular tumor board across five subtrials, each evaluating a targeted therapy—alectinib, idasanutlin, palbociclib, vismodegib or temsirolimus—selected according to the best-matching molecular alteration. Patients without matching alterations were randomized between subtrials without strong biomarkers using atezolizumab and asunercept, and the standard of care (SOC), temozolomide. All received radiotherapy. The primary endpoints were dose-limiting toxicities (phase 1) and progression-free survival at 6 months (PFS-6; phase 2). Secondary endpoints included safety and tolerability, as well as overall survival (OS). The subtrials for alectinib and vismodegib did not open as they did not have matching patients. The idasanutlin subtrial (*n* = 9) was terminated early at the discretion of the manufacturing company. The temsirolimus subtrial (*n* = 46) demonstrated a PFS-6 of 39.1% and median OS of 15.4 months in patients with activated mammalian target of rapamycin (mTOR) signaling compared to a PFS-6 at 18.5% in the SOC group (*n* = 54), meeting the primary endpoint. The atezolizumab (*n* = 42), asunercept (*n* = 26) and palbociclib (*n* = 41) subtrials did not meet the primary endpoint for efficacy. The safety signals of N^2^M^2^ match prior experiences with the drugs in quality and quantity; no relevant negative interaction with the parallel radiotherapy was noted. The results of the N^2^M^2^ trial support further investigation of temsirolimus in addition to radiotherapy in patients with newly diagnosed glioblastoma with activated mTOR signaling. ClinicalTrials.gov registration: NCT03158389.

## Abstract (web summary)

In a phase 1/2a umbrella trial in patients with glioblastoma matching targeted therapies according to tumor molecular profiling, temsirolimus plus radiotherapy improved PFS compared to standard of care in patients with phospho-mTOR activation.

## Main

The current standard of care (SOC) postoperative treatment for patients with a newly diagnosed isocitrate dehydrogenase (IDH) wild-type glioblastoma^1^ comprises 6-week radiotherapy combined with oral temozolomide (TMZ), followed by a maintenance phase with 6–12 28-day cycles of adjuvant TMZ^2^.

O6-methylguanine DNA-methyltransferase (*MGMT*) promoter methylation may guide treatment decisions regarding the use of alkylating agent chemotherapy in patients with IDH wild-type glioblastoma^2,3^. Patients with glioblastoma without *MGMT* promoter hypermethylation are unlikely to benefit from TMZ^4^.

Trials aiming at replacing TMZ with targeted agents in not molecularly selected patient populations have failed to demonstrate relevant benefit to date^5–8^. Advances in molecular understanding of glioblastoma and technological development allow rapid and precise molecular diagnostics. Thus, in principle, treatment with targeted compounds based on molecular markers could be integrated into first-line treatment. As these studies may withhold TMZ in at least one study arm for the poorly responding *MGMT* not hypermethylated patients, accurate determination of *MGMT* promoter methylation status is crucial to avoid withholding TMZ in patients that might benefit from this drug^9^. Further, well-considered allocation of patients to clinical trials based on molecular characteristics of the tumor, as well as necessary retrospective validation of potential biomarkers, is essential in a clinical setting.

Potential candidate molecular lesions that may guide treatment include EGFR pathway activation—despite lack of convincing clinical data to support efficacy of targeting EGFR overexpression or amplification itself^10^—mammalian target of rapamycin (mTOR) phosphorylation^5^, mouse double minute 2 homolog (*MDM2*) amplification or overexpression^11^, as well as RB1 pathway alterations/*CDK4*/*CDK6* amplification^12^ that have a high prevalence but uncertain relevance in glioblastoma or *BRAF* mutation^13^, anaplastic lymphoma kinase (ALK) expression/fusion^14^ or sonic-hedgehog (SHH) overexpression^15^, with low prevalence in glioblastoma but high likelihood of therapeutic relevance if present. Interestingly, as for TMZ^16^, also for other targeted compounds, glioblastoma methylation subclass effects have been described modulating the relevance of the distinct molecular lesion^17^.

For the NCT Neuro Master Match (N^2^M^2^)/Neurooncology Working Group of the German Cancer Society (NOA)-20 umbrella trial, we opted for alectinib, vismodegib, idasanutlin, palbociclib and temsirolimus in the molecularly matched subtrials, while asunercept, atezolizumab and TMZ were evaluated in the randomized nonmatched subtrials. Of note, TMZ served as an internal reference for the accuracy of the assumptions for the primary efficacy endpoint, progression-free survival at 6 months (PFS-6).

N^2^M^2^ was designed to limit entry into five of eight subtrials based on predefined biomarkers assessed during a comprehensive molecular workup, which was determined 4 weeks after surgery and decided upon by a molecular tumor board. Randomization was used in the three remaining subtrials. Depending on the status of the different treatments, a phase 1 component was used to determine the optimal dose in addition to radiotherapy. There was a central assessment of the phase 2a endpoint, PFS-6.

We here report results from the open-label eight-subtrial prospective N^2^M^2^ trial in patients with newly diagnosed IDH wild-type glioblasstoma^1^ without *MGMT* promoter hypermethylation^3^.

## Results

**Fig. 1:**
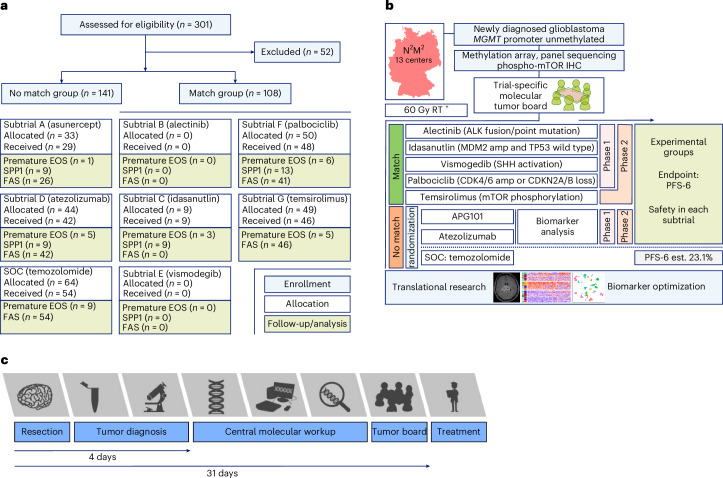
The NCT N^2^M^2^ trial CONSORT overview and concept. **a**, Short CONSORT flow chart. EOS, end of study. **b**, Schematic overview of the trial. The trial was conducted in 13 centers in Germany and molecular tumor board-based allocation was performed into five match and three no-match arms, including one arm with SOC. PFS-6 estimation for SOC (40%) was based on an assumption before trial start and was later corrected to 23.1%. RT, radiotherapy; plus sign indicates that RT was always given in conjunction with one of the subsequently mentioned trial treatments. **c**, The diagnostic workup in the N^2^M^2^ trial.

N^2^M^2^ is an umbrella trial that tests multiple targeted therapies within a single cancer type, here patients with newly diagnosed glioblastoma. The umbrella allows for uniform trial conduct and evaluation in each subtrial. Umbrella trials assign patients to treatments based on specific genetic mutations or biomarkers found in their tumors. The design allows for the simultaneous evaluation of several drugs or interventions tailored to different molecular subgroups within one disease, aiming to match patients to the most appropriate therapy according to their individual tumor characteristics. N^2^M^2^ is divided into a discovery and a treatment part. Discovery includes broad molecular neuropathological diagnostics to detect predefined biomarkers for targeted treatments. Molecular diagnostics (as detailed below) and bioinformatic evaluation were performed within 4 weeks, allowing a timely initiation of postoperative treatment^18^. Stratification for treatment was performed in a trial-specific molecular tumor board in five subtrials, including alectinib, idasanutlin, palbociclib, vismodegib and temsirolimus as targeted therapies, according to the best-matching molecular alteration. Patients without matching alterations were randomized via randomizer.at to subtrials lacking strong biomarkers—evaluating atezolizumab and asunercept (APG101)—or to SOC treatment, TMZ (Fig. 1b).

**Extended Data Fig. 1:**
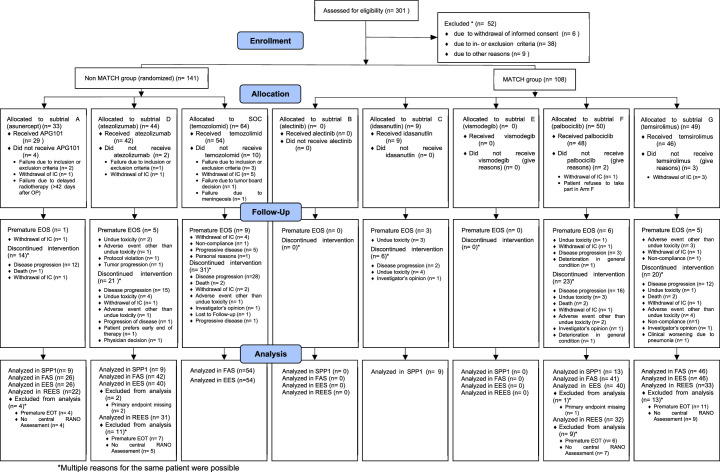
CONSORT diagram. Recruiting trial sites. In total, 301 patients were enrolled in 13 neurooncology working group (NOA) trial sites across Germany.

**Extended Data Fig. 2:**
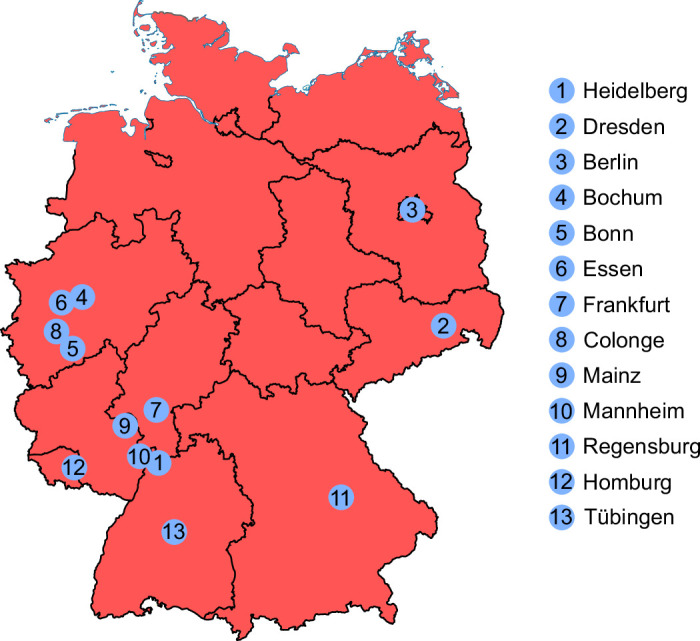
Trial sites. Drawn map of Germany depicting the N^2^M^2^ sites.

From May 2018 through July 2022, 301 patients were enrolled, 249 allocated to treatments and 228 treated (Fig. 1a and Extended Data Fig. 1) at 13 German sites of the NOA (Extended Data Fig. 2). Patient disposition of the treatment cohort is summarized in Table 1 and the whole screening cohort is summarized in Supplementary Table 1.

**Table 1:** Patient characteristics (treatment population, *n* = 228)

Characteristics	All patients	Male	Female
Sex, *n* (%)			
Male	145 (63.6)	145 (100)	0
Female	83 (36.4)	0	83 (100)
Age continuous (years), mean (s.d.)	58.5 (9.26)	58.3 (9.14)	58.9 (9.51)
BMI (kg m^−^^2^), mean (s.d.)^a^	26.6 (4.61)	27.3 (4.21)	25.5 (5.04)
Height (cm), mean (s.d.)^a^	174.1 (8.89)	178.6 (6.89)	166.2 (5.97)
Weight (kg), mean (s.d.)^a^	81.0 (16.88)	87.2 (14.97)	70.3 (14.52)
Age categorical (years), *n* (%)			
18–44	16 (7.0)	9 (6.2)	7 (8.4)
45–64	146 (64.0)	97 (66.9)	49 (59.0)
≥65	66 (28.9)	39 (26.9)	27 (32.5)
Ethnic group, *n* (%)			
Caucasian/white	225 (98.7)	142 (97.9)	83 (100)
Asian	3 (1.3)	3 (2.1)	0
Karnofsky performance status at baseline, *n* (%)			
70	13 (5.7)	6 (4.1)	7 (8.4)
80	59 (25.9)	39 (26.9)	20 (24.1)
90	104 (45.6)	61 (42.1)	43 (51.8)
100	52 (22.8)	39 (26.9)	13 (15.7)
Resection status, *n* (%)^b^			
Biopsy	11 (4.8)	4 (2.8)	7 (8.4)
Partial resection	83 (36.4)	51 (35.2)	32 (38.6)
Complete resection	144 (63.2)	93 (64.1)	51 (61.4)
*MGMT* promoter methylation, median (range)	2% (1–8%)	2% (1–8%)	2% (1–7%)
Glioblastoma methylation classes, *n* (%)			
Mesenchymal	66 (28.9)	41 (28.3)	25 (30.1)
RTK1	58 (25.4)	43 (29.7)	15 (18.1)
RTK2	83 (36.4)	47 (32.4)	36 (43.4)
Other	21 (9.2)	14 (9.7)	7 (8.4)

^a^For the overall two patients, neither weight nor height was documented.

^b^Some patients had both (partial or complete) resection and biopsy or only resection. One (male) patient had only a biopsy.

BMI, body mass index.

For validation of the null hypothesis, patients treated according to SOC were observed for the efficacy endpoint PFS-6. Treatment according to SOC comprises radiotherapy at 60 Gy in 2 Gy fractions plus concomitant TMZ chemotherapy (75 mg m^−^^2^ body surface) followed by six cycles of TMZ maintenance therapy (150–200 mg m^−^^2^ body surface). The null hypothesis is supposed to give the PFS-6 rate of SOC. Originally, the rate was set to 40%. Based on data collected from the first 26 patients with 6 responders, the Data and Safety Monitoring Committee, prospectively and adherent to the protocol for the first interim analysis, suggested correcting the rate (and thus *P*_0_) to 23.1%.

### Discovery: molecular assessments

Median time from resection to diagnosis was 4 days (range = −37 to 37) and time from resection to trial-specific molecular tumor board (MTB) decision was 31 days (range = 15–42; Fig. 1c).

The diagnosis of glioblastoma, IDH wild type, was assessed locally according to the WHO classification^1^ and confirmed with central methylation array profiling. Most of the tumors belong to the three main glioblastoma methylation classes, receptor tyrosine kinase (RTK)1 (58/228, 25.4%), RTK2 (83/228, 36.4%) and mesenchymal (66/228, 28.9%; Table 1). The remaining tumors were classified into less common tumor methylation classes or as control tissue based on low tumor content.

Tumor samples were tested for *MGMT* promoter methylation at the local site, and a nonhypermethylated *MGMT* promoter was confirmed centrally in Heidelberg. *MGMT* pyrosequencing was the primary assay for *MGMT* testing and a cutoff ≤8% was used to verify *MGMT* promoter nonhypermethylated tumors. The median *MGMT* promoter methylation value in the treatment cohort was 2% (range = 1–8%). Results from pyrosequencing were available in 225 of 228 cases (98.7%). In the remaining patients, methylation array was used to confirm a nonhypermethylated *MGMT* promoter. In 12 of 228 (5.3%) patients, *MGMT* promoter was methylated according to methylation array; however, inclusion was based on pyrosequencing as the gold standard in these patients^19^. Median pyrosequencing value was 4% (range = 1–7%) in samples with *MGMT* methylation as assessed by methylation array.

**Fig. 2:**
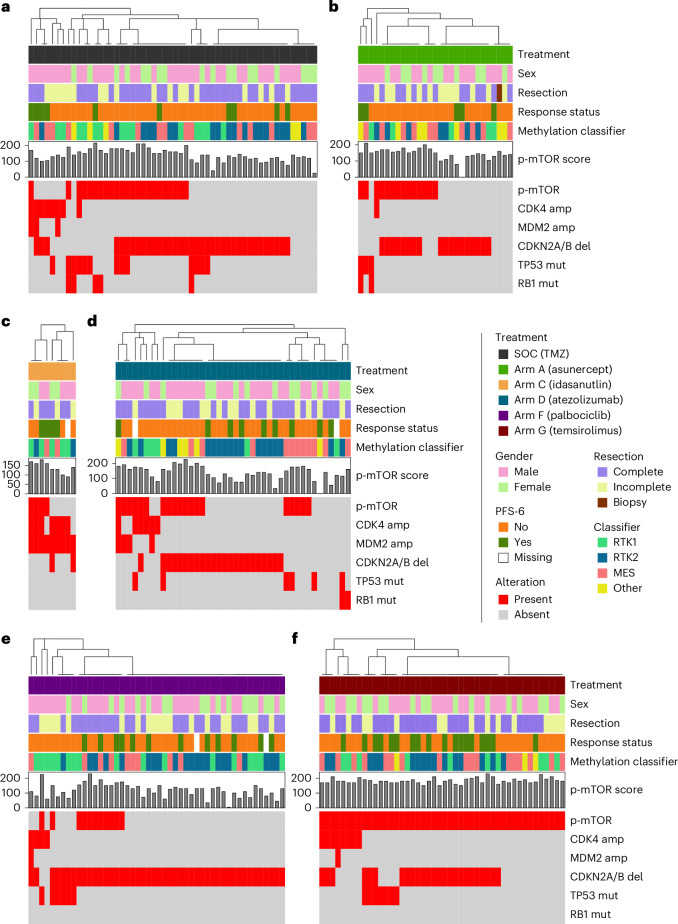
Discovery—molecular assessments and trial arm allocation. **a**–**f**, SOC (TMZ; **a**), arm A (asunercept; **b**), arm C (idasanutlin; **c**), arm D (atezolizumab; **d**), arm F (palbociclib; **e**) and arm G (temsirolimus; **f**) are shown. amp, amplified; del, deleted; mut, mutated; MES, mesenchymal.

Patients in the palbociclib arm were included based on CDK4 amplification in 4 of 48 cases, CDKN2A/CDKN2B deletion in 46 of 48 cases. Two of the treated patients had both CDK4 amplification and CDKN2A/CDKN2B deletion (Fig. 2). Activation of mTOR was defined as a cytoplasmic H-score of 150 or above with a maximum of 20% negative tumor cells based on phospho-mTOR immunohistochemistry (IHC). The median phospho-mTOR score for patients treated with temsirolimus was 180 (range = 150–230). Figure 1a indicates that a total of 141 patients were randomized to the nonmatched trials, whereas 108 patients underwent matching to one of the subtrials.

### Treatment

**Fig. 3:**
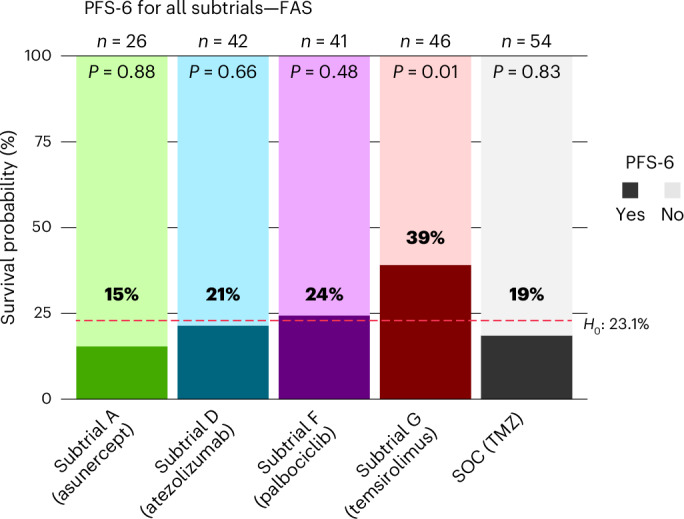
Primary endpoint phase 2a N^2^M^2^. Treatment—PFS-6 results of the N^2^M^2^ trial.

**Extended Data Fig. 3:**
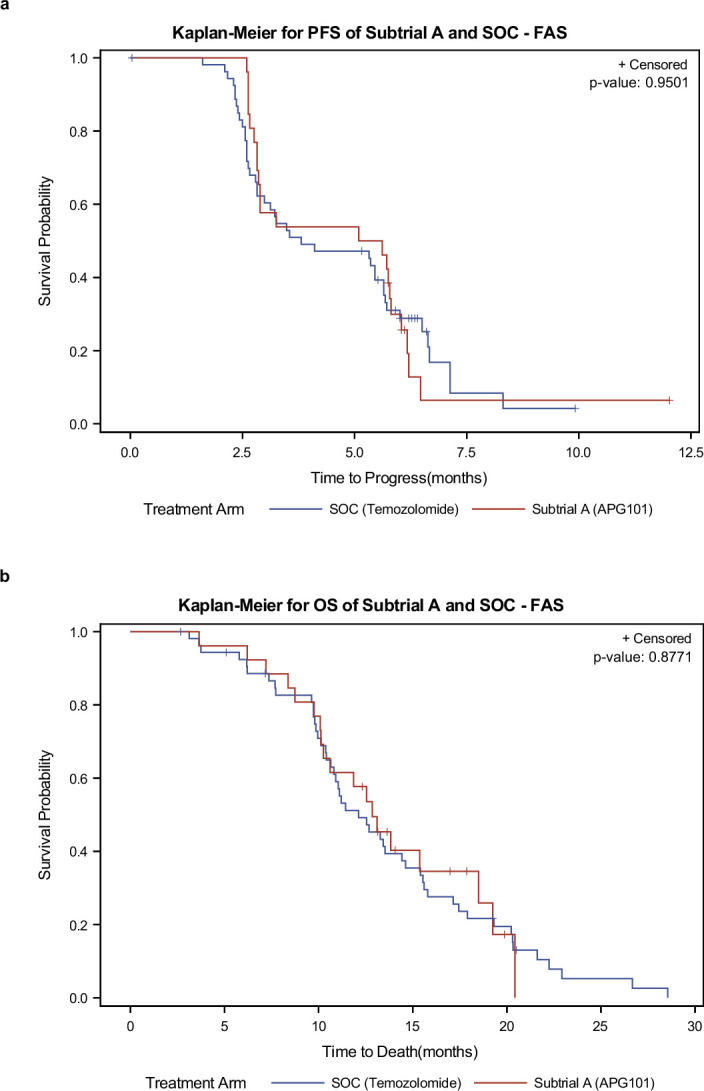
Survival results of subtrial A (asunercept) in the FAS. **a**, Kaplan-Meier plot for PFS of subtrial A (n = 26) and SOC (n = 54). P-value shows result of log-rank test. **b**, Kaplan-Meier plot for OS of subtrial A (n = 26) and SOC (n = 54). P-value shows result of log-rank test.

Determination of the safe combination dose of intravenous asunercept, starting with 600 mg per week with an escalation step of 200 mg, that is, D1 = 600 mg (*n* = 3 patients), D2 = 800 mg (*n* = 6 patients), in conjunction with radiotherapy, revealed 800 mg per week to be safe. No dose-limiting toxicity (DLT) or regimen-limiting toxicity (RLT) were reported in phase 1 (Supplementary Table 2). The asunercept subtrial showed a PFS-6 of 15.4% (4/26; *P* = 0.8825; Fig. 3), a median PFS of 5.4 months (95% confidence interval (CI) = 2.8–5.8) and a median OS of 13.0 months (95% CI = 10.1–19.3; Extended Data Fig. 3), and was closed for futility at the second interim analysis. No DLT or RLT were observed (0/26, 95% Clopper–Pearson CI = 0, 0.132), thus tolerability was confirmed (Table 2).

**Table 2:** Safety results for patients in the FAS

Related AE	Arm A—asunercept (*n* = 26) *n* (%)	Arm D—atezolizumab (*n* = 42) *n* (%)	Arm F—palbociclib (*n* = 41) *n* (%)	Arm G—temsirolimus (*n* = 46) *n* (%)
Any AE	10 (38.5)	34 (81.0)	36 (87.8)	40 (87.0)
Any SAE	0 (0)	9 (21.4)	6 (14.6)	10 (21.7)
Any severe AE (grade 3 or 4)	0 (0)	10 (23.8)	11 (26.8)	16 (34.8)
Any DLT/RLT	0 (0)	10 (23.8)	10 (24.3)	16 (34.8)
Discontinued study drug due to AE	0 (0)	7 (16.7)	2 (4.9)	4 (8.7)
Dose reduction or temporary discontinuation due to AE	0 (0)	5 (11.9)	9 (22.0)	19 (41.3)
AE resulting in death	0 (0)	0 (0)	1 (2.4)	0 (0)

AE, adverse event; SAE, serious adverse event.

**Extended Data Fig. 4:**
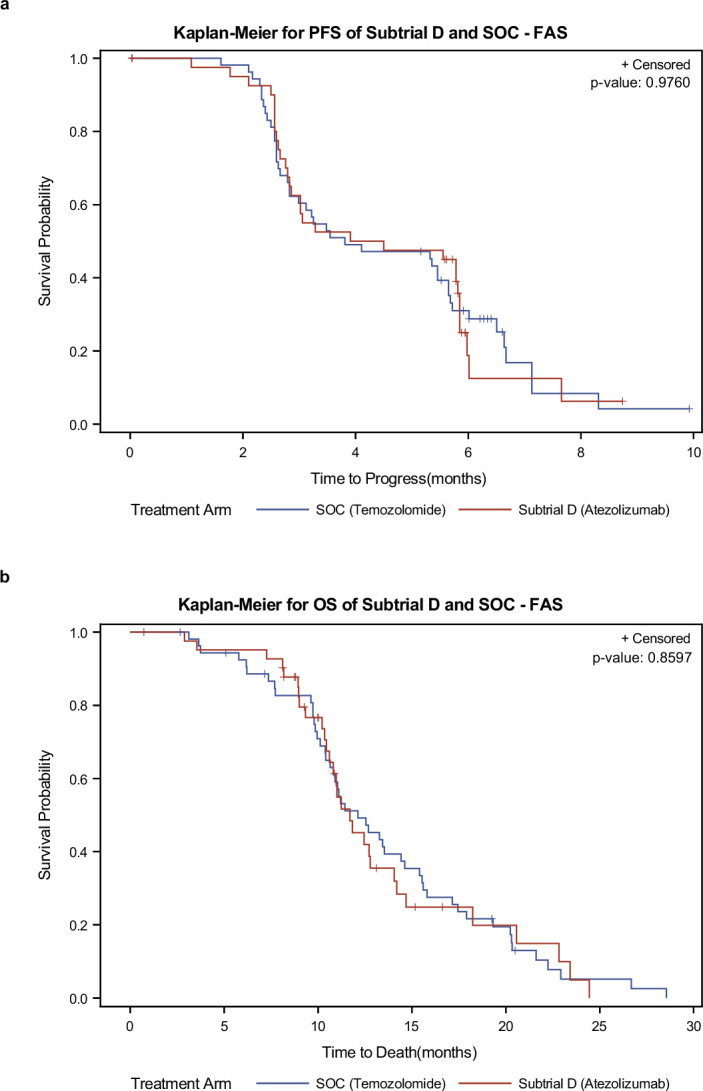
Survival results of subtrial D (atezolizumab) in the FAS. **a**, Kaplan-Meier plot for PFS of subtrial D (n = 42) and SOC (n = 54). P-value shows result of log-rank test. **b**, Kaplan-Meier plot for OS of subtrial D (n = 42) and SOC (n = 54). P-value shows result of log-rank test.

Atezolizumab was administered intravenously at 1,200 mg every 3 weeks in conjunction with radiotherapy (*n* = 9 patients). DLTs were reported in two patients (2/9, 22.2%); no RLTs were reported. The dose was evaluated as safe to continue in phase 2a (Supplementary Table 2). The atezolizumab subtrial showed a PFS-6 of 21.4% (9/42; *P* = 0.660; Fig. 3), a median PFS of 4.2 months (95% CI = 2.8–5.8) and a median OS of 11.7 months (95% CI 10.4–14.1; Extended Data Fig. 4). A total of 23.8% (10/42) of the patients experienced either DLT or RLT (95% CI = 0.121, 0.395), thus the rate is below the unacceptable rate, but tolerability cannot be confirmed according to the CI (Table 2). Most DLTs/RLTs were hepatobiliary disorders (4/42, 9.5%).

For TMZ, no phase 1 part has been performed due to existing data for the combination with radiotherapy^5^. The TMZ subtrial showed a PFS-6 of 18.5% (10/54 patients, 95% CI of all patients (including patients with missing response status) = 0.0925, 0.3143%; *P* = 0.831; Fig. 3). Nine of them were confirmed by central Response Assessment in Neuro-Oncology (RANO) assessment. The best available assessment was ‘stable disease’. Median PFS was 3.8 months (95% CI = 2.8–5.7), and a median OS of 12.1 months (95% CI = 10.6–14.6) was observed. The subtrials for alectinib and vismodegib were closed prematurely since no molecularly matching patients had been accrued.

The idasanutlin subtrial was closed before finding the optimal dose in nine patients at the discretion of the company providing the study drug. Eight patients were available for response assessment, and the PFS-6 rate was 50% (4/8, 95% CI = 15.7–84.3%). The combined DLT/RLT rate was 55.5% (5/9; Supplementary Table 2). DLT/RLTs included leucopenia, neutropenia and thrombocytopenia.

**Extended Data Fig. 5:**
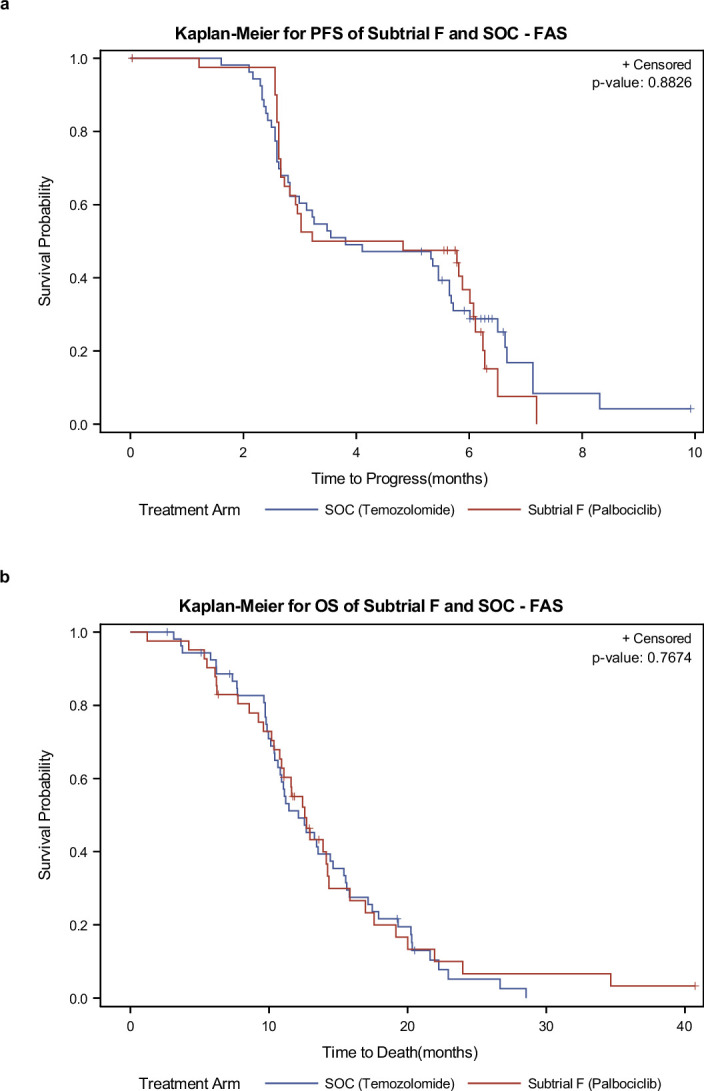
Survival results of subtrial F (palbociclib) in the FAS. **a**, Kaplan-Meier plot for PFS of subtrial F (n = 41) and SOC (n = 54). P-value shows result of log-rank test. **b**, Kaplan-Meier plot for OS of subtrial F (n = 41) and SOC (n = 54). P-value shows result of log-rank test.

**Extended Data Fig. 6:**
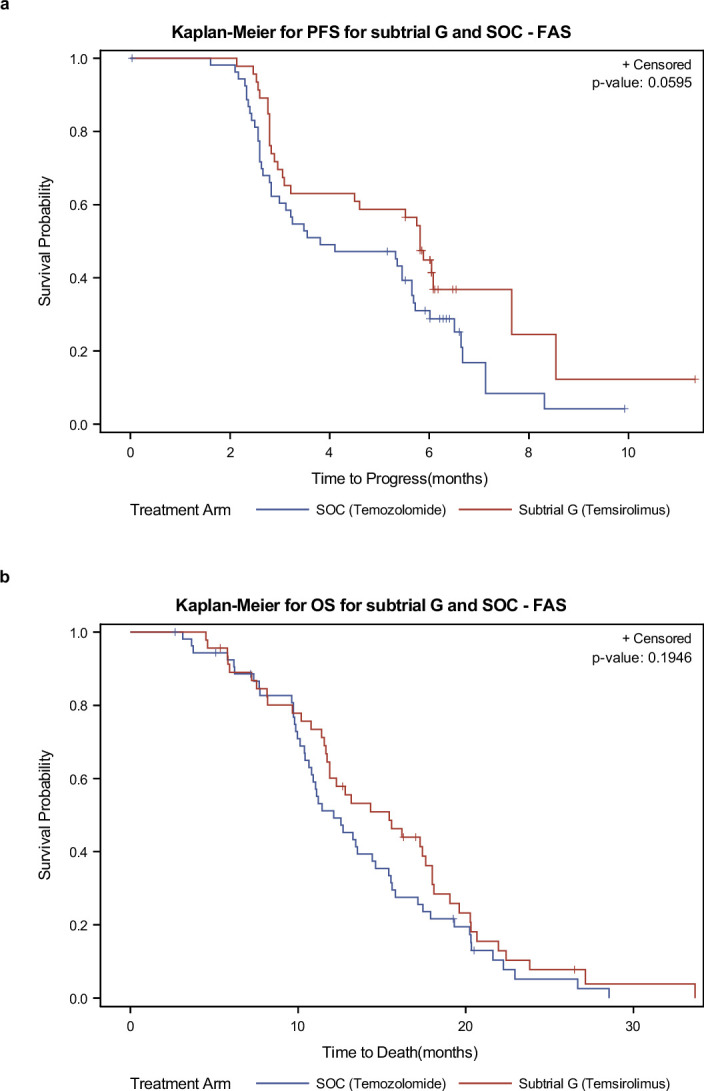
Survival results of subtrial G (temsirolimus) in the FAS. **a**, Kaplan-Meier plot for PFS of subtrial G (n = 46) and SOC (n = 54). P-value shows result of log-rank test. **b**, Kaplan-Meier plot for OS of subtrial G (n = 46) and SOC (n = 54). P-value shows result of log-rank test.

In the palbociclib subtrial, the compound was administered initially at 75 mg (*n* = 1) with dose escalation steps to 100 mg (*n* = 6) and 125 mg (*n* = 6) during combination with radiotherapy and at 125 mg in adjuvant monotherapy on 21 consecutive days of a 28-day cycle. A total of 83.3% (5/6) of patients receiving a dose of 125 mg in combination with radiotherapy experienced either DLT or RLT (95% CI = 0.359, 0.996); thus, the probability is high that the tolerability is unacceptable. For the dose of 75 mg, neither DLTs nor RLTs were observed (0/1, 95% CI = 0, 0.95). One DLT was observed in patients receiving 100 mg (1/6, 16.7%, 95% CI = 0.004, 0.641); thus, this dose has been assessed to be safe in combination with radiotherapy to proceed to phase 2a (Supplementary Table 2). The palbociclib subtrial with patients demonstrating CDK4 amplification or CDKN2A/CDKN2B codeletion showed a PFS-6 of 24.4% (10/41; *P* = 0.4823; Fig. 3), a median PFS of 4.0 months (95% CI = 2.7–6.0) and a median OS of 12.6 months (95% CI = 10.8–14.2; Extended Data Fig. 5). A total of 26.8% (11/41) of the patients experienced either DLT or RLT (95% CI = 0.142, 0.429), thus the rate is below the unacceptable rate, but tolerability cannot be confirmed according to the CI (Table 2). Most RLTs were related to hematologic toxicity (7/41, 17.1%) and infections (2/41, 4.9%). For temsirolimus, no phase 1 part has been performed due to existing data for the combination with radiotherapy^5^. The temsirolimus subtrial with patients demonstrating phospho-mTOR activation showed a PFS-6 of 39.1% (18/46; *P* = 0.0109, 95% CI = 25.1–54.6%); Fig. 3), a median PFS of 5.8 months (95% CI = 3.1–7.7) and a median OS of 15.4 months (95% CI = 11.8–18.0; Extended Data Fig. 6). Baseline characteristics did not differ between the SOC and temsirolimus subtrial, except for phospho-mTOR score (Supplementary Table 3). The observed RLT-rate is 34.8% (16/46, 95% CI = 0.214, 0.502), which is insignificantly above the predefined unacceptable rate for RLTs of 30% (Table 2). The majority of RLTs were infections (8/46, 17.4%), hematologic toxicities (3/46, 6.5%) and gastrointestinal disorders (3/46, 6.5%). Most RLTs had severity grade 3, and one RLT had severity grade 4. No RLTs resulted in death.

As a sensitivity analysis, the same calculations were performed on the efficacy evaluable set (EES; Supplementary Table 4). For SOC, temsirolimus and asunercept, full analysis set (FAS) and EES are equal, thus sensitivity analysis does not differ from the primary analysis.

### Clinical, molecular and imaging assessments

In patients in the SOC arm, a low *MGMT* promoter methylation pyrosequencing value of ≤2% was identified in 43 of 53 patients with one patient with missing information due to technical reasons. Of these, 6 of 43 (14.0%) patients had stable disease, whereas 4 of 10 (40%) patients with *MGMT* promoter methylation in the range of 3–7% had stable disease (Table 3). Median PFS was 3.7 months versus 5.8 months, and OS was 11.2 months versus 16.3 months. Comparisons did not reach significance and had been prespecified as exploratory endpoints.

**Table 3:** Molecular response data for patients in the FAS

	PFS-6	*P* value^a^	PFS	OS
SOC (*n* = 53)^b^				
MGMT methylation ≤ 2%	6/43 (14.0)	0.08	3.7 (2.6–5.5)	11.2 (10.1–13.4)
MGMT methylation > 2%	4/10 (40.0)		5.8 (2.6–NA)	16.3 (7.4–20.3)
Asunercept (*n* = 26)				
CD95 methylation low	1/5 (20.0)	1.00	6.0 (2.8–NA)	12.8 (9.8–NA)
CD95 methylation high	3/21 (14.3)		3.3 (2.8–5.8)	13.1 (10.1–19.3)
Temsirolimus (*n* = 46)				
mTOR low—other	22/98 (22.4)	NA	3.2 (2.9–5.8)	12.5 (10.8–13.9)
mTOR high—other	11/65 (16.9)	NA	5.5 (3.3–5.7)	12.5 (11.1–14.2)
mTOR high—SOC	3/23 (13.0)	0.03	4.6 (3.0–5.7)	11.1 (9.7–15.5)
mTOR high—temsirolimus	18/46 (39.1)		5.8 (3.1–7.7)	15.4 (11.7–18.0)

^a^*P* value is based on PFS-6 values calculated with exact Fisher test.

^b^For one patient in the SOC arm, information on MGMT pyrosequencing is missing.

NA, not applicable.

CD95 promoter methylation had previously been proposed as a potential biomarker for asunercept treatment together with irradiation in progressive glioblastoma and was prespecified as an exploratory endpoint for N^2^M^2^ (ref. ^20^). However, there was no association between CD95 CpG2 (cg10161121) and response to asunercept in this trial (Table 3).

A single primary hypermutation phenotype based on an *MSH6* mutation was observed in one tumor of a patient treated in the temsirolimus arm. Therefore, no association between hypermutation and atezolizumab response could be further explored.

**Extended Data Fig. 7:**
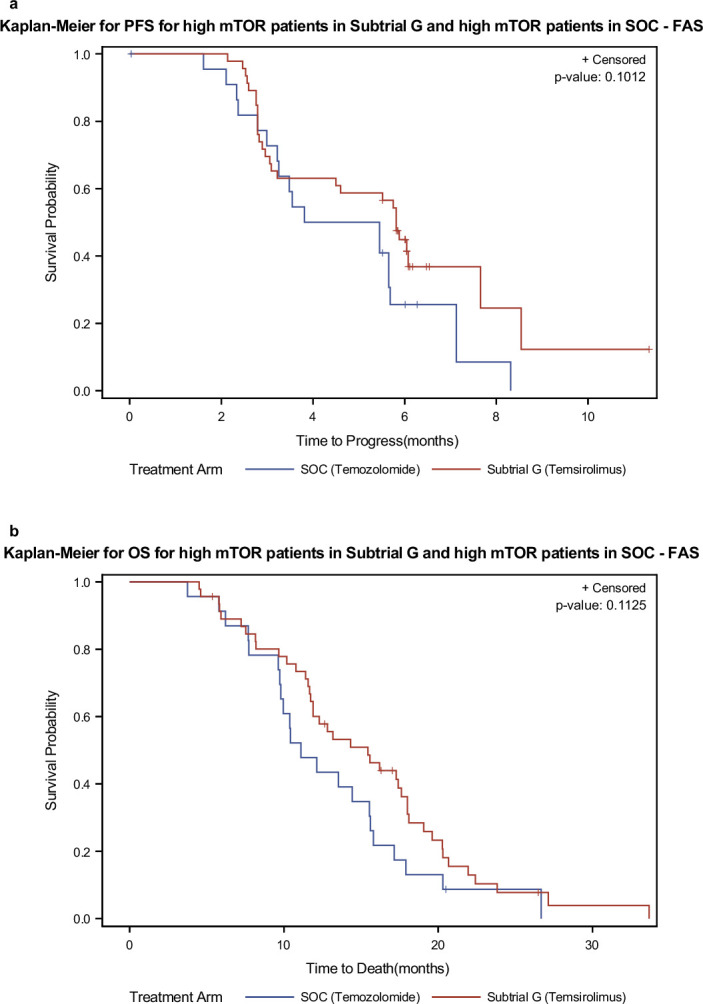
Survival results for comparison of subtrial G (temsirolimus) to phospho-mTOR high patients in SOC. **a**, Kaplan-Meier plot for PFS of subtrial G (n = 46) and phospho-mTOR high patients in SOC (n = 23). P-value shows result of log-rank test. **b**, Kaplan-Meier plot for OS of subtrial G (n = 46) and phospho-mTOR high patients in SOC (n = 23). P-value shows result of log-rank test.

The phospho-mTOR score alone has previously not been prognostic and assessment of clinical impact was prespecified as an exploratory endpoint for N^2^M^2^ (ref. ^5^). To exclude the prognostic value of phospho-mTOR in this trial, thus potentially explaining the increased response rates in the temsirolimus arm, patients from the FAS dataset treated with drugs other than temsirolimus were analyzed (*n* = 163). In this subgroup, 98 tumors had low phospho-mTOR (H-score <150 or >20% negative) and 65 had high phospho-mTOR (H-score ≥150 and ≤20% negative). PFS-6 rates were 22.4% (22/98) in the phospho-mTOR low group and 16.9% (11/65) in the phospho-mTOR high group; therefore, they were lower than in the temsirolimus-treated group (39.1%, 18/46), where all tumors met the inclusion criterion for high phospho-mTOR. OS was both 12.5 months in the phospho-mTOR low and phospho-mTOR high group (Table 3). As an exploratory analysis, we compared survival rates of patients in the temsirolimus subtrial to phospho-mTOR high patients in SOC. PFS-6 rates were higher in the temsirolimus subtrial (39.1%, 18/46) compared to SOC 13% (3/23, *P* = 0.03). PFS was 5.8 months versus 4.6 months, and OS was 15.4 months versus 11.1 months (Table 3 and Extended Data Fig. 7).

**Extended Data Fig. 8:**
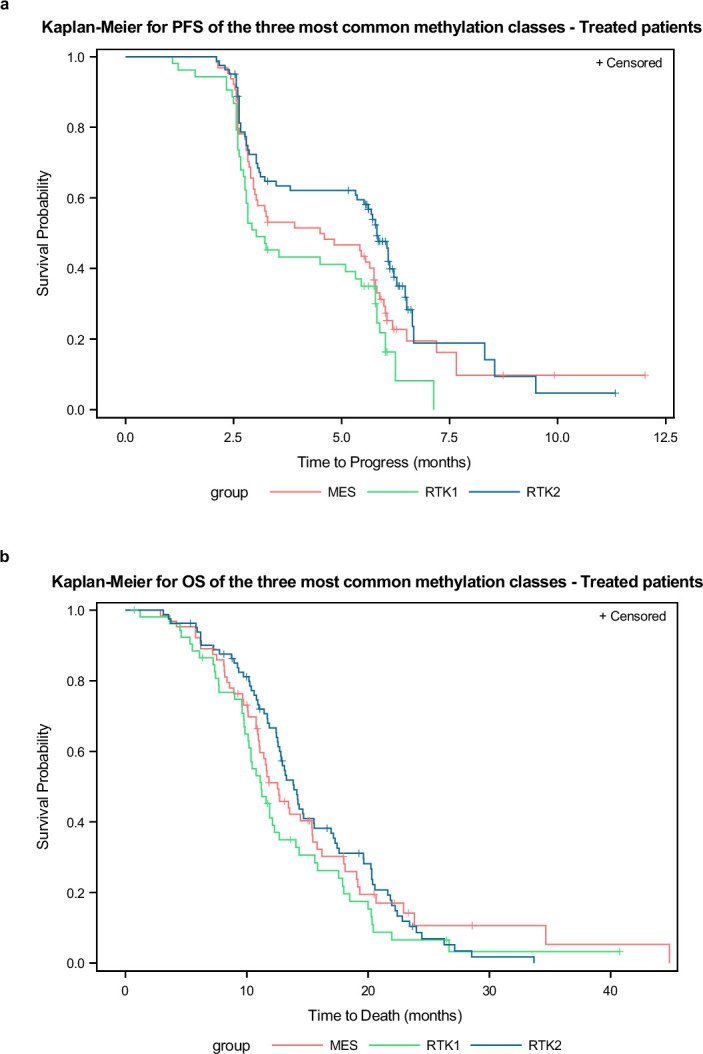
Survival results for comparison of the three main methylation classes in treated patients. **a**, Kaplan-Meier plot for PFS in patients with RTK1 (n = 58), RTK2 (n = 83) and MES (n = 66) tumors. **b**, Kaplan-Meier plot for OS in patients with RTK1 (n = 58), RTK2 (n = 83) and MES (n = 66) tumors.

Patients with RTK2 methylation phenotype tumors had a better prognosis compared to RTK1/MES in previous trials^16,21^. PFS-6 rates in the N^2^M^2^ treatment dataset were 10 of 58 (17.2%) for RTK1, 26/83 (31.3%) for RTK2 and 16 of 66 (24.2%) for MES. Similarly, there is a longer PFS for patients with RTK2 tumors; however, no noteworthy difference in OS was observed (Extended Data Fig. 8). Comparisons of methylation classes had been prespecified endpoints.

MRI was performed according to a prespecified protocol. The trial included *post hoc* central reference MRI evaluation for the PFS-6 endpoint. Treatment was stopped in 20 of 209 (9.6%) patients of the FAS dataset in week 12 based on a local assessment of progression on MRI, whereas subsequent RANO evaluations assessed suspected pseudoprogression or stable disease. These cases were present in the following subtrials: asunercept (2/26, 7.7%), atezolizumab (5/42, 11.9%), palbociclib (2/41, 4.9%), temsirolimus (2/46, 4.3%) and SOC (9/54, 16.7%).

## Discussion

N^2^M^2^/NOA-20 allowed for elaborate molecular testing to be integrated into the treatment decision for patients with newly diagnosed glioblastoma. It determined in a very efficient way the clinical activity of temsirolimus in patients with tumors harboring an activated mTOR signaling pathway, although this is not prognostic without mTOR inhibition. We observed a lack of clinical potential for asunercept and atezolizumab in not molecularly selected patients and palbociclib in molecularly selected patients, respectively. The effect in the temsirolimus arm needs to be balanced with the toxicity and the once-weekly need for intravenous treatment in this subtrial. However, whether this would be different with another mTOR inhibitor, that is, everolimus, which is approved for the treatment of SEGA^22^, would need further clinical investigation.

This umbrella trial has been effective in demonstrating the feasibility of integrating high-throughput molecular diagnostics into the first-line treatment of patients with glioblastoma without undue delay in treatment initiation. There has been a predefined set of molecular assessments that were completed at a median of 26 days during this trial. With a weekly molecular tumor board, this allowed rapid decisions and initiation of treatments.

There are two principal options to perform a trial with molecular targeted agents—(1) to limit treatment to the best molecularly matching patients and thereby to enhance the likelihood of a positive outcome or (2) to allow for all-comers to be treated and only use the molecular data to evaluate molecular parameters predictive of outcome post hoc. The first option limits the discovery of new potential molecular parameters for a given treatment and avoids randomization. The second option may need a large number of patients to find suitable molecular parameters and has not been particularly successful in the past. For N^2^M^2^, we opted to assign molecularly matching patients to five subtrials and to use randomization for patients without validated biomarkers to the remaining three subtrials, including the SOC.

For the temsirolimus treatment phospho-mTOR activation, determined as H-score (nuclear and cytoplasmatic) had been proposed as a biomarker in the European Organization for Research and Treatment of Cancer (EORTC)-26082 trial^5^. N^2^M^2^ was set up to confirm this association and set the scores at a minimum of 150 (cytoplasmatic, scale from 0 to 300) with a maximum of 20% negative tumor cells. Especially for patients without hypermethylation at the *MGMT* promoter^9^, the use of temsirolimus may be an option at first-line, although some patients suffer from unwanted effects that limit drug exposure. Further, there are considerations of even higher doses as in mantle cell lymphoma^23^. Since the H-score for phospho-mTOR is also elevated in more aggressive or recurrent disease^24^, mTOR inhibition may also be suitable for salvage treatment. Notably, the phospho-mTOR score alone was not prognostic. It seems plausible that an IHC-based score is a good option for broad application. On the other hand, work towards establishing a stronger quantitative assay using phosphoprotein levels will help to define a threshold and to elucidate the phospho-mTOR–efficacy relationship.

N^2^M^2^ confirms a lack of activity of TMZ in patients with a nonhypermethylated *MGMT* promoter^4^, which is relevant for two reasons. First, the uncertainty on limited, but not absent activity raised by contemporary trials^10^, is most likely due rather to insufficient scrutiny in the determination of methylation status than to residual activity. For elderly patients, use of a more stringent cutoff for residual activity of the PCR determining *MGMT* promoter methylation demonstrated that patients with no/minimal promoter methylation derived no benefit from TMZ in the reanalysis of three randomized trials^9^. Hence, it is well possible to leave out the drug in the trial setting as well as in elderly patients. Second, the present trial now reopens the question whether TMZ also lacks activity in all glioblastoma patients with *MGMT* promoter methylation <8% determined by pyrosequencing. Whether patients with a glioblastoma harboring a nonhypermethylated *MGMT* promoter should be offered alternative treatments in addition to radiotherapy already at first-line treatment is a relevant question, when considering the availability of molecular testing and a critical lack of options.

Limitations of N^2^M^2^ include the selection of drugs, which could have been more extensive, such as BRAF and NTRK inhibitors, to determine their role in the first-line treatment of adults with glioblastoma. Also, the mix of matched and randomized subtrials (Fig. 1) was a compromise as asunercept and atezolizumab had had a scientific rationale for CD95 promoter methylation^20^ or PD1 expression/high mutational burden, respectively. However, the trial group considered the data from other solid tumors not to be sufficiently mature. With the data from N^2^M^2^, we feel confident that we have not missed any known matching parameters, as neither subtrial showed differences in PFS according to any of the assessed molecules. Despite the overall negative signal, there were a few responding patients in both subtrials, who may be further analyzed.

In contrast to the positive data from breast cancer, the CDK4/CDK6 and CDKN2A/CDKN2B alterations were not sufficient to predict the success of palbociclib. From the trial data, it is not clear whether this was due to the high incidence of CDKN2A/CDKN2B deletions, which may be rather diagnostic than predictive, or to low blood–brain barrier penetration of palbociclib^25^ and thus limited drug availability in the glioblastoma cells, despite a regularly open blood–brain barrier in glioblastoma, is not.

The lack of molecularly matching patients for alectinib and vismodegib has been even below the expected very low frequency of these patients^26,27^ and clearly calls for more stringent estimation of biomarker frequencies before clinically investigating such treatment arms.

The effect of temsirolimus is robust, given that there is no prognostic impact of the phospho-mTOR score that was used to assign patients in the molecular tumor board, despite the conceptual disadvantage that the SOC with TMZ was not randomized in the subgroup of patients with a high phospho-mTOR score. Randomization was in the group of patients without molecularly matching lesions for one of the active subtrials only. However, the drug was terminated at 6 months, like all other treatments; therefore, limiting the option to produce long-term benefiting patients. Long-term use is also limited by the substantial number of RLTs with this drug.

Finally, PFS-6 as a primary endpoint was a compromise between swift evaluation of the subtrials without interference from a salvage therapy and robustness of the mainly image-based endpoint. To reduce the number of pseudoprogressions, we mandated confirmatory MRI scans at 4 weeks after the initial diagnosis of a suspected pseudoprogression and subjected the local decisions to a central post hoc reading. The range of the rate of relevant discrepancies between the lowest at 4.3% (temsirolimus) and the highest at 16.7% (TMZ) did not statistically impact outcome, but supports the notion that unblinded trials may suffer from bias against the control arm. This is particularly true in the setting of N2M2, which, by inclusion, limited trial entry to a subgroup of patients most likely not benefiting from the present SOC. However, given that N^2^M^2^ did not embark on any antiangiogenic treatments, the assumption that there is a correlation between PFS-6 and OS^28^ was also valid for this trial.

N^2^M^2^ was conducted to understand molecular-driven first-line treatments. The data provided will serve as a resource for further assessments. It should also help to decide which subtrial may be moved forward to phase 3. Based on the efficacy of the temsirolimus subtrial, controlled confirmation of data is warranted to establish an option for a subgroup of patients with glioblastoma without *MGMT* promoter hypermethylation and activity of the mTOR pathway. In addition, there is continued activity to progress molecularly targeted drugs in glioblastoma into later trial phases. GBM AGILE is another platform trial investigating molecularly targeted treatments in unselected patients with newly diagnosed or progressive glioblastoma. In addition to safety and clinical efficacy, it aims at post hoc understanding matching molecular signatures^29^.

## Methods

### Ethical and legal aspects

The trial was conducted in accordance with the standards of Good Clinical Practice, the Declaration of Helsinki and local legal and regulatory requirements. The study protocol has been approved by the lead ethics committee (AFmu-207/2017) in Heidelberg and all regional ethics committees, as well as the competent federal authority (Vorlagennummer 3051/01, Paul-Ehrlich-Institute).

For this trial, the EudraCT 2015-002752-27 has been obtained. The trial has been registered at NCT03158389. Monitoring and pharmacovigilance were performed by the Coordination Center for Clinical Trials (KKS) in Heidelberg.

Patients were enrolled in a two-step consent process. Oral and written explanation of the molecular testing, including interpretation and conduct of the MTB, was provided after surgery, and any trial-specific measures were only started after written informed consent. Consenting for the treatment step in the respective subtrial was done after the MTB decision, before any subtrial-specific process.

### Objective

The primary objective of the phase 1 parts of the trial was dose finding or dose validation. The primary endpoint was DLT. For dose validation, a Bayesian criterion was used for continuous monitoring of dose-limiting toxicities according to Common Terminology Criteria for Adverse Events (CTCAE) version 5.0 (used for subtrial alectinib, atezolizumab and vismodegib).

For dose finding, an accelerated rule-based design was used, where the dosage is escalated to a predefined dose using single-patient cohorts, followed by a classical 3 + 3 design. For temsirolimus, no phase 1 was planned. In the phase 2a trials, PFS-6 was used as the primary endpoint. Secondary endpoints for efficacy were PFS and OS. The methods part of the trial protocol has been published before^18^.

### Patients

Histological diagnosis of a newly diagnosed glioblastoma in an adult with nonhypermethylated *MGMT* promoter determined by one of the accepted methods (qPCR, pyrosequencing, methylation array)^19^ and without mutation of the IDH genes (suitable for all subtrials) and availability of tissue for molecular assessments as well as eligibility for radiotherapy at 60 Gy at a Karnofsky performance status ≥70% have been the main eligibility criteria. Other criteria are mentioned below.

#### Inclusion criteria

1. Open biopsy or resection.
2. The craniotomy or intracranial biopsy site must be adequately healed.
3. Written informed consent.
4. Standard MRI within 72 h (+12 h) postsurgery according to the present national and international guidelines.
5. At least 15 × 8 µm formalin-fixed, paraffin-embedded tumor tissue and 0.2 g tumor tissue (equivalent to at least one pea-sized tumor piece) were freshly cryopreserved during surgery and blood.
6. Life expectancy >6 months.
7. Patients not on steroids or on stable or decreasing steroid levels not exceeding 4 mg d^−1^ dexamethasone (or equivalent doses of other steroids) during the last 3 days before the day of attribution.
8. ANC > 1.5 × 10^9^ l^−1^.
9. Ability of the patient to understand and the willingness to sign written informed consent for study participation.
10. All female patients with reproductive potential must have a negative pregnancy test (serum or urine) within 6 days before the start of therapy. All female patients must be surgically sterile or must agree to use adequate contraception during the period of therapy and 6 months after the end of study treatment, or women must be postmenopausal for at least 2 years. Acceptable methods of contraception comprise barrier contraception combined with a medically accepted contraceptive method for the female patient or female partner of a male patient (for example, intra-uterine device with spermicide, hormonal contraceptive for at least 2 months). Female patients must agree not to donate lactation during treatment and until 6 months after the end of treatment.
11. Male patients who are willing to use contraception (condoms with spermicidal jellies or cream) upon study entry and during the course of the study and 3 months after the end of the study, have undergone vasectomy, or are practicing total abstinence. Sperm donation is not permitted for the same time interval.
12. Other product-specific inclusion criteria are included in the ‘Subtrial-specific eligibility criteria’.

#### Exclusion criteria

General exclusion criteria are mentioned as follows:

1. Participation in other ongoing interventional clinical trials.
2. Insufficient tumor material for molecular diagnostics.
3. Inability to undergo MRI.
4. Abnormal (≥Grade 2 CTCAE v5.0) laboratory values for hematology (Hb, WBC, neutrophils or platelets), liver (serum bilirubin, ALT or AST) or renal function (serum creatinine).
5. Active tuberculosis; HIV infection or active hepatitis B or C infection, or active infections requiring oral or intravenous antibiotics or that can cause a severe disease or pose a severe danger to site staff or lab personnel working on patients’ blood or tissue (for example, rabies).
6. Prior treatment with any of the questioned investigational medicinal products. Any prior anticancer therapy or coadministration of anticancer therapies other than those allowed in this study. A history of low-grade glioma that did not require prior treatment with chemotherapy or radiotherapy is not an exclusion criterion.
7. Immunosuppression, not related to prior treatment for malignancy.
8. History of other malignancies (except for adequately treated basal or squamous cell carcinoma or carcinoma in situ) within the last 5 years, unless the patient has been disease-free for 5 years.
9. Any clinically substantial concomitant disease (including hereditary fructose intolerance) or condition that could interfere with, or for which the treatment might interfere with, the conduct of the study or the absorption of oral medications or that would, in the opinion of the Principal Investigator, pose an unacceptable risk to the patient in this study.
10. Any psychological, familial, sociological or geographical condition potentially hampering compliance with the study protocol requirements and/or follow-up procedures; those conditions should be discussed with the patient before trial entry.
11. Pregnancy or breastfeeding.
12. History of hypersensitivity to the investigational medicinal product or to any drug with a similar chemical structure or to any excipient present in the pharmaceutical form of the investigational medicinal product.

Restricted medication (relevant for all patients at attribution):

(13) Requirement of anticoagulant/antiplatelet therapy (for example, daily treatment with aspirin >325 mg d^−1^, clopidogrel, warfarin, marcumar, NOAK, systemic LMWH or subcutaneous anticoagulant prophylaxis) unless treatment can be discontinued 7 days (or five half-lives) before initiation of study treatment. Patients may receive heparin flushes for maintenance of indwelling catheters.
(14) Continuous treatment with systemic immunosuppressive medication (including but not limited to prednisone, cyclophosphamide, azathioprine, methotrexate, thalidomide and antitumor necrosis factor (TNF) agents for other diseases than the brain tumor) within 2 weeks before initiation of study treatment. Patients who have received acute, low-dose, systemic immunosuppressant medications (for example, a one-time dose of dexamethasone for nausea) may be enrolled in the study after discussion with and approval by the coordinating investigator (L.K.P.). The use of inhaled corticosteroids and mineralocorticoids (for example, fludrocortisone) for patients with orthostatic hypotension or adrenocortical insufficiency is allowed.

### Subtrial-specific eligibility criteria

Specific diseases (relevant for screening and attribution) are mentioned as follows:

(15) Liver disease is characterized by ‘ALT or AST (≥Grade 2 CTCAE v5.0) confirmed on two consecutive measurements’ OR ‘impaired excretory function (for example, hyperbilirubinemia) or synthetic function’ OR ‘other conditions of decompensated liver disease such as coagulopathy, hepatic encephalopathy, hypoalbuminemia, ascites and bleeding from esophageal varices (≥Grade 2 CTCAE v5.0)’ OR ‘acute viral or active autoimmune, alcoholic or other types of acute hepatitis’.
(16) Known uncorrected coagulopathy, platelet disorder or history of nondrug-induced thrombocytopenia.
(17) Known coronary artery disease, substantial arrhythmias or severe congestive heart failure.
  Immune diseases (relevant for all patients at screening and patients allocated to subtrial D (atezolizumab)) are mentioned as follows:
(18) History of autoimmune disease, including but not limited to myasthenia gravis, myositis, autoimmune hepatitis, systemic lupus erythematosus, rheumatoid arthritis, inflammatory bowel disease, vascular thrombosis associated with antiphospholipid syndrome, Wegener’s granulomatosis, Sjögren’s syndrome, Guillain–Barré syndrome, multiple sclerosis, vasculitis or glomerulonephritis; autoimmune-related hypothyroidism (patients on a stable dose of thyroid replacement hormone are eligible for this study); and type 1 diabetes mellitus (patients on a stable dose of insulin regimen are eligible for this study).
(19) History of idiopathic pulmonary fibrosis, organizing pneumonia (for example, bronchiolitis obliterans), drug-induced pneumonitis, idiopathic pneumonitis or active pneumonitis; history of radiation pneumonitis in the radiation field (fibrosis) is permitted.
(20) Psoriatic arthritis (however, patients with eczema, psoriasis, lichen simplex chronicus or vitiligo with dermatologic manifestations only are permitted provided that they meet the following conditions: rash must cover less than 10% of body surface area (BSA); disease is well controlled at baseline and only requiring low potency topical steroids and no acute exacerbations of underlying condition within the previous 12 months (not requiring psoralen + ultraviolet A radiation, methotrexate, retinoids, biologic agents, oral calcineurin inhibitors, high potency or oral steroids).
(21) Prior allogeneic bone marrow transplantation or solid organ transplant.
(22) Administration of a live, attenuated vaccine within 4 weeks before initiation of study treatment or anticipation that such a live attenuated vaccine will be required during the study.

#### Compound-specific eligibility criteria include

##### Subtrial A: asunercept

No biomarker is defined for this subtrial. Tissue available for molecular diagnosis and for IHC staining with a focus on the CD95/CD95L pathway. Exclusion criteria included (1) hereditary fructose intolerance, (2) prior treatment with APG101 or (3) known coronary artery disease, substantial arrhythmias or severe congestive heart failure.

##### Subtrial B: alectinib

Presence of Alk fusion/point mutation (1–2%). Exclusion criteria included (1) prior therapy with alectinib or another alk inhibitor or known allergy to the compound or any of the ingredients; (2) cotherapy with strong/potent CYP3A inducers and/or inhibitors (for example, ketoconazole, rifampin, rifabutin, phenobarbital, phenytoin, carbamazepine and St. John’s Wort (*Hypericum perforatum*)) within 2 weeks or five half-lives (whichever is longer) before the first dose of study drug treatment and while on treatment with study drug; (3) cotherapy with P-gp-substrates or BCRP-substrates; (4) patients with symptomatic bradycardia; (5) any GI disorder that may affect the absorption of oral medications, such as malabsorption syndrome or status postmajor bowel resection; and (6) liver disease characterized by ALT or AST > 3× ULN (≥5× ULN for patients with concurrent liver metastasis) confirmed on two consecutive measurements or impaired excretory function (for example, hyperbilirubinemia) or synthetic function or other conditions of decompensated liver disease such as coagulopathy, hepatic encephalopathy, hypoalbuminemia, ascites and bleeding from esophageal varices or acute viral or active autoimmune, alcoholic or other types of acute hepatitis.

##### Subtrial C: idasanutlin

Presence of p53 wild-type status and MDM2 amplification (>1.8-fold) or MDM2 overexpression, which will be checked by the molecular tumor board. Exclusion criteria included (1) prior therapy with idasanutlin or known allergy to the compound or any of the ingredients*;* (2) known uncorrected coagulopathy, platelet disorder or history of nondrug-induced thrombocytopenia*;* (3) requirement of anticoagulant/antiplatelet therapy (for example, daily treatment with aspirin >325 mg d^−1^, clopidogrel, warfarin, marcumar, NOAK, systemic LMWH or subcutaneous anticoagulant prophylaxis) unless treatment can be discontinued 7 days (or five half-lives) before initiation of study treatment. Patients may receive heparin flushes for maintenance of indwelling catheters*.* (4) Patients who refuse to potentially receive blood products and/or have a hypersensitivity to blood products*.* (5) Patients unable to temporarily interrupt treatment with moderate to strong CYP2C8 inducers and inhibitors (including gemfibrozil, which is also an inhibitor of UGT1A3), CYP2C8 or OATP1B1/OATP1B3 substrates, or strong CYP3A4 inducers. These agents must be discontinued 7–14 days before the start of study medication.

##### Subtrial atezolizumab

No biomarker is defined for this subtrial. Exclusion criteria included (1) prior therapy with atezolizumab or known allergy to the compound or any of the ingredients; (2) history of autoimmune disease, including but not limited to myasthenia gravis, myositis, autoimmune hepatitis, systemic lupus erythematosus, rheumatoid arthritis, inflammatory bowel disease, vascular thrombosis associated with antiphospholipid syndrome, Wegener’s granulomatosis, Sjögren’s syndrome, Guillain–Barré syndrome, multiple sclerosis, vasculitis or glomerulonephritis, patients with a history of autoimmune-related hypothyroidism on a stable dose of thyroid replacement hormone may be eligible for this study; (3) patients with controlled type 1 diabetes mellitus not on a stable dose of insulin regimen; (4) psoriatic arthritis (however, patients with eczema, psoriasis, lichen simplex chronicus or vitiligo with dermatologic manifestations only are permitted provided that they meet the following conditions—rash must cover less than 10% of BSA, disease is well controlled at baseline and only requiring low potency topical steroids, no acute exacerbations of underlying condition within the previous 12 months (not requiring psoralen + ultraviolet A radiation, methotrexate, retinoids, biologic agents, oral calcineurin inhibitors, high potency or oral steroids)). As well as (5) history of idiopathic pulmonary fibrosis, organizing pneumonia (for example, bronchiolitis obliterans), drug-induced pneumonitis, idiopathic pneumonitis or active pneumonitis; history of radiation pneumonitis in the radiation field (fibrosis) is permitted; (6) active tuberculosis; (7) prior allogeneic bone marrow transplantation or solid organ transplant; (8) administration of a live, attenuated vaccine within 4 weeks before attribution or anticipation that such a live attenuated vaccine will be required during the study; (9) severe infections within the last 4 weeks before attribution includig but not limited to hospitalization for complications of infection, bacteremia or sever pneumonia; (10) signs or symptoms of infection within the last 2 weeks before attribution; (11) received oral or intravenously anibiotics within the last 2 weeks before attribution (patients receiving prophylactic antibiotics (for example, for prevention of a urinary tract infection or chronic obstructive pulmonary disease) are eligible); (12) dexamethasone doses above 4 mg d^−1^ and (13) continous treatment with systemic immunosuppressive medication (including but not limited to prednisone, cyclophosphasmide, azathioprine, methotrexate, thalidomide and TNF agents for other diseases than the brain tumor) within 2 weeks before initiation of study treatment. Patients who have received acute, low-dose, systemic immunosuppressant medications (for example, a one-time dose of dexamethasone for nausea) may be enrolled in the study after discussion with and approval by the coordinating investigator (L.K.P.). The use of inhaled corticosteroids and mineralocorticoids (for example, fludrocortisone) for patients with orthostatic hypotension or adrenocortical insufficiency is allowed.

##### Subtrial E: vismodegib

(1) SHH activation (for example, characterized by mutations in patched homolog 1 (PTCH1) or other downstream pathway mutations) was determined by panel and exome sequencing (for mutation analysis) and expression array and RNA-sequencing for SHH pathway regulation. Deviating from the umbrella protocol—(2) vasectomy was not considered sufficient, and male patients were required to agree to use contraception (condoms with spermicidal jellies or cream) upon study entry, during the study, and for 3 months after the end of the study; (3) female patients were required to agree not to become pregnant or donate lactation during treatment and until 24 months after the stop of treatment and (4) all patients were required to agree not to donate blood during treatment and until 24 months after the stop of treatment. Exclusion criteria included (1) prior therapy with vismodegib or known allergy to the compound or any of the ingredients and (2) cotreatment with a statin or St. John’s Wort.

##### Subtrial F: palbociclib

Presence of CDK4/CDK6 amplification or CDKN2A codeletion. Exclusion criteria included (1) prior therapy with palbociclib or another CDK4/CDK6 inhibitor or known allergy to the compound or any of the ingredients. (2) Cotherapy with strong/potent CYP3A inducers and/or inhibitors (for example, ketoconazole, rifampin, rifabutin, phenobarbital, phenytoin, carbamazepine and St. John’s Wort (*H. perforatum*)) within 2 weeks or five half-lives (whichever is longer) before the first dose of study drug treatment and while on treatment with study drug. (3) Patients with baseline QTc > 470 ms or symptomatic bradycardia. (4) Any GI disorder that may affect the absorption of oral medications, such as malabsorption syndrome or status postmajor bowel resection. (5) Liver disease characterized by ALT or AST > 3× ULN (≥5× ULN for patients with concurrent liver metastasis) confirmed on two consecutive measurements or impaired excretory function (for example, hyperbilirubinemia) or synthetic function or other conditions of decompensated liver disease such as coagulopathy, hepatic encephalopathy, hypoalbuminemia, ascites and bleeding from esophageal varices or acute viral or active autoimmune, alcoholic or other types of acute hepatitis.

##### Subtrial G: temsirolimus

High level of p-mTOR^Ser2448^ as determined by IHC. Exclusion criteria included (1) prior therapy with temsirolimus or known allergy to the compound or any of the ingredients; (2) cotreatment with strong/potent CYP3A4 inducers and/or inhibitors, ACE inhibitors or Ca antagonist.

##### Subtrial TMZ: standard

MGMT status as per umbrella protocol.

### Subtrials, targeted therapies

The warehouse of targeted therapies for the different subtrials consisted of alectinib, idasanutlin, vismodegib, palbociclib and temsirolimus for the match subtrials, as well as asunercept, atezolizumab and TMZ for the nonmatch randomized subtrials. Of note, no prognostic value is so far attributed to the markers used in the N^2^M^2^ trial^2^.

Asunercept (APG101), a CD95-fusion protein, has been shown to be effective and well tolerated in combination with second radiotherapy in progressive glioblastoma^3^. Determination of the safe combination dose of intravenous asunercept was done starting with 600 mg per week with three de-escalation/escalation steps of 200 mg, that is D0 = 400 mg, D1 = 600 mg, D2 = 800 mg in conjunction with radiotherapy.

Alectinib is a second-generation inhibitor of ALK administered orally at 600 mg twice daily. ALK fusions and mutations represent proven biomarkers for alectinib treatment^4^.

The MDM2 inhibitor idasanutlin activates the p53 pathway by blocking the inhibitory MDM2-p53 interaction in *TP53* wild-type tumors. Preclinical studies demonstrated a higher sensitivity towards the drug for *TP53* wild-type tumors with *MDM2* amplification and a primary resistance of tumors harboring *TP53* mutations^11^. Idasanutlin was administered orally on five consecutive days of a 28-day cycle. Optimal dose was determined in the phase 1 part by dose escalation from 100 mg daily until maximum tolerated dose in steps of 50 mg.

Atezolizumab is a monoclonal antibody targeting programmed death-ligand 1. Predictive biomarkers for atezolizumab are currently not defined. Atezolizumab was administered intravenously at 1,200 mg every 3 weeks.

Vismodegib, a small-molecule inhibitor of the SHH signaling pathway, has been approved for the therapy of basal-cell carcinoma in doses of 150 mg daily. Activation of the SHH pathway leads to cell proliferation, upregulation of anti-apoptotic proteins, production of vascular endothelial growth factor and angiopoietins and is considered as a biomarker for a response to vismodegib treatment^15^.

Palbociclib, an oral inhibitor of CDK4 and CDK6, has been approved for the treatment of estrogen receptor-positive, human epidermal growth factor receptor 2-negative breast cancer in combination with aromatase inhibitors or fulvestrant^12^. Activation of CDK4 or CDK6 or CDKN2A/CDKN2B codeletion served as biomarkers for palbociclib treatment. Palbociclib was administered initially at 75 mg with dose escalation steps to 100 and 125 mg during combination with radiotherapy and at 125 mg in adjuvant monotherapy on 21 consecutive days of a 28-day cycle.

Temsirolimus represents an inhibitor of the mechanistic target of rapamycin (mTOR) pathway, which is administered intravenously at 25 mg per week, and was evaluated as a first-line treatment in glioblastoma patients in the EORTC-26082 trial. Phosphorylation of mTOR^serine2448^ (p-mTOR^Ser2448^) was retrospectively found to be predictive for response to temsirolimus^5^. This association is worth prospective confirmation, which is attempted in the present subtrial. As the EORTC-26082 trial showed feasibility and safety of temsirolimus in the exact same patient population and treatment schedule, a formal phase 1 trial was not performed for this subtrial.

TMZ is an alkylating chemotherapy used as SOC for patients with glioblastoma irrespective of *MGMT* status.

### Enrollment

Patients have been enrolled from May 2018 through July 2022 in 13 NOA trial sites in Germany. Based on molecular findings (‘match’/‘no match’), patients have been allocated to seven different subtrials or the control group.

For the ‘match’/‘no match’ decision, fresh tumor tissue and blood from glioblastoma patients with a nonhypermethylated *MGMT* promoter were widely examined by neuropathological analysis. Results were available within a maximum of 3 weeks postoperatively, allowing a dedicated bioinformatics evaluation, which forms the basis for the final treatment decision by the MTB and afterwards a timely initiation (≤6 weeks) of postoperative treatments. The workflow and timelines of molecular diagnostics and treatment decisions have been summarized in the published study protocol^18^.

### Molecular diagnostics (Discovery)

Molecular analysis consisted of an epigenome-wide array, panel sequencing, whole exome, low-coverage whole genome and transcriptome sequencing as well as expression array detecting somatic single-nucleotide variants (SNV), small inserts/deletions, copy number variants, focal amplifications or overexpression of affected genes and pathways. At least panel sequencing, methylation array and phospho-mTOR IHC have been required at the MTB. Results of the molecular profiling have been discussed in the MTB on a weekly basis and treatment recommendations were identified. The MTB consisted of W.W., A.W., F.S., T.K. and representatives from the sites of the discussed patient.

For cases with detection of several targetable mutations, a previously described ranking algorithm was applied^30^ by the molecular tumor board.

Sequence and methylation data have been deposited at the European Genome-phenome Archive, which is hosted by the European Bioinformatics Institute and the Centre for Genomic Regulation under accession EGAS00001008033 (https://ega-archive.org; RRID: SCR_004944).

### Molecular assessments and molecular tumor board

Molecular analysis consisted of an epigenome-wide array, panel sequencing, whole exome, low-coverage whole genome and transcriptome sequencing as well as expression array detecting somatic SNV, small inserts/deletions, copy number variants, focal amplifications or overexpression of affected genes and pathways.

#### *MGMT* pyrosequencing

Analysis of *MGMT* promoter methylation status through pyrosequencing was performed with the Therascreen MGMT Pyro Kit (Qiagen) according to the manufacturer’s instructions. Quantitative measurement of methylation in four cytosine-phosphate-guanine (CpG) sites in exon 1 of the *MGMT* gene was performed. The cutoff was set at 8%.

#### Phospho-mTOR IHC

IHC to detect phospho-mTOR was performed as described previously^5^ using a heat antigen retrieval procedure (citrate buffer) and the phospho-mTOR antibody (Ser-2448; 2976, Cell Signaling Technology) in a dilution of 1:100 according to the manufacturer’s recommendations.

#### DNA methylation profiling

The Illumina Infinium HumanMethylationEPIC (EPIC) bead chip kit was used to obtain the DNA methylation status at >850,000 CpG sites (Illumina) according to the manufacturer’s instructions at the Genomics and Proteomics Core Facility of the German Cancer Research Center, from paraffin-embedded tissue.

MGMT promoter methylation was assessed with the use of Illumina EPIC methylation arrays based on the MGMT-STP27 model^9^. Classification of tumors was performed with the Heidelberg classifier (www.molecularneuropathology.org (ref. ^31^)).

Samples were analyzed using the R (www.r-project.org) based methylation pipeline ‘ChAMP’ (version 2.34.0, RRID:SCR_012891). Briefly, filtering was done for multihit sites, SNPs and XY chromosome-related CpGs; then, data were normalized with a BMIQ-based method.

Custom scripts based on the R packages ‘minfi’ (version 1.26.2) and ‘conumee’ (version 1.14.0) were implemented for CNV profiling and visualization.

#### DNA panel sequencing

DNA sequencing was conducted as described previously^32^. Briefly, an adapted version of the original panel consisting of a set of 170+ genes recurrently altered in brain tumors was used from paraffin-embedded tissue samples.

DNA was extracted on the Promega Maxwell device (Promega) following the manufacturer’s instructions. Sequencing was performed on a NovaSeq instrument (Illumina, RRID:SCR_016387).

For data processing, raw data were demultiplexed and converted into fastq format with subsequent alignment to the reference genome. For SNV calling, we used SAMtools mpileup (version 1.17, RRID:SCR_002105), and for InDel calling Platypus^33^ was used. Common seq artifacts were removed. Filtering was done for snp138 variants and exonic SNVs were included.

#### Molecular tumor board decision in case of multiple targetable alterations

For cases with detection of several targetable mutations, a previously described ranking algorithm has been used^30^. If more than one mutation obtains the highest rank, the match was randomly allocated to specific subtrials or assigned for the best-performing subtrial, if already known.

### Treatment

Based on the decision of the MTB, patients were enrolled in five different subtrials (‘match’) or randomized between asunercept, atezolizumab and SOC (‘no match’; Fig. 1a). Radiotherapy built the backbone for each subtrial at 60 Gy in 2 Gy fractions in working-daily radiotherapy sessions over a period of 6 weeks. Experimental treatments start with the initiation of radiotherapy at maximum tolerated dose, which is predefined or determined in phase 1 parts of the subtrials, and continued until progression, undue toxicity, death or patient’s decision, whichever comes first. As a control, intervention patients without any of the defined molecular alterations and randomized to SOC received concomitant TMZ chemotherapy (75 mg m^−^^2^ BSA) plus radiotherapy followed by six cycles of TMZ maintenance therapy (150/200 mg m^−^^2^ body surface) according to the SOC. Safety endpoints of phase 1 parts have been determined until the end of combined modality treatment and efficacy data are collected until the EOS or death, whichever comes first.

### Withdrawal of patients

Patients were withdrawn from the trial at any time at their own request in case of serious AEs caused by the investigational medicinal product, except for manageable abnormal laboratory values or other general safety issues by the investigator. All ongoing AEs and SAEs of withdrawn patients have been followed up until stabilization or resolution.

### Assessments of safety and efficacy

#### Assessment of efficacy

For the primary efficacy endpoint, PFS-6—defined as the proportion of patients achieving PFS-6 after treatment initiation—was determined and is presented in summary tables, along with Pearson–Clopper 95% CIs. Radiographic progression was evaluated according to RANO^34^ or iRANO for atezolizumab^35^ by the central neuroradiology and clinical progression by deterioration of Karnofsky performance status. Notably, the protocol contained detailed instructions to avoid too early cessation of study drug in case of presumed pseudoprogression and mandates a confirmatory scan whenever clinically possible.

For secondary efficacy endpoints PFS and OS, defined as the time from treatment start until progression or death, were determined and analyzed using the Kaplan–Meier method for survival curves and Greenwood’s formula for estimating the standard error of event rates. Patients without an event relevant for PFS or OS at the time of analysis are censored at the last disease assessment showing no progression or at baseline if the patient has no postbaseline disease assessments (PFS) or last date they were known to be alive (OS). Please note that patients with a death event in the survival follow-up (without a preceding progression event) are only considered as having an event relevant for PFS if regular information on disease assessment for that patient is available. Otherwise, the patient is censored at the last date of disease assessment. Given the low number of patients in each subtrial and the multiplicity of the analyses, all statistical tests are strictly exploratory.

The primary efficacy endpoint, PFS-6, according to RANO criteria as a binary endpoint, is analyzed with a one-sample one-sided binomial test of the null hypothesis (*H*_0_—*P* = 0.231).

No formal statistical comparisons between the subtrials are planned. However, results obtained for the control group and different subtrials may be used for considerations of changes regarding efficacy or recommendations for further phase 2 and phase 3 trials.

### Analysis of the (secondary) safety endpoints

For the secondary safety endpoint (RLT), the frequency and types of RLTs are tabulated. Summary tables (Supplementary Information) present the number and percentage of patients experiencing RLTs, accompanied by exact two-sided 95% Clopper–Pearson confidence intervals. All patients who received the final dose are included in the analysis.

AEs are analyzed. Frequencies of patients experiencing at least one AE are displayed. Detailed information collected for each AE includes a description of the event, duration, whether the AE was serious, intensity, relationship to study drug, action taken and clinical outcome. Summaries of incidence rates (frequencies and percentages) of AEs by MedDRA (version 23.0) System Organ Class and Preferred Term (PT) are prepared. Such summaries are displayed for all AEs, AEs by intensity and AEs by relationship to study drug. Summary tables present the number of patients observed with AEs and the corresponding percentages.

Karnofsky index is summarized descriptively for each visit by presenting the absolute and relative frequencies (percentages).

Vital signs (blood pressure, heart rate, temperature, body weight, body height (only at screening), respiratory rate) are summarized descriptively by visit. The number of observations (*n*, *n*_miss_), mean, s.d., median, minimum and maximum are presented. This includes changes (differences) from the baseline assessment, except for body height.

Clinical laboratory parameters (hematology, chemistry, urinalysis) and electrocardiogram (ECG) are summarized descriptively by visit. Number of observations (*n*, *n*_miss_), mean, s.d., median, minimum and maximum are presented (for ECG: only for abnormal results). The number of patients with laboratory values that are below, within or above normal ranges is tabulated for each parameter. Descriptive summaries (mean, s.d., median, minimum and maximum) of actual values and of changes from baseline are presented for each parameter. The number and percentage of patients with normal and abnormal ECG results at baseline and follow-up are tabulated. ECG findings are tabulated by patient.

All AEs that occurred during the trial after the first experimental treatment have been recorded, graded according to the CTCAE Version 5.0 at every study visit, and followed up until resolution or stabilization. Safety endpoints were assessed by the frequency of AEs and the number of laboratory values that fall outside of predetermined ranges. AEs were described by event, duration, seriousness, intensity and relationship to the investigational medicinal product, actions taken and clinical outcome and reported as tables of frequencies at PT and Medical Dictionary for Regulatory Activities (MedDRA) SOC.

#### Phase 1

The primary safety endpoint was the determination of posterior probability of DLT, defined as all AEs coded using MedDRA ≥Grade 3 according to the National Cancer Institute CTCAE v5.0 that are definitely, probably or possibly related to the administration of the investigational medical product in combination with radiotherapy.

The secondary safety endpoint was RLT, defined as any toxicity that meets the criteria of a DLT, but is observed after the end of the combination therapy in phase 1 or during phase 2a of the trial for patients recruited for phase 1.

The secondary efficacy endpoint was PFS-6 according to RANO criteria as a binary endpoint. See also the primary efficacy endpoint for phase 2a for more information.

#### Phase 2a

The primary efficacy endpoint was the PFS-6 according to RANO criteria as a binary endpoint. Response was defined as the proportion of patients without progression at 6 months after study entry. The basis for the baseline assessment of the disease progression was an initial MRI ≤ 2 weeks before the start of therapy (for radiotherapy planning).

Secondary efficacy endpoints were PFS and OS. PFS is defined as time from study entry (day of attribution = baseline) until the day of first documentation of clinical or radiographic tumor progression or death of any cause (whichever occurs first). Patients without an event relevant for PFS (progression or death) at the time of analysis were censored at the last disease assessment showing no progression or at baseline if the patient had no postbaseline disease assessments. OS was defined as the time from study entry (day of attribution) until death due to any cause. Patients still alive or lost to follow-up at the time of the analysis were censored at the last date they were known to be alive.

The secondary safety endpoint was RLT, defined as any toxicity that meets the criteria of a DLT, but is observed after the end of the combination therapy in phase 1 (for patients recruited at the final dose of phase 1) or during phase 2a of the trial.

### Interim analysis and stopping rules

Two interim analyses per subtrial were carried out once the PFS-6 endpoint had been determined for 15 and 25 patients, respectively. Tests for futility based on predictive power and for decisions regarding acceptance of the DLT rate of experimental treatment for phase 2a trial were performed. For that, the posterior distribution of DLT rate was calculated with a β-binomial model with a noninformative prior and a Bayesian criterion was used for continuous monitoring of toxicity. Recruitment was planned to be suspended if the predictive power is lower than 10% or if the a posteriori probability that the true toxicity rate (at the given dose level of dose escalation in phase 1 part of indicated subtrials) is 30% or higher exceeds 95%. In both scenarios, the Data and Safety Monitoring Committee advised the coordinating investigator if patient accrual should be stopped.

### Sample size estimation

In the phase 1 parts, patients were enrolled depending on observed toxicities. In the phase 2a parts, a maximum of 40 patients were to be accrued for evaluation in each subtrial, with nine patients from the appropriate dose of an eventual phase 1 part included. The exact number depends on early stopping for toxicity or futility or overshooting in cases of rapid enrollment. The ‘nonmatching’ group was anticipated to include approximately 35% of all screened study patients. Therefore, 12% of all screened patients were expected to be enrolled in the control group receiving TMZ.

### Data collection

Clincase version 2.7 (EDC System) has been used to allow on-site data entry. Data analysis was done on exports from this system using SAS version 9.4.

### Statistical analysis

Data for time-to-event endpoints were further collected after the EOS in the survival follow-up. Survival follow-up information was collected until the overall EOS of the umbrella trial.

#### Phase 1

For the primary safety endpoint (DLTs), the different examined dose levels are presented, together with the amount and type (PT and SOC level) of DLTs, patients experienced at these dose levels. Summary tables present the number of patients observed with a DLT and the corresponding percentage. Exact 95% two-sided Clopper–Pearson CIs are presented (for more details, see Supplementary Information).

The number of responses, defined as patients being free of progression after 6 months (confirmed by MRI), is presented in descriptive tables together with corresponding percentages, and exact 95% two-sided Clopper–Pearson CIs. Patients with missing information on PFS-6 are tabulated as missing.

#### Phase 2a

The primary efficacy endpoint, PFS-6, according to RANO criteria, is analyzed as a binary endpoint with a one-sample one-sided binomial test of the null hypothesis (*H*_0_—*P* = 0.231). The number of responses, defined as patients being definitely free of progression after 6 months (confirmed by MRI scans), is presented in descriptive tables together with corresponding percentages and exact 95% two-sided Clopper–Pearson CIs. Patients with missing information on PFS-6 are tabulated as missing, but for the calculation of the *P* value, those patients are assumed to be nonresponders. The *α*-level for the primary analysis is 10%.

Response assessment was determined by combining information from the clinical trial site (local RANO assessment, a status page in the eCRF showing the information if the patient experienced progression and/or received other antitumor therapy during the 6 months after study entry and survival follow-up in case of premature EOS) and central RANO assessment performed by central neuroradiology in Heidelberg. If the clinical trial site stated a progression on the status page, the patient was assumed to be a nonresponder, irrespective of other information. Central RANO assessment was the preferred type of assessment, but if not available (or differing from a stated progression on the status page), other sources of information have been used to determine the response status/assessment.

### Reporting summary

Further information on research design is available in the Nature Portfolio Reporting Summary linked to this article.

## Online content

Any methods, additional references, Nature Portfolio reporting summaries, source data, extended data, supplementary information, acknowledgements, peer review information; details of author contributions and competing interests; and statements of data and code availability are available at 10.1038/s41591-025-03928-9.

## Supplementary information

Supplementary Information Supplementary Tables 1–4 and Note (protocol: EudraCT No: 2015-002752-27 final report).

Reporting Summary

## Data Availability

Sequence and methylation data have been deposited at the European Genome-phenome Archive (EGA), which is hosted by the European Bioinformatics Institute and the Centre for Genomic Regulation under accession EGAS00001008033 (https://ega-archive.org; RRID: SCR_004944). Patient outcomes and raw molecular data are available upon request to the corresponding author (W.W.) within 4 weeks from request, as long as they are in line with the ethics approvals.
