# Supplementary material for: Molecularly matched targeted therapies plus radiotherapy in glioblastoma: the phase 1/2a N2M2 umbrella trial
Source: Nat Med. 2025 Sep 5;31(10):3534–41. doi: 10.1038/s41591-025-03928-9 (PMC12532562; doi:10.1038/s41591-025-03928-9)
Supplement: Supplementary file 1 — Supplementary Tables 1–4 and Note (protocol: EudraCT No: 2015-002752-27 final report). [file 41591_2025_3928_MOESM1_ESM.pdf]

nature medicine

Supplementary information https://doi.org/10.1038/s41591-025-03928-9

Molecularly matched targeted therapies plus radiotherapy in glioblastoma: the phase 1/2a N^2M^2 umbrella trial

In the format provided by the authors and unedited

Supplement

Supplementary Data Table 1: Patient characteristics (screening population)

	All Patients	Male	Female
**Sex**, n (%)			
Male	192 (63.8)	192 (100)	0
Female	109 (36.2)	0	109 (100)
**Age continuous (years)**, mean (SD)	58.9 (9.71)	59.0 (9.55)	58.8 (10.03)
**BMI (kg/m^2)**, mean (SD)*	26.6 (4.64)	27.0 (4.08)	25.7 (5.41)
**Height (cm)**, mean (SD)*	174.0 (9.07)	178.6 (6.91)	166.0 (6.50)
**Weight (kg)**, mean (SD)*	80.7 (16.95)	86.3 (14.85)	71.0 (16.02)
**Age categorical (years)**, n (%)			
18-44	21 (7.0)	12 (6.3)	9 (8.3)
45-64	193 (64.1)	128 (66.7)	65 (59.6)
≥65	87 (28.9)	52 (27.1)	35 (32.1)
**Ethnic group**, n (%)			
Caucasian/white	296 (98.3)	188 (97.9)	108 (99.1)
Oriental	5 (1.7)	4 (2.1)	1 (0.9)
**Karnofsky performance status (KPS) at baseline**, n(%)			
60	1 (0.8)	1 (0.5)	0
70	15 (5.0)	7 (3.6)	8 (7.3)
80	64 (21.2)	43 (22.4)	21 (19.3)
90	113 (37.6)	66 (34.3)	47 (43.1)
100	56 (18.6)	41 (21.4)	15 (13.8)
Missing	52 (17.3)	34 (17.7)	18 (16.5)
**Resection status****, n (%)			
Biopsy	17 (5.6)	8 (4.2)	9 (8.3)
Partial resection	105 (34.9)	62 (32.3)	43 (39.4)
Complete resection	186 (61.8)	123 (64.1)	63 (57.8)
Missing resection	7 (2.33)	7 (3.7)	3 (2.8)
**Glioblastoma methylation classes**, n (%)			
Mesenchymal	82 (27.2)	52 (27.1)	30 (27.5)
RTK I	67 (22.3)	50 (26.0)	17 (15.6)
RTK II	101 (33.6)	60 (31.3)	41 (37.6)
Other	27 (9.0)	18 (9.4)	9 (8.3)
Missing	24 (8.0)	12 (6.3)	12 (11.0)

*For overall 10 patients neither weight nor height were documented
** Some patients had both (partial or complete) resection and biopsy or only resection. Three male patients only had biopsy.

Supplementary Data Table 2: Safety results of the phase I population for all dose groups

**Related AE**	**Arm A: asunercept (N=9)**	**Arm C: idasanutlin (N=9)**	**Arm D: atezolizumab (N=9)**	**Arm F: palbociclib (N=13)**
Any AE	1 (11.1)	9 (100)	8 (88.9)	12 (92.3)
Any SAE	0	4 (44.4)	3 (33.3)	1 (7.7)
Any Severe Adverse Event (grade 3 or 4)	0	5 (55.6)	2 (22.2)	6 (46.2)
Any DLT	0	2 (22.2)	2 (22.2)	4 (30.8)
Any RLT	0	4 (44.4)	0	5 (38.5)
Discontinued study drug due to AE	0	4 (44.4)	3 (33.3)	3 (23.1)
Dose reduction or temporary discontinuation due to AE	0	5 (55.6)	1 (11.1)	6 (46.2)
AE resulting in death	0	0	0	0

Supplementary Data Table 3: Patient characteristics per subtrial – Full Analysis Set (FAS)

	**Subtrial A: N(%)**	**Subtrial D: N(%)**	**Subtrial F: N(%)**	**Subtrial G: N(%)**	**SOC: N(%)**
**Sex**, n (%)					
Male	19 (73.1)	28 (66.7)	26 (63.4)	27 (58.7)	36 (66.7)
Female	7 (26.9)	14 (33.3)	15 (36.6)	19 (41.3)	18 (33.3)
**Age continuous (years)**, mean (SD)	59.0 (7.94)	58.2 (10.17)	58.0 (9.63)	59.4 (10.15)	58.6 (7.79)
**BMI (kg/m^2)**, mean (SD)*	25.8 (3.49)	26.8 (5.08)	25.8 (4.73)	26.7 (4.15)	27.0 (4.83)
**Height (cm)**, mean (SD)*	175.7 (6.82)	173.9 (8.76)	172.9 (9.12)	173.5 (9.18)	176.1 (9.82)
**Weight (kg)**, mean (SD)*	79.9 (12.41)	81.3 (17.67)	77.7 (18.30)	80.6 (15.09)	84.2 (19.20)
**Age categorical (years)**, n (%)					
18-44	2 (7.7)	3 (7.1)	4 (9.8)	4 (8.7)	1 (1.9)
45-64	17 (65.4)	25 (59.5)	27 (65.9)	25 (54.3)	40 (74.1)
≥65	7 (26.9)	14 (33.3)	10 (24.4)	17 (37.0)	13 (24.1)
**Ethnic group**, n (%)					
Caucasian/white	26 (100)	42 (100)	41 (100)	45 (97.8)	52 (96.3)
Oriental	0	0	0	1 (2.2)	2 (3.7)
**Karnofsky performance status (KPS) at baseline**, n(%)					
70	0	1 (2.4)	3 (7.3)	3 (6.5)	6 (11.1)
80	8 (30.8)	10 (23.8)	8 (19.5)	13 (28.3)	15 (27.8)
90	11 (42.3)	21 (50.0)	19 (46.3)	24 (52.2)	21 (38.9)
100	7 (26.9)	10 (23.8)	11 (26.8)	6 (13.0)	12 (22.2)
**Resection status**, n (%)**					
Biopsy	2 (7.7)	1 (2.4)	3 (7.3)	1 (2.2)	2 (3.7)
Partial resection	11 (42.3)	15 (35.7)	17 (41.5)	15 (32.6)	18 (33.3)
Complete resection	14 (53.8)	27 (64.3)	24 (58.5)	31 (67.4)	36 (66.7)
**MGMT promotor methylation**, median (range)	0.02 (0.01-0.07)	0.02 (0.01-0.07)	0.02 (0.01-0.08)	0.01 (0.01-0.06)	0.02 (0.01-0.07)
**Glioblastoma methylation classes**, n (%)					
Mesenchymal	9 (34.6)	12 (28.6)	8 (19.5)	17 (37.0)	15 (27.8)
RTK I	5 (19.2)	6 (14.3)	16 (39.0)	10 (21.7)	15 (27.8)
RTK II	7 (26.9)	18 (42.9)	17 (41.5)	16 (34.8)	19 (35.2)
Other	5 (19.2)	6 (14.3)	0	3 (6.5)	5 (9.3)
**p-mTOR score**, mean (SD)	146.5 (39.7)	135.9 (49.1)	115.2 (46.2)	182.8 (18.7)	139.0 (39.2)

*For overall 2 patients neither weight nor height were documented

**Some patients had both (partial or complete) resection and biopsy or only resection. One (male) patient had only biopsy

Supplementary Data Table 4: Comparison of FAS and EES datasets

	FAS		EES	
	n	PFS-6	n [% of FAS]	PFS-6
**Arm A (asunercept)**	26	15.4%	26 (100%)	15.4%
**Arm D (atezolizumab)**	42	21.4%	40 (95%)	22.5%
**Arm F (palbociclib)**	41	24.4%	40 (98%)	25.0%
**Arm G (temsirolimus)**	46	39.1%	46 (100%)	39.1%
**SOC (temozolomide)**	54	18.5%	54 (100%)	18.5%

Incidence and Severity of Adverse Events (All Causalities) - Full Analysis Set

AEs that occured on or after the date of first medication and before end of observation have been considered as treament emergent AEs.

		N=26						
			Severity					
System organ class (MedDRA 23.0)	Preferred Term (MedDRA 23.0)	Number of patients (%) *	Missing	Grade 1	Grade 2	Grade 3	Grade 4	Grade 5
Blood and lymphatic system disorders	Patients with one or more Events	4 (15.4)	0	1	2	1	0	0
	Leukocytosis	1 (3.8)	0	0	1	0	0	0
	Lymphopenia	3 (11.5)	0	1	1	1	0	0
	Monocytosis	1 (3.8)	0	1	0	0	0	0
	Neutrophilia	1 (3.8)	0	0	1	0	0	0
Cardiac disorders	Patients with one or more Events	1 (3.8)	0	0	1	0	0	0
	Arrhythmia	1 (3.8)	0	0	1	0	0	0
Ear and labyrinth disorders	Patients with one or more Events	3 (11.5)	0	3	0	0	0	0
	Hypoacusis	1 (3.8)	0	1	0	0	0	0
	Vertigo	2 (7.7)	0	2	0	0	0	0
Eye disorders	Patients with one or more Events	5 (19.2)	0	3	1	1	0	0
	Blepharitis	1 (3.8)	0	0	1	0	0	0
	Eyelid oedema	2 (7.7)	0	2	0	0	0	0
	Periorbital oedema	1 (3.8)	0	1	0	0	0	0
	Retinal detachment	1 (3.8)	0	0	0	1	0	0
	Visual impairment	1 (3.8)	0	1	0	0	0	0
Gastrointestinal disorders	Patients with one or more Events	9 (34.6)	0	8	1	0	0	0
	Abdominal pain upper	1 (3.8)	0	1	0	0	0	0
	Constipation	2 (7.7)	0	2	0	0	0	0
	Faeces discoloured	1 (3.8)	0	1	0	0	0	0
	Nausea	4 (15.4)	0	3	1	0	0	0
	Stomatitis	1 (3.8)	0	1	0	0	0	0
	Vomiting	1 (3.8)	0	1	0	0	0	0

The AE with the highest grading for each preferred term for each patient was taken. A patient who reports two or more Different preferred terms which are in the same system organ class, is counted only once in the system organ class total.
The highest grading of this patient in the respective system organ class will be taken.

Section 13 Table 6.1.2.1.1
Incidence and Severity of Adverse Events (All Causalities) - Full Analysis Set
AEs that occured on or after the date of first medication and before end of observation have been considered as treament emergent AEs.

		N=26						
			Severity					
System organ class (MedDRA 23.0)	Preferred Term (MedDRA 23.0)	Number of patients (%) *	Missing	Grade 1	Grade 2	Grade 3	Grade 4	Grade 5
General disorders and administration site conditions	Patients with one or more Events	13 (50.0)	0	8	4	1	0	0
	Chills	3 (11.5)	0	3	0	0	0	0
	Cyst	1 (3.8)	0	0	0	1	0	0
	Fatigue	9 (34.6)	0	6	3	0	0	0
	Gait disturbance	1 (3.8)	0	0	1	0	0	0
	Impaired healing	1 (3.8)	0	1	0	0	0	0
	Influenza like illness	1 (3.8)	0	1	0	0	0	0
	Oedema	1 (3.8)	0	0	1	0	0	0
	Oedema peripheral	1 (3.8)	0	1	0	0	0	0
Immune system disorders	Patients with one or more Events	1 (3.8)	0	0	1	0	0	0
	Drug hypersensitivity	1 (3.8)	0	0	1	0	0	0
Infections and infestations	Patients with one or more Events	7 (26.9)	0	3	1	3	0	0
	COVID-19	1 (3.8)	0	0	0	1	0	0
	Conjunctivitis	1 (3.8)	0	1	0	0	0	0
	Infected bite	1 (3.8)	0	0	0	1	0	0
	Infection	1 (3.8)	0	0	0	1	0	0
	Influenza	1 (3.8)	0	1	0	0	0	0
	Nasopharyngitis	1 (3.8)	0	0	1	0	0	0
	Respiratory tract infection	1 (3.8)	0	1	0	0	0	0
	Urinary tract infection	1 (3.8)	0	0	1	0	0	0
	Urinary tract infection bacterial	1 (3.8)	0	1	0	0	0	0
Injury, poisoning and procedural complications	Patients with one or more Events	4 (15.4)	0	4	0	0	0	0
	Head injury	1 (3.8)	0	1	0	0	0	0
	Lip injury	1 (3.8)	0	1	0	0	0	0
	Muscle strain	1 (3.8)	0	1	0	0	0	0
	Radiation skin injury	1 (3.8)	0	1	0	0	0	0

The AE with the highest grading for each preferred term for each patient was taken. A patient who reports two or more Different preferred terms which are in the same system organ class, is counted only once in the system organ class total.
The highest grading of this patient in the respective system organ class will be taken.

Section 13 Table 6.1.2.1.1

Incidence and Severity of Adverse Events (All Causalities) - Full Analysis Set

AEs that occured on or after the date of first medication and before end of observation have been considered as treament emergent AEs.

		N=26						
			Severity					
System organ class (MedDRA 23.0)	Preferred Term (MedDRA 23.0)	Number of patients (%) *	Missing	Grade 1	Grade 2	Grade 3	Grade 4	Grade 5
Investigations	Patients with one or more Events	7 (26.9)	0	5	1	1	0	0
	Alanine aminotransferase increased	1 (3.8)	0	0	0	1	0	0
	Blood creatine phosphokinase increased	1 (3.8)	0	1	0	0	0	0
	C-reactive protein increased	1 (3.8)	0	1	0	0	0	0
	Gamma-glutamyltransferase increased	2 (7.7)	0	1	0	1	0	0
	Lymphocyte count decreased	1 (3.8)	0	0	1	0	0	0
	Weight decreased	2 (7.7)	0	2	0	0	0	0
	Weight increased	1 (3.8)	0	1	0	0	0	0
	White blood cell count increased	1 (3.8)	0	1	0	0	0	0
Metabolism and nutrition disorders	Patients with one or more Events	5 (19.2)	0	2	3	0	0	0
	Decreased appetite	2 (7.7)	0	0	2	0	0	0
	Hypoglycaemia	1 (3.8)	0	0	1	0	0	0
	Hypokalaemia	1 (3.8)	0	1	0	0	0	0
	Hyponatraemia	1 (3.8)	0	1	0	0	0	0
	Type 2 diabetes mellitus	1 (3.8)	0	1	0	0	0	0
Musculoskeletal and connective tissue disorders	Patients with one or more Events	1 (3.8)	0	1	0	0	0	0
	Fistula	1 (3.8)	0	1	0	0	0	0
Nervous system disorders	Patients with one or more Events	18 (69.2)	0	5	9	4	0	0
	Aphasia	4 (15.4)	0	1	3	0	0	0
	Apraxia	2 (7.7)	0	1	1	0	0	0
	Ataxia	2 (7.7)	0	0	2	0	0	0
	Cognitive disorder	2 (7.7)	0	1	1	0	0	0
	Coordination abnormal	1 (3.8)	0	1	0	0	0	0
	Disturbance in attention	4 (15.4)	0	0	4	0	0	0
	Dyscalculia	1 (3.8)	0	0	1	0	0	0
	Dysgeusia	1 (3.8)	0	0	1	0	0	0

The AE with the highest grading for each preferred term for each patient was taken. A patient who reports two or more Different preferred terms which are in the same system organ class, is counted only once in the system organ class total.
The highest grading of this patient in the respective system organ class will be taken.

Section 13 Table 6.1.2.1.1

Incidence and Severity of Adverse Events (All Causalities) - Full Analysis Set

AEs that occured on or after the date of first medication and before end of observation have been considered as treament emergent AEs.

		N=26						
			Severity					
System organ class (MedDRA 23.0)	Preferred Term (MedDRA 23.0)	Number of patients (%) *	Missing	Grade 1	Grade 2	Grade 3	Grade 4	Grade 5
Nervous system disorders	Epilepsy	1 (3.8)	0	0	0	1	0	0
	Headache	8 (30.8)	0	6	1	1	0	0
	Hemianopia	2 (7.7)	0	1	1	0	0	0
	Hemiparesis	2 (7.7)	0	1	1	0	0	0
	Hypoaesthesia	1 (3.8)	0	1	0	0	0	0
	Hyposmia	1 (3.8)	0	0	1	0	0	0
	Memory impairment	2 (7.7)	0	0	2	0	0	0
	Muscle spasticity	1 (3.8)	0	0	1	0	0	0
	Myoclonic epilepsy	1 (3.8)	0	0	0	1	0	0
	Neurological decompensation	1 (3.8)	0	0	0	1	0	0
	Seizure	5 (19.2)	0	2	3	0	0	0
	Syncope	1 (3.8)	0	1	0	0	0	0
	Taste disorder	1 (3.8)	0	0	1	0	0	0
Psychiatric disorders	Patients with one or more Events	3 (11.5)	0	0	3	0	0	0
	Confusional state	1 (3.8)	0	0	1	0	0	0
	Depressed mood	1 (3.8)	0	0	1	0	0	0
	Disorientation	1 (3.8)	0	0	1	0	0	0
Renal and urinary disorders	Patients with one or more Events	1 (3.8)	0	0	1	0	0	0
	Incontinence	1 (3.8)	0	0	1	0	0	0
Respiratory, thoracic and mediastinal disorders	Patients with one or more Events	1 (3.8)	0	1	0	0	0	0
	Cough	1 (3.8)	0	1	0	0	0	0
Skin and subcutaneous tissue disorders	Patients with one or more Events	8 (30.8)	0	5	3	0	0	0
	Alopecia	6 (23.1)	0	5	1	0	0	0
	Erythema	1 (3.8)	0	1	0	0	0	0
	Pityriasis rosea	1 (3.8)	0	0	1	0	0	0
	Skin disorder	1 (3.8)	0	1	0	0	0	0
	Skin ulcer	1 (3.8)	0	0	1	0	0	0

The AE with the highest grading for each preferred term for each patient was taken. A patient who reports two or more Different preferred terms which are in the same system organ class, is counted only once in the system organ class total.
The highest grading of this patient in the respective system organ class will be taken.

Incidence and Severity of Adverse Events (All Causalities) - Full Analysis Set

AEs that occured on or after the date of first medication and before end of observation have been considered as treament emergent AEs.

		N=26						
			Severity					
System organ class (MedDRA 23.0)	Preferred Term (MedDRA 23.0)	Number of patients (%) *	Missing	Grade 1	Grade 2	Grade 3	Grade 4	Grade 5
Vascular disorders	Patients with one or more Events	4 (15.4)	0	2	1	1	0	0
	Deep vein thrombosis	1 (3.8)	0	0	0	1	0	0
	Hypertension	1 (3.8)	0	1	0	0	0	0
	Hypotension	1 (3.8)	0	0	1	0	0	0
	Lymphoedema	1 (3.8)	0	1	0	0	0	0

The AE with the highest grading for each preferred term for each patient was taken. A patient who reports two or more Different preferred terms which are in the same system organ class, is counted only once in the system organ class total.

The highest grading of this patient in the respective system organ class will be taken.

Section 13 Table 6.1.2.2.1
Incidence and Severity of Adverse Events (All Causalities) - Safety Population Phase 1-Dosegroup: All Dosegroups

AEs that occured on or after the date of first medication and before end of observation have been considered as treament emergent AEs.

		N=9						
			Severity					
System organ class (MedDRA 23.0)	Preferred Term (MedDRA 23.0)	Number of patients (%) *	Missing	Grade 1	Grade 2	Grade 3	Grade 4	Grade 5
Ear and labyrinth disorders	Patients with one or more Events	2 (22.2)	0	2	0	0	0	0
	Vertigo	2 (22.2)	0	2	0	0	0	0
Gastrointestinal disorders	Patients with one or more Events	4 (44.4)	0	3	1	0	0	0
	Abdominal pain upper	1 (11.1)	0	1	0	0	0	0
	Constipation	1 (11.1)	0	1	0	0	0	0
	Dry mouth	1 (11.1)	0	0	1	0	0	0
	Nausea	2 (22.2)	0	1	1	0	0	0
	Stomatitis	1 (11.1)	0	1	0	0	0	0
General disorders and administration site conditions	Patients with one or more Events	5 (55.6)	0	1	3	1	0	0
	Cyst	1 (11.1)	0	0	0	1	0	0
	Fatigue	3 (33.3)	0	1	2	0	0	0
	Gait disturbance	2 (22.2)	0	0	2	0	0	0
	General physical health deterioration	1 (11.1)	0	0	1	0	0	0
	Impaired healing	1 (11.1)	0	1	0	0	0	0
	Influenza like illness	1 (11.1)	0	1	0	0	0	0
	Oedema	2 (22.2)	0	1	1	0	0	0
Infections and infestations	Patients with one or more Events	4 (44.4)	0	3	0	1	0	0
	Infected bite	1 (11.1)	0	0	0	1	0	0
	Influenza	1 (11.1)	0	1	0	0	0	0
	Otitis externa	1 (11.1)	0	1	0	0	0	0
	Postoperative wound infection	1 (11.1)	0	1	0	0	0	0
	Urinary tract infection bacterial	1 (11.1)	0	1	0	0	0	0

The AE with the highest grading for each preferred term for each patient was taken. A patient who reports two or more Different preferred terms which are in the same system organ class, is counted only once in the system organ class total.
The highest grading of this patient in the respective system organ class will be taken.

Section 13 Table 6.1.2.2.1
Incidence and Severity of Adverse Events (All Causalities) - Safety Population Phase 1-Dosegroup: All Dosegroups

AEs that occured on or after the date of first medication and before end of observation have been considered as treament emergent AEs.

		N=9						
			Severity					
System organ class (MedDRA 23.0)	Preferred Term (MedDRA 23.0)	Number of patients (%) *	Missing	Grade 1	Grade 2	Grade 3	Grade 4	Grade 5
Investigations	Patients with one or more Events	5 (55.6)	0	3	1	1	0	0
	Alanine aminotransferase increased	2 (22.2)	0	1	0	1	0	0
	Blood creatine phosphokinase increased	1 (11.1)	0	1	0	0	0	0
	C-reactive protein increased	1 (11.1)	0	0	1	0	0	0
	Gamma-glutamyltransferase increased	2 (22.2)	0	1	0	1	0	0
	Weight decreased	1 (11.1)	0	1	0	0	0	0
Metabolism and nutrition disorders	Patients with one or more Events	2 (22.2)	0	1	1	0	0	0
	Decreased appetite	1 (11.1)	0	0	1	0	0	0
	Hypokalaemia	1 (11.1)	0	1	0	0	0	0
	Hyponatraemia	1 (11.1)	0	1	0	0	0	0
Nervous system disorders	Patients with one or more Events	8 (88.9)	0	1	4	3	0	0
	Aphasia	1 (11.1)	0	0	1	0	0	0
	Apraxia	2 (22.2)	0	1	1	0	0	0
	Ataxia	1 (11.1)	0	0	1	0	0	0
	Cognitive disorder	2 (22.2)	0	2	0	0	0	0
	Coordination abnormal	1 (11.1)	0	1	0	0	0	0
	Disturbance in attention	2 (22.2)	0	1	1	0	0	0
	Dysgeusia	1 (11.1)	0	0	1	0	0	0
	Facial paresis	1 (11.1)	0	1	0	0	0	0
	Headache	2 (22.2)	0	1	1	0	0	0
	Hemiparesis	3 (33.3)	0	2	1	0	0	0
	Memory impairment	2 (22.2)	0	0	2	0	0	0
	Neurological decompensation	1 (11.1)	0	0	0	1	0	0
	Seizure	3 (33.3)	0	0	1	2	0	0

The AE with the highest grading for each preferred term for each patient was taken. A patient who reports two or more Different preferred terms which are in the same system organ class, is counted only once in the system organ class total.
The highest grading of this patient in the respective system organ class will be taken.

Section 13 Table 6.1.2.2.1

Incidence and Severity of Adverse Events (All Causalities) - Safety Population Phase 1-Dosegroup: All Dosegroups

AEs that occured on or after the date of first medication and before end of observation have been considered as treament emergent AEs.

		N=9						
			Severity					
System organ class (MedDRA 23.0)	Preferred Term (MedDRA 23.0)	Number of patients (%) *	Missing	Grade 1	Grade 2	Grade 3	Grade 4	Grade 5
Psychiatric disorders	Patients with one or more Events	4 (44.4)	0	1	2	1	0	0
	Anxiety	1 (11.1)	0	0	0	1	0	0
	Confusional state	1 (11.1)	0	1	0	0	0	0
	Depressed mood	1 (11.1)	0	0	1	0	0	0
	Disorientation	1 (11.1)	0	0	1	0	0	0
	Psychomotor retardation	1 (11.1)	0	1	0	0	0	0
Renal and urinary disorders	Patients with one or more Events	1 (11.1)	0	0	1	0	0	0
	Incontinence	1 (11.1)	0	0	1	0	0	0
Respiratory, thoracic and mediastinal disorders	Patients with one or more Events	1 (11.1)	0	1	0	0	0	0
	Oropharyngeal pain	1 (11.1)	0	1	0	0	0	0
Skin and subcutaneous tissue disorders	Patients with one or more Events	4 (44.4)	0	2	2	0	0	0
	Alopecia	4 (44.4)	0	2	2	0	0	0
Vascular disorders	Patients with one or more Events	2 (22.2)	0	1	1	0	0	0
	Hypotension	1 (11.1)	0	0	1	0	0	0
	Thrombosis	1 (11.1)	0	1	0	0	0	0

The AE with the highest grading for each preferred term for each patient was taken. A patient who reports two or more Different preferred terms which are in the same system organ class, is counted only once in the system organ class total.
The highest grading of this patient in the respective system organ class will be taken.

Section 13 Table 6.1.2.1
Incidence and Severity of Adverse Events (All Causalities) - Safety Population P1
Dosegroup=All Dosegroups

AEs that occured on or after the date of first medication and before end of observation have been considered as treament emergent AEs.

		N=9						
			Severity					
System organ class (MedDRA 23.0)	Preferred Term (MedDRA 23.0)	Number of patients (%) *	Missing	Grade 1	Grade 2	Grade 3	Grade 4	Grade 5
Blood and lymphatic system disorders	Patients with one or more Events	7 (77.8)	0	1	2	1	3	0
	Anaemia	1 (11.1)	0	1	0	0	0	0
	Leukopenia	6 (66.7)	0	1	3	1	1	0
	Lymphopenia	2 (22.2)	0	1	1	0	0	0
	Neutropenia	4 (44.4)	0	0	0	1	3	0
	Thrombocytopenia	5 (55.6)	0	0	1	2	2	0
Ear and labyrinth disorders	Patients with one or more Events	1 (11.1)	0	0	1	0	0	0
	Ear pain	1 (11.1)	0	0	1	0	0	0
Eye disorders	Patients with one or more Events	2 (22.2)	0	1	1	0	0	0
	Eyelid thickening	1 (11.1)	0	1	0	0	0	0
	Visual impairment	1 (11.1)	0	0	1	0	0	0
Gastrointestinal disorders	Patients with one or more Events	9 (100)	0	3	6	0	0	0
	Constipation	1 (11.1)	0	1	0	0	0	0
	Diarrhoea	7 (77.8)	0	3	4	0	0	0
	Nausea	8 (88.9)	0	3	5	0	0	0
	Vomiting	6 (66.7)	0	1	5	0	0	0
General disorders and administration site conditions	Patients with one or more Events	9 (100)	0	4	5	0	0	0
	Asthenia	1 (11.1)	0	1	0	0	0	0
	Fatigue	8 (88.9)	0	3	5	0	0	0
	Influenza like illness	1 (11.1)	0	1	0	0	0	0
	Malaise	2 (22.2)	0	2	0	0	0	0
	Oedema	1 (11.1)	0	0	1	0	0	0
	Oedema peripheral	1 (11.1)	0	1	0	0	0	0
	Pyrexia	1 (11.1)	0	1	0	0	0	0

Patients are assigned to dose groups according to their respective initial start Dose.
The AE with the highest grading for each preferred term for each patient was taken. A patient who reports two or more different preferred terms which are in the same system organ class, is counted only once in the system organ class total.
The highest grading of this patient in the respective system organ class will be taken.

AEs that occured on or after the date of first medication and before end of observation have been considered as treament emergent AEs.

		N=9						
			Severity					
System organ class (MedDRA 23.0)	Preferred Term (MedDRA 23.0)	Number of patients (%) *	Missing	Grade 1	Grade 2	Grade 3	Grade 4	Grade 5
Infections and infestations	Patients with one or more Events	2 (22.2)	0	1	1	0	0	0
	Gingivitis	1 (11.1)	0	1	0	0	0	0
	Laryngitis	1 (11.1)	0	0	1	0	0	0
Investigations	Patients with one or more Events	4 (44.4)	0	1	1	1	1	0
	Alanine aminotransferase increased	1 (11.1)	0	1	0	0	0	0
	Aspartate aminotransferase increased	1 (11.1)	0	1	0	0	0	0
	Blood creatine phosphokinase increased	1 (11.1)	0	1	0	0	0	0
	C-reactive protein increased	1 (11.1)	0	1	0	0	0	0
	Gamma-glutamyltransferase increased	1 (11.1)	0	0	1	0	0	0
	Neutrophil count decreased	2 (22.2)	0	0	0	2	0	0
	Platelet count decreased	2 (22.2)	0	0	0	1	1	0
	White blood cell count decreased	2 (22.2)	0	0	1	1	0	0
Metabolism and nutrition disorders	Patients with one or more Events	6 (66.7)	0	1	5	0	0	0
	Decreased appetite	5 (55.6)	0	1	4	0	0	0
	Hyperkalaemia	1 (11.1)	0	0	1	0	0	0
	Hypokalaemia	2 (22.2)	0	2	0	0	0	0
Musculoskeletal and connective tissue disorders	Patients with one or more Events	1 (11.1)	0	0	1	0	0	0
	Neck pain	1 (11.1)	0	0	1	0	0	0
Nervous system disorders	Patients with one or more Events	8 (88.9)	0	2	5	1	0	0
	Ageusia	1 (11.1)	0	0	1	0	0	0
	Aphasia	1 (11.1)	0	1	0	0	0	0
	Ataxia	1 (11.1)	0	1	0	0	0	0
	Burning sensation	1 (11.1)	0	0	1	0	0	0
	Dizziness	1 (11.1)	0	0	1	0	0	0

Patients are assigned to dose groups according to their respective initial start Dose.
The AE with the highest grading for each preferred term for each patient was taken. A patient who reports two or more different preferred terms which are in the same system organ class, is counted only once in the system organ class total.
The highest grading of this patient in the respective system organ class will be taken.

Section 13 Table 6.1.2.1
Incidence and Severity of Adverse Events (All Causalities) - Safety Population P1
Dosegroup=All Dosegroups

AEs that occured on or after the date of first medication and before end of observation have been considered as treament emergent AEs.

		N=9						
			Severity					
System organ class (MedDRA 23.0)	Preferred Term (MedDRA 23.0)	Number of patients (%) *	Missing	Grade 1	Grade 2	Grade 3	Grade 4	Grade 5
Nervous system disorders	Headache	3 (33.3)	0	0	3	0	0	0
	Hemiparesis	1 (11.1)	0	0	1	0	0	0
	Hypertonia	1 (11.1)	0	1	0	0	0	0
	Hypoaesthesia	1 (11.1)	0	1	0	0	0	0
	Hypotonia	1 (11.1)	0	1	0	0	0	0
	Memory impairment	1 (11.1)	0	0	1	0	0	0
	Monoparesis	1 (11.1)	0	1	0	0	0	0
	Quadrantanopia	1 (11.1)	0	1	0	0	0	0
	Seizure	2 (22.2)	0	0	1	1	0	0
	Tension headache	1 (11.1)	0	0	1	0	0	0
	Tremor	1 (11.1)	0	0	1	0	0	0
Psychiatric disorders	Patients with one or more Events	1 (11.1)	0	0	1	0	0	0
	Confusional state	1 (11.1)	0	0	1	0	0	0
	Disorientation	1 (11.1)	0	0	1	0	0	0
Respiratory, thoracic and mediastinal disorders	Patients with one or more Events	1 (11.1)	0	1	0	0	0	0
	Dyspnoea	1 (11.1)	0	1	0	0	0	0
Skin and subcutaneous tissue disorders	Patients with one or more Events	5 (55.6)	0	2	3	0	0	0
	Alopecia	4 (44.4)	0	4	0	0	0	0
	Dry skin	1 (11.1)	0	1	0	0	0	0
	Erythema	3 (33.3)	0	0	3	0	0	0
	Skin swelling	1 (11.1)	0	0	1	0	0	0

Patients are assigned to dose groups according to their respective initial start Dose.
The AE with the highest grading for each preferred term for each patient was taken. A patient who reports two or more different preferred terms which are in the same system organ class, is counted only once in the system organ class total.
The highest grading of this patient in the respective system organ class will be taken.

Incidence and Severity of Adverse Events (All Causalities) - Full Analysis Set

AEs that occured on or after the date of first medication and before end of observation have been considered as treament emergent AEs.

		N=42						
			Severity					
System organ class (MedDRA 23.0)	Preferred Term (MedDRA 23.0)	Number of patients (%) *	Missing	Grade 1	Grade 2	Grade 3	Grade 4	Grade 5
Blood and lymphatic system disorders	Patients with one or more Events	11 (26.2)	0	2	5	3	1	0
	Anaemia	1 (2.4)	0	1	0	0	0	0
	Leukopenia	2 (4.8)	0	0	2	0	0	0
	Lymphopenia	7 (16.7)	0	1	4	2	0	0
	Monocytosis	1 (2.4)	0	1	0	0	0	0
	Neutropenia	1 (2.4)	0	0	0	1	0	0
	Thrombocytopenia	1 (2.4)	0	0	0	0	1	0
Cardiac disorders	Patients with one or more Events	2 (4.8)	0	2	0	0	0	0
	Bradycardia	1 (2.4)	0	1	0	0	0	0
	Bundle branch block right	1 (2.4)	0	1	0	0	0	0
Ear and labyrinth disorders	Patients with one or more Events	2 (4.8)	0	1	1	0	0	0
	Ear disorder	1 (2.4)	0	1	0	0	0	0
	Middle ear effusion	1 (2.4)	0	0	1	0	0	0
	Vertigo	1 (2.4)	0	1	0	0	0	0
Endocrine disorders	Patients with one or more Events	2 (4.8)	0	0	2	0	0	0
	Hypothyroidism	2 (4.8)	0	0	2	0	0	0
Eye disorders	Patients with one or more Events	5 (11.9)	0	3	2	0	0	0
	Conjunctival irritation	1 (2.4)	0	1	0	0	0	0
	Eye irritation	1 (2.4)	0	1	0	0	0	0
	Vision blurred	1 (2.4)	0	1	0	0	0	0
	Visual impairment	2 (4.8)	0	0	2	0	0	0
Gastrointestinal disorders	Patients with one or more Events	15 (35.7)	0	9	6	0	0	0
	Constipation	2 (4.8)	0	1	1	0	0	0
	Diarrhoea	5 (11.9)	0	4	1	0	0	0
	Flatulence	1 (2.4)	0	1	0	0	0	0
	Nausea	9 (21.4)	0	4	5	0	0	0
	Stomatitis	1 (2.4)	0	1	0	0	0	0

The AE with the highest grading for each preferred term for each patient was taken. A patient who reports two or more Different preferred terms which are in the same system organ class, is counted only once in the system organ class total.
The highest grading of this patient in the respective system organ class will be taken.

Section 13 Table 6.1.2.2

Incidence and Severity of Adverse Events (All Causalities) - Full Analysis Set

AEs that occured on or after the date of first medication and before end of observation have been considered as treament emergent AEs.

		N=42						
			Severity					
System organ class (MedDRA 23.0)	Preferred Term (MedDRA 23.0)	Number of patients (%) *	Missing	Grade 1	Grade 2	Grade 3	Grade 4	Grade 5
Gastrointestinal disorders	Vomiting	4 (9.5)	0	2	2	0	0	0
General disorders and administration site conditions	Patients with one or more Events	24 (57.1)	1	11	10	2	0	0
	Asthenia	1 (2.4)	0	0	1	0	0	0
	Chills	2 (4.8)	0	2	0	0	0	0
	Disease progression	1 (2.4)	0	0	0	1	0	0
	Fatigue	9 (21.4)	0	6	3	0	0	0
	Gait disturbance	3 (7.1)	0	2	1	0	0	0
	General physical health deterioration	4 (9.5)	1	0	2	1	0	0
	Granuloma	1 (2.4)	0	0	1	0	0	0
	Hernia pain	1 (2.4)	0	1	0	0	0	0
	Impaired healing	1 (2.4)	0	0	0	1	0	0
	Influenza like illness	1 (2.4)	0	1	0	0	0	0
	Oedema	2 (4.8)	0	1	1	0	0	0
	Peripheral swelling	2 (4.8)	0	2	0	0	0	0
	Pyrexia	5 (11.9)	0	3	2	0	0	0
	Swelling	1 (2.4)	0	0	1	0	0	0
	Swelling face	1 (2.4)	0	1	0	0	0	0
Hepatobiliary disorders	Patients with one or more Events	4 (9.5)	0	0	0	3	1	0
	Autoimmune hepatitis	1 (2.4)	0	0	0	1	0	0
	Drug-induced liver injury	1 (2.4)	0	0	0	1	0	0
	Hepatitis	2 (4.8)	0	0	0	1	1	0
Infections and infestations	Patients with one or more Events	15 (35.7)	0	4	7	4	0	0
	Acute sinusitis	1 (2.4)	0	0	0	1	0	0
	COVID-19	2 (4.8)	0	0	1	1	0	0
	Candida infection	1 (2.4)	0	1	0	0	0	0
	Herpes simplex	1 (2.4)	0	1	0	0	0	0
	Infection	2 (4.8)	0	0	0	2	0	0

The AE with the highest grading for each preferred term for each patient was taken. A patient who reports two or more Different preferred terms which are in the same system organ class, is counted only once in the system organ class total.
The highest grading of this patient in the respective system organ class will be taken.

Incidence and Severity of Adverse Events (All Causalities) - Full Analysis Set

AEs that occured on or after the date of first medication and before end of observation have been considered as treament emergent AEs.

		N=42						
			Severity					
System organ class (MedDRA 23.0)	Preferred Term (MedDRA 23.0)	Number of patients (%) *	Missing	Grade 1	Grade 2	Grade 3	Grade 4	Grade 5
Infections and infestations	Oral herpes	1 (2.4)	0	1	0	0	0	0
	Pneumonia	1 (2.4)	0	0	1	0	0	0
	Sinusitis	1 (2.4)	0	0	1	0	0	0
	Tinea versicolour	1 (2.4)	0	1	0	0	0	0
	Upper respiratory tract infection	1 (2.4)	0	1	0	0	0	0
	Urinary tract infection	5 (11.9)	0	1	4	0	0	0
	Viral infection	1 (2.4)	0	0	1	0	0	0
Injury, poisoning and procedural complications	Patients with one or more Events	5 (11.9)	0	4	0	0	1	0
	Contusion	1 (2.4)	0	1	0	0	0	0
	Extradural haematoma	1 (2.4)	0	0	0	0	1	0
	Fall	1 (2.4)	0	1	0	0	0	0
	Radiation alopecia	1 (2.4)	0	1	0	0	0	0
	Radiation skin injury	1 (2.4)	0	1	0	0	0	0
Investigations	Patients with one or more Events	16 (38.1)	0	4	9	3	0	0
	Alanine aminotransferase increased	5 (11.9)	0	1	3	1	0	0
	Aspartate aminotransferase increased	4 (9.5)	0	2	1	1	0	0
	Bilirubin conjugated increased	1 (2.4)	0	1	0	0	0	0
	Blood alkaline phosphatase increased	2 (4.8)	0	1	1	0	0	0
	Blood bilirubin increased	4 (9.5)	0	3	1	0	0	0
	Blood lactate dehydrogenase increased	1 (2.4)	0	1	0	0	0	0
	Blood thyroid stimulating hormone increased	1 (2.4)	0	1	0	0	0	0
	C-reactive protein increased	6 (14.3)	0	4	2	0	0	0
	Creatinine renal clearance decreased	1 (2.4)	0	0	1	0	0	0

The AE with the highest grading for each preferred term for each patient was taken. A patient who reports two or more Different preferred terms which are in the same system organ class, is counted only once in the system organ class total.
The highest grading of this patient in the respective system organ class will be taken.

Incidence and Severity of Adverse Events (All Causalities) - Full Analysis Set

AEs that occured on or after the date of first medication and before end of observation have been considered as treament emergent AEs.

		N=42						
			Severity					
System organ class (MedDRA 23.0)	Preferred Term (MedDRA 23.0)	Number of patients (%) *	Missing	Grade 1	Grade 2	Grade 3	Grade 4	Grade 5
Investigations	Gamma-glutamyltransferase increased	3 (7.1)	0	1	1	1	0	0
	Liver function test increased	1 (2.4)	0	0	1	0	0	0
	Lymphocyte count decreased	4 (9.5)	0	0	4	0	0	0
	Lymphocyte count increased	1 (2.4)	0	0	1	0	0	0
	Nitrite urine present	1 (2.4)	0	0	1	0	0	0
	Urobilinogen urine increased	1 (2.4)	0	1	0	0	0	0
	Vitamin B1 decreased	1 (2.4)	0	1	0	0	0	0
	Weight decreased	1 (2.4)	0	0	0	1	0	0
	Weight increased	1 (2.4)	0	1	0	0	0	0
	White blood cell count decreased	1 (2.4)	0	0	1	0	0	0
Metabolism and nutrition disorders	Patients with one or more Events	9 (21.4)	0	5	4	0	0	0
	Decreased appetite	5 (11.9)	0	3	2	0	0	0
	Diabetes mellitus	1 (2.4)	0	1	0	0	0	0
	Hypercholesterolaemia	1 (2.4)	0	0	1	0	0	0
	Hyperglycaemia	1 (2.4)	0	0	1	0	0	0
	Hypokalaemia	1 (2.4)	0	1	0	0	0	0
	Hypomagnesaemia	1 (2.4)	0	1	0	0	0	0
	Steroid diabetes	1 (2.4)	0	0	1	0	0	0
Musculoskeletal and connective tissue disorders	Patients with one or more Events	10 (23.8)	0	7	3	0	0	0
	Arthralgia	2 (4.8)	0	1	1	0	0	0
	Back pain	3 (7.1)	0	1	2	0	0	0
	Muscular weakness	1 (2.4)	0	1	0	0	0	0
	Musculoskeletal pain	2 (4.8)	0	2	0	0	0	0
	Myalgia	1 (2.4)	0	1	0	0	0	0
	Pain in extremity	1 (2.4)	0	1	0	0	0	0

The AE with the highest grading for each preferred term for each patient was taken. A patient who reports two or more Different preferred terms which are in the same system organ class, is counted only once in the system organ class total.
The highest grading of this patient in the respective system organ class will be taken.

Incidence and Severity of Adverse Events (All Causalities) - Full Analysis Set

AEs that occured on or after the date of first medication and before end of observation have been considered as treament emergent AEs.

		N=42						
			Severity					
**System organ class (MedDRA 23.0)**	**Preferred Term (MedDRA 23.0)**	**Number of patients (%) ***	**Missing**	**Grade 1**	**Grade 2**	**Grade 3**	**Grade 4**	**Grade 5**
Nervous system disorders	Patients with one or more Events	23 (54.8)	1	7	9	6	0	0
	Ageusia	1 (2.4)	0	1	0	0	0	0
	Aphasia	5 (11.9)	0	0	4	1	0	0
	Brain oedema	2 (4.8)	0	0	2	0	0	0
	Cerebral venous sinus thrombosis	1 (2.4)	0	0	0	1	0	0
	Cognitive disorder	1 (2.4)	0	0	1	0	0	0
	Dizziness	1 (2.4)	0	1	0	0	0	0
	Dysaesthesia	1 (2.4)	0	0	1	0	0	0
	Dysarthria	1 (2.4)	0	1	0	0	0	0
	Fine motor skill dysfunction	1 (2.4)	0	1	0	0	0	0
	Headache	10 (23.8)	1	2	6	1	0	0
	Hemianaesthesia	1 (2.4)	0	1	0	0	0	0
	Hemiparesis	1 (2.4)	0	0	0	1	0	0
	Hydrocephalus	1 (2.4)	1	0	0	0	0	0
	Hypoaesthesia	2 (4.8)	0	1	1	0	0	0
	Intracranial pressure increased	1 (2.4)	0	0	0	1	0	0
	Ischaemic stroke	1 (2.4)	0	0	1	0	0	0
	Memory impairment	1 (2.4)	0	1	0	0	0	0
	Neurologic neglect syndrome	1 (2.4)	0	1	0	0	0	0
	Neuropathy peripheral	1 (2.4)	0	0	1	0	0	0
	Paresis	1 (2.4)	0	0	1	0	0	0
	Seizure	6 (14.3)	0	2	2	2	0	0
	Sensory disturbance	1 (2.4)	0	1	0	0	0	0
	Somnolence	1 (2.4)	0	0	0	1	0	0
	Wernicke's encephalopathy	1 (2.4)	0	0	0	1	0	0

The AE with the highest grading for each preferred term for each patient was taken. A patient who reports two or more Different preferred terms which are in the same system organ class, is counted only once in the system organ class total.
The highest grading of this patient in the respective system organ class will be taken.

Section 13 Table 6.1.2.2

Incidence and Severity of Adverse Events (All Causalities) - Full Analysis Set

AEs that occured on or after the date of first medication and before end of observation have been considered as treament emergent AEs.

		N=42						
			Severity					
System organ class (MedDRA 23.0)	Preferred Term (MedDRA 23.0)	Number of patients (%) *	Missing	Grade 1	Grade 2	Grade 3	Grade 4	Grade 5
Psychiatric disorders	Patients with one or more Events	4 (9.5)	0	0	2	2	0	0
	Agitation	1 (2.4)	0	0	1	0	0	0
	Confusional state	1 (2.4)	0	0	0	1	0	0
	Depressed mood	1 (2.4)	0	0	1	0	0	0
	Depression	1 (2.4)	0	0	1	0	0	0
	Disorientation	1 (2.4)	0	0	0	1	0	0
	Panic attack	1 (2.4)	0	0	1	0	0	0
	Personality change	1 (2.4)	0	0	1	0	0	0
Renal and urinary disorders	Patients with one or more Events	2 (4.8)	0	1	1	0	0	0
	Chromaturia	1 (2.4)	0	1	0	0	0	0
	Haematuria	1 (2.4)	0	1	0	0	0	0
	Renal disorder	1 (2.4)	0	1	0	0	0	0
	Urinary incontinence	1 (2.4)	0	0	1	0	0	0
	Urinary tract disorder	1 (2.4)	0	1	0	0	0	0
Reproductive system and breast disorders	Patients with one or more Events	1 (2.4)	0	1	0	0	0	0
	Benign prostatic hyperplasia	1 (2.4)	0	1	0	0	0	0
Respiratory, thoracic and mediastinal disorders	Patients with one or more Events	2 (4.8)	0	2	0	0	0	0
	Cough	1 (2.4)	0	1	0	0	0	0
	Nasal congestion	1 (2.4)	0	1	0	0	0	0
Skin and subcutaneous tissue disorders	Patients with one or more Events	18 (42.9)	0	11	7	0	0	0
	Alopecia	10 (23.8)	0	7	3	0	0	0
	Dermatitis	1 (2.4)	0	1	0	0	0	0
	Dermatitis acneiform	1 (2.4)	0	0	1	0	0	0
	Eczema	1 (2.4)	0	0	1	0	0	0
	Erythema	2 (4.8)	0	2	0	0	0	0
	Pruritus	1 (2.4)	0	0	1	0	0	0

The AE with the highest grading for each preferred term for each patient was taken. A patient who reports two or more Different preferred terms which are in the same system organ class, is counted only once in the system organ class total.
The highest grading of this patient in the respective system organ class will be taken.

Incidence and Severity of Adverse Events (All Causalities) - Full Analysis Set

AEs that occured on or after the date of first medication and before end of observation have been considered as treament emergent AEs.

		N=42						
			Severity					
System organ class (MedDRA 23.0)	Preferred Term (MedDRA 23.0)	Number of patients (%) *	Missing	Grade 1	Grade 2	Grade 3	Grade 4	Grade 5
Skin and subcutaneous tissue disorders	Psoriasis	1 (2.4)	0	1	0	0	0	0
	Rash	1 (2.4)	0	0	1	0	0	0
	Rash maculo-papular	1 (2.4)	0	1	0	0	0	0
	Urticaria	1 (2.4)	0	0	1	0	0	0
	Urticaria chronic	1 (2.4)	0	0	1	0	0	0
Surgical and medical procedures	Patients with one or more Events	1 (2.4)	0	0	1	0	0	0
	Endodontic procedure	1 (2.4)	0	0	1	0	0	0
	Tumour excision	1 (2.4)	0	1	0	0	0	0
Vascular disorders	Patients with one or more Events	3 (7.1)	0	1	0	2	0	0
	Embolism	1 (2.4)	0	0	0	1	0	0
	Flushing	1 (2.4)	0	1	0	0	0	0
	Hypotension	1 (2.4)	0	1	0	0	0	0
	Jugular vein thrombosis	1 (2.4)	0	0	0	1	0	0

The AE with the highest grading for each preferred term for each patient was taken. A patient who reports two or more Different preferred terms which are in the same system organ class, is counted only once in the system organ class total.
The highest grading of this patient in the respective system organ class will be taken.

Protocol: EudraCT NO: 2015-002752-27 DRAFT REPORT
Subtrial F: Palbociclib
Section 13 Table 6.1.2.1.1
Incidence and Severity of Adverse Events (All Causalities) - Safety Population Phase 1 - Dosegroup: All Dosegroups

AEs that occured on or after the date of first medication and before end of observation have been considered as treament emergent AEs.

		N=13						
			Severity					
System organ class (MedDRA 23.0)	Preferred Term (MedDRA 23.0)	Number of patients (%) *	Missing	Grade 1	Grade 2	Grade 3	Grade 4	Grade 5
Blood and lymphatic system disorders	Patients with one or more Events	8 (61.5)	0	2	1	2	3	0
	Anaemia	1 (7.7)	0	0	1	0	0	0
	Leukopenia	4 (30.8)	0	1	1	2	0	0
	Lymphopenia	5 (38.5)	0	0	1	3	1	0
	Neutropenia	4 (30.8)	0	0	1	1	2	0
	Thrombocytopenia	3 (23.1)	0	1	2	0	0	0
Ear and labyrinth disorders	Patients with one or more Events	3 (23.1)	0	3	0	0	0	0
	Hypoacusis	1 (7.7)	0	1	0	0	0	0
	Tinnitus	1 (7.7)	0	1	0	0	0	0
	Vertigo	1 (7.7)	0	1	0	0	0	0
Eye disorders	Patients with one or more Events	2 (15.4)	0	2	0	0	0	0
	Eye disorder	1 (7.7)	0	1	0	0	0	0
	Eye oedema	1 (7.7)	0	1	0	0	0	0
	Vision blurred	1 (7.7)	0	1	0	0	0	0
Gastrointestinal disorders	Patients with one or more Events	6 (46.2)	0	1	5	0	0	0
	Aphthous ulcer	1 (7.7)	0	0	1	0	0	0
	Constipation	2 (15.4)	0	1	1	0	0	0
	Gastritis	1 (7.7)	0	0	1	0	0	0
	Nausea	2 (15.4)	0	0	2	0	0	0
	Stomatitis	1 (7.7)	0	0	1	0	0	0
General disorders and administration site conditions	Patients with one or more Events	7 (53.8)	0	3	3	1	0	0
	Fatigue	4 (30.8)	0	3	1	0	0	0
	General physical health deterioration	1 (7.7)	0	0	1	0	0	0
	Hernia	1 (7.7)	0	0	0	1	0	0
	Oedema	1 (7.7)	0	0	1	0	0	0
	Pain	1 (7.7)	0	0	1	0	0	0

The AE with the highest grading for each preferred term for each patient was taken. A patient who reports two or more Different preferred terms which are in the same system organ class, is counted only once in the system organ class total.
The highest grading of this patient in the respective system organ class will be taken.

Section 13 Table 6.1.2.1.1

Incidence and Severity of Adverse Events (All Causalities) - Safety Population Phase 1 - Dosegroup: All Dosegroups

AEs that occured on or after the date of first medication and before end of observation have been considered as treament emergent AEs.

		N=13						
			Severity					
System organ class (MedDRA 23.0)	Preferred Term (MedDRA 23.0)	Number of patients (%) *	Missing	Grade 1	Grade 2	Grade 3	Grade 4	Grade 5
General disorders and administration site conditions	Swelling face	1 (7.7)	0	1	0	0	0	0
Immune system disorders	Patients with one or more Events	1 (7.7)	0	0	1	0	0	0
	Hypersensitivity	1 (7.7)	0	0	1	0	0	0
Infections and infestations	Patients with one or more Events	5 (38.5)	0	1	4	0	0	0
	Cystitis	2 (15.4)	0	1	1	0	0	0
	Lip infection	1 (7.7)	0	0	1	0	0	0
	Nasopharyngitis	2 (15.4)	0	1	1	0	0	0
	Urinary tract infection	1 (7.7)	0	0	1	0	0	0
Injury, poisoning and procedural complications	Patients with one or more Events	1 (7.7)	0	1	0	0	0	0
	Post procedural erythema	1 (7.7)	0	1	0	0	0	0
Investigations	Patients with one or more Events	8 (61.5)	0	2	3	2	1	0
	Alanine aminotransferase increased	2 (15.4)	0	0	1	1	0	0
	Aspartate aminotransferase increased	2 (15.4)	0	1	1	0	0	0
	Bilirubin conjugated increased	1 (7.7)	0	1	0	0	0	0
	Blood bilirubin increased	1 (7.7)	0	0	1	0	0	0
	Blood creatinine increased	1 (7.7)	0	1	0	0	0	0
	C-reactive protein increased	1 (7.7)	0	1	0	0	0	0
	Lymphocyte count decreased	1 (7.7)	0	0	0	0	1	0
	Neutrophil count decreased	3 (23.1)	0	0	2	1	0	0
	Weight decreased	2 (15.4)	0	1	1	0	0	0
	White blood cell count decreased	2 (15.4)	0	0	2	0	0	0

The AE with the highest grading for each preferred term for each patient was taken. A patient who reports two or more Different preferred terms which are in the same system organ class, is counted only once in the system organ class total.
The highest grading of this patient in the respective system organ class will be taken.

Section 13 Table 6.1.2.1.1
Incidence and Severity of Adverse Events (All Causalities) - Safety Population Phase 1 - Dosegroup: All Dosegroups

AEs that occured on or after the date of first medication and before end of observation have been considered as treament emergent AEs.

		N=13						
			Severity					
**System organ class (MedDRA 23.0)**	**Preferred Term (MedDRA 23.0)**	**Number of patients (%) ***	**Missing**	**Grade 1**	**Grade 2**	**Grade 3**	**Grade 4**	**Grade 5**
Metabolism and nutrition disorders	Patients with one or more Events	2 (15.4)	0	1	0	0	1	0
	Decreased appetite	1 (7.7)	0	1	0	0	0	0
	Hypochloraemia	1 (7.7)	0	1	0	0	0	0
	Hyponatraemia	1 (7.7)	0	0	0	0	1	0
Musculoskeletal and connective tissue disorders	Patients with one or more Events	2 (15.4)	0	1	0	1	0	0
	Back pain	1 (7.7)	0	0	0	1	0	0
	Soft tissue swelling	1 (7.7)	0	1	0	0	0	0
Nervous system disorders	Patients with one or more Events	9 (69.2)	0	5	2	1	1	0
	Aphasia	1 (7.7)	0	1	0	0	0	0
	Apraxia	1 (7.7)	0	1	0	0	0	0
	Cold-stimulus headache	1 (7.7)	0	1	0	0	0	0
	Dizziness	1 (7.7)	0	0	1	0	0	0
	Facial paresis	1 (7.7)	0	1	0	0	0	0
	Headache	4 (30.8)	0	1	3	0	0	0
	Hemiparesis	1 (7.7)	0	0	0	1	0	0
	Memory impairment	2 (15.4)	0	2	0	0	0	0
	Neurologic neglect syndrome	1 (7.7)	0	1	0	0	0	0
	Neurological decompensation	1 (7.7)	0	0	0	1	0	0
	Seizure	2 (15.4)	0	1	0	0	1	0
Psychiatric disorders	Patients with one or more Events	2 (15.4)	0	1	1	0	0	0
	Insomnia	1 (7.7)	0	0	1	0	0	0
	Sleep disorder	1 (7.7)	0	1	0	0	0	0
Respiratory, thoracic and mediastinal disorders	Patients with one or more Events	2 (15.4)	0	0	1	1	0	0
	Cough	1 (7.7)	0	0	1	0	0	0
	Pulmonary embolism	1 (7.7)	0	0	0	1	0	0

The AE with the highest grading for each preferred term for each patient was taken. A patient who reports two or more Different preferred terms which are in the same system organ class, is counted only once in the system organ class total.
The highest grading of this patient in the respective system organ class will be taken.

Section 13 Table 6.1.2.1.1
Incidence and Severity of Adverse Events (All Causalities) - Safety Population Phase 1 - Dosegroup: All Dosegroups

AEs that occured on or after the date of first medication and before end of observation have been considered as treament emergent AEs.

		N=13						
			Severity					
System organ class (MedDRA 23.0)	Preferred Term (MedDRA 23.0)	Number of patients (%) *	Missing	Grade 1	Grade 2	Grade 3	Grade 4	Grade 5
Skin and subcutaneous tissue disorders	Patients with one or more Events	8 (61.5)	0	4	4	0	0	0
	Alopecia	6 (46.2)	0	3	3	0	0	0
	Erythema	2 (15.4)	0	2	0	0	0	0
	Erythema multiforme	1 (7.7)	0	0	1	0	0	0
	Rosacea	1 (7.7)	0	1	0	0	0	0
Vascular disorders	Patients with one or more Events	1 (7.7)	0	0	1	0	0	0
	Hypertension	1 (7.7)	0	0	1	0	0	0

The AE with the highest grading for each preferred term for each patient was taken. A patient who reports two or more Different preferred terms which are in the same system organ class, is counted only once in the system organ class total.
The highest grading of this patient in the respective system organ class will be taken.

Section 13 Table 6.1.2.2.1
Incidence and Severity of Adverse Events (All Causalities) - Full Analysis Set - Dosegroup: All Dosegroups

AEs that occured on or after the date of first medication and before end of observation have been considered as treament emergent AEs.

		N=41						
			Severity					
System organ class (MedDRA 23.0)	Preferred Term (MedDRA 23.0)	Number of patients (%) *	Missing	Grade 1	Grade 2	Grade 3	Grade 4	Grade 5
Blood and lymphatic system disorders	Patients with one or more Events	20 (48.8)	0	2	8	8	2	0
	Anaemia	2 (4.9)	0	1	1	0	0	0
	Leukopenia	13 (31.7)	0	1	9	3	0	0
	Lymphopenia	11 (26.8)	0	0	3	6	2	0
	Neutropenia	3 (7.3)	0	0	2	1	0	0
	Thrombocytopenia	5 (12.2)	0	2	3	0	0	0
Cardiac disorders	Patients with one or more Events	2 (4.9)	0	1	0	1	0	0
	Angina pectoris	1 (2.4)	0	0	0	1	0	0
	Arrhythmia	1 (2.4)	0	1	0	0	0	0
Ear and labyrinth disorders	Patients with one or more Events	7 (17.1)	0	5	2	0	0	0
	Hypoacusis	1 (2.4)	0	1	0	0	0	0
	Otorrhoea	1 (2.4)	0	1	0	0	0	0
	Tinnitus	3 (7.3)	0	1	2	0	0	0
	Vertigo	2 (4.9)	0	2	0	0	0	0
Endocrine disorders	Patients with one or more Events	1 (2.4)	0	1	0	0	0	0
	Cushing's syndrome	1 (2.4)	0	1	0	0	0	0
Eye disorders	Patients with one or more Events	9 (22.0)	0	8	1	0	0	0
	Dry eye	3 (7.3)	0	2	1	0	0	0
	Eye disorder	1 (2.4)	0	1	0	0	0	0
	Eye oedema	1 (2.4)	0	1	0	0	0	0
	Eyelid function disorder	1 (2.4)	0	1	0	0	0	0
	Glare	1 (2.4)	0	1	0	0	0	0
	Vision blurred	2 (4.9)	0	2	0	0	0	0
	Visual impairment	1 (2.4)	0	1	0	0	0	0

The AE with the highest grading for each preferred term for each patient was taken. A patient who reports two or more Different preferred terms which are in the same system organ class, is counted only once in the system organ class total.
The highest grading of this patient in the respective system organ class will be taken.

Section 13 Table 6.1.2.2.1

Incidence and Severity of Adverse Events (All Causalities) - Full Analysis Set - Dosegroup: All Dosegroups

AEs that occured on or after the date of first medication and before end of observation have been considered as treament emergent AEs.

		N=41						
			Severity					
System organ class (MedDRA 23.0)	Preferred Term (MedDRA 23.0)	Number of patients (%) *	Missing	Grade 1	Grade 2	Grade 3	Grade 4	Grade 5
Gastrointestinal disorders	Patients with one or more Events	18 (43.9)	0	11	7	0	0	0
	Abdominal discomfort	2 (4.9)	0	2	0	0	0	0
	Abdominal pain upper	1 (2.4)	0	0	1	0	0	0
	Anorectal ulcer	1 (2.4)	0	1	0	0	0	0
	Aphthous ulcer	2 (4.9)	0	0	2	0	0	0
	Constipation	2 (4.9)	0	2	0	0	0	0
	Diarrhoea	3 (7.3)	0	3	0	0	0	0
	Nausea	10 (24.4)	0	5	5	0	0	0
	Stomatitis	2 (4.9)	0	1	1	0	0	0
General disorders and administration site conditions	Patients with one or more Events	25 (61.0)	0	16	7	2	0	0
	Chest pain	1 (2.4)	0	0	1	0	0	0
	Fatigue	21 (51.2)	0	15	5	1	0	0
	Gait disturbance	2 (4.9)	0	1	1	0	0	0
	General physical health deterioration	3 (7.3)	0	0	3	0	0	0
	Mucosal inflammation	2 (4.9)	0	1	1	0	0	0
	Oedema peripheral	2 (4.9)	0	1	0	1	0	0
	Peripheral swelling	2 (4.9)	0	1	1	0	0	0
	Pyrexia	2 (4.9)	0	2	0	0	0	0
	Swelling face	2 (4.9)	0	2	0	0	0	0
Infections and infestations	Patients with one or more Events	19 (46.3)	1	6	8	1	1	2
	Bacterial infection	1 (2.4)	0	0	1	0	0	0
	COVID-19	4 (9.8)	0	3	0	0	0	1
	Candida infection	1 (2.4)	0	1	0	0	0	0
	Cystitis	2 (4.9)	0	0	2	0	0	0

The AE with the highest grading for each preferred term for each patient was taken. A patient who reports two or more Different preferred terms which are in the same system organ class, is counted only once in the system organ class total.
The highest grading of this patient in the respective system organ class will be taken.

Section 13 Table 6.1.2.2.1

Incidence and Severity of Adverse Events (All Causalities) - Full Analysis Set - Dosegroup: All Dosegroups

AEs that occured on or after the date of first medication and before end of observation have been considered as treament emergent AEs.

		N=41						
			Severity					
System organ class (MedDRA 23.0)	Preferred Term (MedDRA 23.0)	Number of patients (%) *	Missing	Grade 1	Grade 2	Grade 3	Grade 4	Grade 5
Infections and infestations	Gingivitis	1 (2.4)	0	1	0	0	0	0
	Influenza	1 (2.4)	0	1	0	0	0	0
	Nasopharyngitis	2 (4.9)	0	1	1	0	0	0
	Oral candidiasis	1 (2.4)	0	0	1	0	0	0
	Oral herpes	1 (2.4)	0	1	0	0	0	0
	Otitis externa	2 (4.9)	0	1	1	0	0	0
	Penile infection	1 (2.4)	0	1	0	0	0	0
	Pneumonia	3 (7.3)	0	0	0	1	1	1
	Rhinitis	1 (2.4)	0	0	1	0	0	0
	Sepsis	1 (2.4)	0	0	0	0	0	1
	Skin candida	1 (2.4)	0	1	0	0	0	0
	Upper respiratory tract infection	1 (2.4)	0	1	0	0	0	0
	Urinary tract infection	3 (7.3)	0	0	3	0	0	0
	Wound infection	1 (2.4)	1	0	0	0	0	0
Injury, poisoning and procedural complications	Patients with one or more Events	9 (22.0)	0	7	2	0	0	0
	Dislocation of vertebra	1 (2.4)	0	0	1	0	0	0
	Fall	2 (4.9)	0	2	0	0	0	0
	Ligament sprain	1 (2.4)	0	1	0	0	0	0
	Post procedural erythema	1 (2.4)	0	1	0	0	0	0
	Radiation alopecia	2 (4.9)	0	1	1	0	0	0
	Radiation skin injury	3 (7.3)	0	3	0	0	0	0

The AE with the highest grading for each preferred term for each patient was taken. A patient who reports two or more Different preferred terms which are in the same system organ class, is counted only once in the system organ class total.
The highest grading of this patient in the respective system organ class will be taken.

Section 13 Table 6.1.2.2.1

Incidence and Severity of Adverse Events (All Causalities) - Full Analysis Set - Dosegroup: All Dosegroups

AEs that occured on or after the date of first medication and before end of observation have been considered as treament emergent AEs.

		N=41						
			Severity					
System organ class (MedDRA 23.0)	Preferred Term (MedDRA 23.0)	Number of patients (%) *	Missing	Grade 1	Grade 2	Grade 3	Grade 4	Grade 5
Investigations	Patients with one or more Events	20 (48.8)	0	6	8	6	0	0
	Alanine aminotransferase increased	2 (4.9)	0	1	0	1	0	0
	Aspartate aminotransferase increased	1 (2.4)	0	0	1	0	0	0
	Blood creatine phosphokinase increased	1 (2.4)	0	1	0	0	0	0
	Blood creatinine increased	1 (2.4)	0	1	0	0	0	0
	Blood glucose increased	1 (2.4)	0	0	0	1	0	0
	Blood urea increased	1 (2.4)	0	1	0	0	0	0
	C-reactive protein increased	2 (4.9)	0	2	0	0	0	0
	Gamma-glutamyltransferase increased	1 (2.4)	0	0	0	1	0	0
	Glomerular filtration rate decreased	1 (2.4)	0	0	1	0	0	0
	Glycosylated haemoglobin increased	1 (2.4)	0	0	1	0	0	0
	Lymphocyte count decreased	3 (7.3)	0	1	1	1	0	0
	Neutrophil count decreased	4 (9.8)	0	1	3	0	0	0
	Platelet count decreased	3 (7.3)	0	1	2	0	0	0
	Weight decreased	2 (4.9)	0	1	0	1	0	0
	White blood cell count decreased	8 (19.5)	0	2	5	1	0	0
Metabolism and nutrition disorders	Patients with one or more Events	8 (19.5)	0	4	4	0	0	0
	Decreased appetite	5 (12.2)	0	3	2	0	0	0
	Hyperkalaemia	1 (2.4)	0	0	1	0	0	0
	Iron deficiency	1 (2.4)	0	1	0	0	0	0
	Vitamin B12 deficiency	1 (2.4)	0	0	1	0	0	0

The AE with the highest grading for each preferred term for each patient was taken. A patient who reports two or more Different preferred terms which are in the same system organ class, is counted only once in the system organ class total.
The highest grading of this patient in the respective system organ class will be taken.

Section 13 Table 6.1.2.2.1

Incidence and Severity of Adverse Events (All Causalities) - Full Analysis Set - Dosegroup: All Dosegroups

AEs that occured on or after the date of first medication and before end of observation have been considered as treament emergent AEs.

		N=41						
			Severity					
System organ class (MedDRA 23.0)	Preferred Term (MedDRA 23.0)	Number of patients (%) *	Missing	Grade 1	Grade 2	Grade 3	Grade 4	Grade 5
Musculoskeletal and connective tissue disorders	Patients with one or more Events	7 (17.1)	0	5	1	1	0	0
	Arthralgia	1 (2.4)	0	0	1	0	0	0
	Back pain	2 (4.9)	0	1	1	0	0	0
	Muscle spasms	1 (2.4)	0	1	0	0	0	0
	Muscle twitching	1 (2.4)	0	1	0	0	0	0
	Myalgia	1 (2.4)	0	1	0	0	0	0
	Neck pain	1 (2.4)	0	0	0	1	0	0
	Pain in extremity	1 (2.4)	0	1	0	0	0	0
	Tendon pain	1 (2.4)	0	1	0	0	0	0
Neoplasms benign, malignant and unspecified (incl cysts and polyps)	Patients with one or more Events	2 (4.9)	0	0	0	2	0	0
	Tumour pseudoprogression	2 (4.9)	0	0	0	2	0	0
Nervous system disorders	Patients with one or more Events	29 (70.7)	0	13	9	7	0	0
	Aphasia	7 (17.1)	0	2	4	1	0	0
	Apraxia	1 (2.4)	0	1	0	0	0	0
	Balance disorder	1 (2.4)	0	1	0	0	0	0
	Brain oedema	2 (4.9)	0	0	1	1	0	0
	Cerebral cyst	1 (2.4)	0	0	0	1	0	0
	Cerebral venous sinus thrombosis	1 (2.4)	0	0	0	1	0	0
	Cognitive disorder	1 (2.4)	0	0	1	0	0	0
	Coordination abnormal	1 (2.4)	0	1	0	0	0	0
	Disturbance in attention	3 (7.3)	0	0	3	0	0	0
	Dizziness	2 (4.9)	0	0	2	0	0	0
	Dysaesthesia	1 (2.4)	0	1	0	0	0	0
	Dysgeusia	3 (7.3)	0	3	0	0	0	0
	Epilepsy	1 (2.4)	0	1	0	0	0	0
	Fine motor skill dysfunction	1 (2.4)	0	0	1	0	0	0

The AE with the highest grading for each preferred term for each patient was taken. A patient who reports two or more Different preferred terms which are in the same system organ class, is counted only once in the system organ class total.
The highest grading of this patient in the respective system organ class will be taken.

Section 13 Table 6.1.2.2.1
Incidence and Severity of Adverse Events (All Causalities) - Full Analysis Set - Dosegroup: All Dosegroups

AEs that occured on or after the date of first medication and before end of observation have been considered as treament emergent AEs.

		N=41						
			Severity					
System organ class (MedDRA 23.0)	Preferred Term (MedDRA 23.0)	Number of patients (%) *	Missing	Grade 1	Grade 2	Grade 3	Grade 4	Grade 5
Nervous system disorders	Headache	9 (22.0)	0	6	3	0	0	0
	Hemiparesis	4 (9.8)	0	0	4	0	0	0
	Hyperaesthesia	1 (2.4)	0	1	0	0	0	0
	Hypoaesthesia	2 (4.9)	0	2	0	0	0	0
	Hypotonic-hyporesponsive episode	1 (2.4)	0	0	1	0	0	0
	Memory impairment	2 (4.9)	0	2	0	0	0	0
	Monoparesis	1 (2.4)	0	1	0	0	0	0
	Neurological decompensation	1 (2.4)	0	0	0	1	0	0
	Orthostatic intolerance	1 (2.4)	0	0	1	0	0	0
	Partial seizures	2 (4.9)	0	1	1	0	0	0
	Presyncope	1 (2.4)	0	1	0	0	0	0
	Reflexes abnormal	1 (2.4)	0	1	0	0	0	0
	Seizure	6 (14.6)	0	2	4	0	0	0
	Status epilepticus	1 (2.4)	0	0	0	1	0	0
	Syncope	1 (2.4)	0	0	0	1	0	0
	Visual pathway disorder	1 (2.4)	0	0	1	0	0	0
Psychiatric disorders	Patients with one or more Events	9 (22.0)	0	4	3	2	0	0
	Anxiety	2 (4.9)	0	0	2	0	0	0
	Confusional state	1 (2.4)	0	0	1	0	0	0
	Depressed mood	1 (2.4)	0	1	0	0	0	0
	Depression	2 (4.9)	0	0	1	1	0	0
	Hypomania	1 (2.4)	0	0	0	1	0	0
	Insomnia	2 (4.9)	0	1	1	0	0	0
	Organic brain syndrome	1 (2.4)	0	0	0	1	0	0
	Restlessness	1 (2.4)	0	1	0	0	0	0
	Sleep disorder	2 (4.9)	0	2	0	0	0	0

The AE with the highest grading for each preferred term for each patient was taken. A patient who reports two or more Different preferred terms which are in the same system organ class, is counted only once in the system organ class total.
The highest grading of this patient in the respective system organ class will be taken.

Section 13 Table 6.1.2.2.1
Incidence and Severity of Adverse Events (All Causalities) - Full Analysis Set - Dosegroup: All Dosegroups

AEs that occured on or after the date of first medication and before end of observation have been considered as treament emergent AEs.

		N=41						
			Severity					
**System organ class (MedDRA 23.0)**	**Preferred Term (MedDRA 23.0)**	**Number of patients (%) ***	**Missing**	**Grade 1**	**Grade 2**	**Grade 3**	**Grade 4**	**Grade 5**
Renal and urinary disorders	Patients with one or more Events	4 (9.8)	0	3	1	0	0	0
	Glycosuria	1 (2.4)	0	1	0	0	0	0
	Haematuria	1 (2.4)	0	1	0	0	0	0
	Incontinence	1 (2.4)	0	1	0	0	0	0
	Renal failure	1 (2.4)	0	0	1	0	0	0
Respiratory, thoracic and mediastinal disorders	Patients with one or more Events	7 (17.1)	0	3	2	1	1	0
	Cough	3 (7.3)	0	1	2	0	0	0
	Dyspnoea	2 (4.9)	0	1	0	1	0	0
	Productive cough	1 (2.4)	0	1	0	0	0	0
	Pulmonary embolism	2 (4.9)	0	0	0	1	1	0
Skin and subcutaneous tissue disorders	Patients with one or more Events	25 (61.0)	0	13	12	0	0	0
	Alopecia	22 (53.7)	0	11	11	0	0	0
	Dry skin	1 (2.4)	0	1	0	0	0	0
	Erythema	2 (4.9)	0	2	0	0	0	0
	Hyperhidrosis	1 (2.4)	0	1	0	0	0	0
	Night sweats	1 (2.4)	0	0	1	0	0	0
	Photosensitivity reaction	1 (2.4)	0	0	1	0	0	0
	Rash	2 (4.9)	0	2	0	0	0	0
	Rash pruritic	1 (2.4)	0	0	1	0	0	0
	Rosacea	1 (2.4)	0	1	0	0	0	0
	Skin hyperpigmentation	1 (2.4)	0	0	1	0	0	0
Surgical and medical procedures	Patients with one or more Events	1 (2.4)	0	1	0	0	0	0
	Cardiac pacemaker insertion	1 (2.4)	0	1	0	0	0	0

The AE with the highest grading for each preferred term for each patient was taken. A patient who reports two or more Different preferred terms which are in the same system organ class, is counted only once in the system organ class total.
The highest grading of this patient in the respective system organ class will be taken.

Section 13 Table 6.1.2.2.1

Incidence and Severity of Adverse Events (All Causalities) - Full Analysis Set - Dosegroup: All Dosegroups

AEs that occured on or after the date of first medication and before end of observation have been considered as treament emergent AEs.

		N=41						
			Severity					
System organ class (MedDRA 23.0)	Preferred Term (MedDRA 23.0)	Number of patients (%) *	Missing	Grade 1	Grade 2	Grade 3	Grade 4	Grade 5
Vascular disorders	Patients with one or more Events	3 (7.3)	0	1	2	0	0	0
	Paraneoplastic thrombosis	1 (2.4)	0	0	1	0	0	0
	Post thrombotic syndrome	1 (2.4)	0	1	0	0	0	0
	Thrombophlebitis	1 (2.4)	0	0	1	0	0	0
	Thrombosis	1 (2.4)	0	1	0	0	0	0

The AE with the highest grading for each preferred term for each patient was taken. A patient who reports two or more Different preferred terms which are in the same system organ class, is counted only once in the system organ class total.
The highest grading of this patient in the respective system organ class will be taken.

Incidence and Severity of Adverse Events (All Causalities) - Full Analysis Set

AEs that occured on or after the date of first medication and before end of observation have been considered as treament emergent AEs.

		N=46						
			Severity					
System organ class (MedDRA 23.0)	Preferred Term (MedDRA 23.0)	Number of patients (%) *	Missing	Grade 1	Grade 2	Grade 3	Grade 4	Grade 5
Blood and lymphatic system disorders	Patients with one or more Events	21 (45.7)	0	5	11	3	2	0
	Anaemia	6 (13.0)	0	1	4	1	0	0
	Leukopenia	6 (13.0)	0	1	5	0	0	0
	Lymphopenia	11 (23.9)	0	1	8	1	1	0
	Neutropenia	2 (4.3)	0	1	0	1	0	0
	Thrombocytopenia	6 (13.0)	0	4	1	0	1	0
Cardiac disorders	Patients with one or more Events	1 (2.2)	0	1	0	0	0	0
	Atrioventricular block first degree	1 (2.2)	0	1	0	0	0	0
Ear and labyrinth disorders	Patients with one or more Events	8 (17.4)	0	6	1	1	0	0
	Auricular swelling	1 (2.2)	0	0	1	0	0	0
	Ear discomfort	1 (2.2)	0	1	0	0	0	0
	Hypoacusis	1 (2.2)	0	1	0	0	0	0
	Sudden hearing loss	1 (2.2)	0	0	0	1	0	0
	Tinnitus	2 (4.3)	0	2	0	0	0	0
	Vertigo	2 (4.3)	0	2	0	0	0	0
Eye disorders	Patients with one or more Events	5 (10.9)	0	3	2	0	0	0
	Dry eye	1 (2.2)	0	1	0	0	0	0
	Eye disorder	1 (2.2)	0	1	0	0	0	0
	Eyelid oedema	1 (2.2)	0	1	0	0	0	0
	Ocular discomfort	1 (2.2)	0	1	0	0	0	0
	Retinal detachment	1 (2.2)	0	0	1	0	0	0
	Visual impairment	1 (2.2)	0	0	1	0	0	0
Gastrointestinal disorders	Patients with one or more Events	27 (58.7)	0	11	13	3	0	0
	Abdominal pain	1 (2.2)	0	1	0	0	0	0
	Abdominal pain lower	1 (2.2)	0	1	0	0	0	0
	Abdominal pain upper	2 (4.3)	0	0	2	0	0	0
	Abdominal tenderness	1 (2.2)	0	1	0	0	0	0

The AE with the highest grading for each preferred term for each patient was taken. A patient who reports two or more Different preferred terms which are in the same system organ class, is counted only once in the system organ class total.
The highest grading of this patient in the respective system organ class will be taken.

Incidence and Severity of Adverse Events (All Causalities) - Full Analysis Set

AEs that occured on or after the date of first medication and before end of observation have been considered as treament emergent AEs.

		N=46						
			Severity					
System organ class (MedDRA 23.0)	Preferred Term (MedDRA 23.0)	Number of patients (%) *	Missing	Grade 1	Grade 2	Grade 3	Grade 4	Grade 5
Gastrointestinal disorders	Anal eczema	1 (2.2)	0	0	1	0	0	0
	Anal fissure	1 (2.2)	0	0	1	0	0	0
	Aphthous ulcer	3 (6.5)	0	0	2	1	0	0
	Constipation	3 (6.5)	0	2	1	0	0	0
	Diarrhoea	7 (15.2)	0	4	3	0	0	0
	Dry mouth	1 (2.2)	0	1	0	0	0	0
	Dysphagia	2 (4.3)	0	2	0	0	0	0
	Gastritis	1 (2.2)	0	1	0	0	0	0
	Gastrooesophageal reflux disease	1 (2.2)	0	0	1	0	0	0
	Haemorrhoids	2 (4.3)	0	1	1	0	0	0
	Irritable bowel syndrome	1 (2.2)	0	0	1	0	0	0
	Nausea	6 (13.0)	0	1	5	0	0	0
	Oral discomfort	1 (2.2)	0	1	0	0	0	0
	Oral pain	2 (4.3)	0	0	0	2	0	0
	Plicated tongue	1 (2.2)	0	0	0	1	0	0
	Proctalgia	1 (2.2)	0	1	0	0	0	0
	Stomatitis	11 (23.9)	0	6	3	2	0	0
	Toothache	2 (4.3)	0	1	1	0	0	0
	Vomiting	1 (2.2)	0	0	1	0	0	0
General disorders and administration site conditions	Patients with one or more Events	27 (58.7)	0	12	12	3	0	0
	Chills	1 (2.2)	0	1	0	0	0	0
	Face oedema	3 (6.5)	0	3	0	0	0	0
	Facial pain	1 (2.2)	0	1	0	0	0	0
	Fatigue	14 (30.4)	0	11	3	0	0	0
	Gait disturbance	5 (10.9)	0	2	2	1	0	0
	General physical health deterioration	3 (6.5)	0	0	2	1	0	0

The AE with the highest grading for each preferred term for each patient was taken. A patient who reports two or more Different preferred terms which are in the same system organ class, is counted only once in the system organ class total.
The highest grading of this patient in the respective system organ class will be taken.

Incidence and Severity of Adverse Events (All Causalities) - Full Analysis Set

AEs that occured on or after the date of first medication and before end of observation have been considered as treament emergent AEs.

		N=46						
			Severity					
System organ class (MedDRA 23.0)	Preferred Term (MedDRA 23.0)	Number of patients (%) *	Missing	Grade 1	Grade 2	Grade 3	Grade 4	Grade 5
General disorders and administration site conditions	Impaired healing	1 (2.2)	0	1	0	0	0	0
	Mucosal disorder	4 (8.7)	0	2	2	0	0	0
	Mucosal inflammation	6 (13.0)	0	1	5	0	0	0
	Oedema	1 (2.2)	0	1	0	0	0	0
	Oedema peripheral	3 (6.5)	0	2	1	0	0	0
	Peripheral swelling	1 (2.2)	0	1	0	0	0	0
	Pyrexia	3 (6.5)	0	1	0	2	0	0
Hepatobiliary disorders	Patients with one or more Events	2 (4.3)	0	0	0	2	0	0
	Hepatotoxicity	2 (4.3)	0	0	0	2	0	0
Immune system disorders	Patients with one or more Events	2 (4.3)	0	0	2	0	0	0
	Allergic reaction to excipient	1 (2.2)	0	0	1	0	0	0
	Drug hypersensitivity	1 (2.2)	0	0	1	0	0	0
	Hypersensitivity	1 (2.2)	0	0	1	0	0	0
Infections and infestations	Patients with one or more Events	32 (69.6)	0	6	12	14	0	0
	Asymptomatic bacteriuria	1 (2.2)	0	1	0	0	0	0
	Atypical pneumonia	2 (4.3)	0	0	1	1	0	0
	Bronchitis	1 (2.2)	0	0	1	0	0	0
	Candida infection	1 (2.2)	0	0	1	0	0	0
	Conjunctivitis	2 (4.3)	0	2	0	0	0	0
	Cystitis	2 (4.3)	0	0	2	0	0	0
	Folliculitis	5 (10.9)	0	2	1	2	0	0
	Gingivitis	1 (2.2)	0	1	0	0	0	0
	Herpes simplex reactivation	1 (2.2)	0	0	1	0	0	0
	Herpes zoster	1 (2.2)	0	0	0	1	0	0
	Infection	6 (13.0)	0	1	3	2	0	0
	Lip infection	1 (2.2)	0	1	0	0	0	0
	Localised infection	1 (2.2)	0	1	0	0	0	0

The AE with the highest grading for each preferred term for each patient was taken. A patient who reports two or more Different preferred terms which are in the same system organ class, is counted only once in the system organ class total.
The highest grading of this patient in the respective system organ class will be taken.

Section 13 Table 6.1.2.1

Incidence and Severity of Adverse Events (All Causalities) - Full Analysis Set

AEs that occured on or after the date of first medication and before end of observation have been considered as treament emergent AEs.

		N=46						
			Severity					
System organ class (MedDRA 23.0)	Preferred Term (MedDRA 23.0)	Number of patients (%) *	Missing	Grade 1	Grade 2	Grade 3	Grade 4	Grade 5
Infections and infestations	Nail infection	1 (2.2)	0	0	1	0	0	0
	Nasopharyngitis	3 (6.5)	0	3	0	0	0	0
	Oral fungal infection	1 (2.2)	0	0	1	0	0	0
	Oral herpes	4 (8.7)	0	2	2	0	0	0
	Otitis externa	1 (2.2)	0	0	1	0	0	0
	Paronychia	2 (4.3)	0	0	2	0	0	0
	Pharyngitis	1 (2.2)	0	1	0	0	0	0
	Pneumocystis jirovecii pneumonia	1 (2.2)	0	0	0	1	0	0
	Pneumonia	7 (15.2)	0	2	3	2	0	0
	Postoperative wound infection	1 (2.2)	0	0	0	1	0	0
	Pulpitis dental	1 (2.2)	0	1	0	0	0	0
	Sinusitis	1 (2.2)	0	0	1	0	0	0
	Staphylococcal infection	1 (2.2)	0	0	0	1	0	0
	Upper respiratory tract infection	2 (4.3)	0	0	1	1	0	0
	Urinary tract infection	3 (6.5)	0	0	3	0	0	0
	Viral infection	1 (2.2)	0	0	1	0	0	0
	Wound infection	2 (4.3)	0	0	0	2	0	0
Injury, poisoning and procedural complications	Patients with one or more Events	9 (19.6)	0	6	3	0	0	0
	Ear injury	1 (2.2)	0	1	0	0	0	0
	Fall	2 (4.3)	0	1	1	0	0	0
	Postoperative wound complication	1 (2.2)	0	0	1	0	0	0
	Radiation alopecia	1 (2.2)	0	1	0	0	0	0
	Radiation skin injury	3 (6.5)	0	2	1	0	0	0
	Spinal fracture	1 (2.2)	0	1	0	0	0	0

The AE with the highest grading for each preferred term for each patient was taken. A patient who reports two or more Different preferred terms which are in the same system organ class, is counted only once in the system organ class total.
The highest grading of this patient in the respective system organ class will be taken.

Section 13 Table 6.1.2.1
Incidence and Severity of Adverse Events (All Causalities) - Full Analysis Set

AEs that occured on or after the date of first medication and before end of observation have been considered as treament emergent AEs.

		N=46						
			Severity					
System organ class (MedDRA 23.0)	Preferred Term (MedDRA 23.0)	Number of patients (%) *	Missing	Grade 1	Grade 2	Grade 3	Grade 4	Grade 5
Investigations	Patients with one or more Events	22 (47.8)	0	6	11	5	0	0
	Alanine aminotransferase increased	3 (6.5)	0	3	0	0	0	0
	Aspartate aminotransferase increased	3 (6.5)	0	3	0	0	0	0
	Blood alkaline phosphatase increased	2 (4.3)	0	2	0	0	0	0
	Blood creatine phosphokinase increased	2 (4.3)	0	2	0	0	0	0
	Blood lactate dehydrogenase increased	2 (4.3)	0	2	0	0	0	0
	C-reactive protein increased	6 (13.0)	0	1	2	3	0	0
	Gamma-glutamyltransferase increased	2 (4.3)	0	0	2	0	0	0
	Haemoglobin increased	1 (2.2)	0	0	1	0	0	0
	Liver function test increased	1 (2.2)	0	0	1	0	0	0
	Lymphocyte count decreased	6 (13.0)	0	0	5	1	0	0
	Neutrophil count decreased	2 (4.3)	0	0	1	1	0	0
	Platelet count decreased	1 (2.2)	0	1	0	0	0	0
	Serum ferritin decreased	1 (2.2)	0	1	0	0	0	0
	Transaminases increased	1 (2.2)	0	0	1	0	0	0
	Troponin increased	1 (2.2)	0	0	1	0	0	0
	Weight decreased	6 (13.0)	0	6	0	0	0	0
	White blood cell count decreased	2 (4.3)	0	0	2	0	0	0
Metabolism and nutrition disorders	Patients with one or more Events	16 (34.8)	0	9	4	3	0	0
	Decreased appetite	5 (10.9)	0	5	0	0	0	0
	Dehydration	1 (2.2)	0	0	1	0	0	0
	Diabetes mellitus	1 (2.2)	0	0	1	0	0	0
	Hyperglycaemia	2 (4.3)	0	1	0	1	0	0
	Hypokalaemia	5 (10.9)	0	3	2	0	0	0

The AE with the highest grading for each preferred term for each patient was taken. A patient who reports two or more Different preferred terms which are in the same system organ class, is counted only once in the system organ class total.
The highest grading of this patient in the respective system organ class will be taken.

Incidence and Severity of Adverse Events (All Causalities) - Full Analysis Set
AEs that occured on or after the date of first medication and before end of observation have been considered as treament emergent AEs.

		N=46						
			Severity					
System organ class (MedDRA 23.0)	Preferred Term (MedDRA 23.0)	Number of patients (%) *	Missing	Grade 1	Grade 2	Grade 3	Grade 4	Grade 5
Metabolism and nutrition disorders	Hyponatraemia	2 (4.3)	0	0	1	1	0	0
	Hypophosphataemia	2 (4.3)	0	0	1	1	0	0
	Iron deficiency	1 (2.2)	0	1	0	0	0	0
	Type 2 diabetes mellitus	1 (2.2)	0	0	1	0	0	0
	Vitamin D deficiency	1 (2.2)	0	1	0	0	0	0
Musculoskeletal and connective tissue disorders	Patients with one or more Events	6 (13.0)	0	4	2	0	0	0
	Back pain	3 (6.5)	0	1	2	0	0	0
	Muscle spasms	1 (2.2)	0	1	0	0	0	0
	Musculoskeletal pain	1 (2.2)	0	1	0	0	0	0
	Osteoporosis	1 (2.2)	0	0	1	0	0	0
	Pain in extremity	1 (2.2)	0	1	0	0	0	0
Nervous system disorders	Patients with one or more Events	29 (63.0)	0	11	12	4	1	1
	Aphasia	6 (13.0)	0	1	3	2	0	0
	Ataxia	1 (2.2)	0	1	0	0	0	0
	Brain oedema	1 (2.2)	0	1	0	0	0	0
	Burning sensation	2 (4.3)	0	1	1	0	0	0
	Cerebral haemorrhage	1 (2.2)	0	0	0	0	0	1
	Cognitive disorder	1 (2.2)	0	0	1	0	0	0
	Disturbance in attention	2 (4.3)	0	2	0	0	0	0
	Dizziness	2 (4.3)	0	1	1	0	0	0
	Dysarthria	1 (2.2)	0	0	1	0	0	0
	Dysgeusia	5 (10.9)	0	5	0	0	0	0
	Epilepsy	1 (2.2)	0	0	0	1	0	0
	Facial paresis	1 (2.2)	0	1	0	0	0	0
	Guillain-Barre syndrome	1 (2.2)	0	0	0	0	1	0
	Head discomfort	1 (2.2)	0	1	0	0	0	0
	Headache	8 (17.4)	0	3	5	0	0	0

The AE with the highest grading for each preferred term for each patient was taken. A patient who reports two or more Different preferred terms which are in the same system organ class, is counted only once in the system organ class total.
The highest grading of this patient in the respective system organ class will be taken.

Incidence and Severity of Adverse Events (All Causalities) - Full Analysis Set

AEs that occured on or after the date of first medication and before end of observation have been considered as treament emergent AEs.

		N=46						
			Severity					
**System organ class (MedDRA 23.0)**	**Preferred Term (MedDRA 23.0)**	**Number of patients (%) ***	**Missing**	**Grade 1**	**Grade 2**	**Grade 3**	**Grade 4**	**Grade 5**
Nervous system disorders	Hemianopia	2 (4.3)	0	2	0	0	0	0
	Hemiparesis	7 (15.2)	0	2	4	1	0	0
	Hydrocephalus	1 (2.2)	0	0	0	1	0	0
	Hypoaesthesia	3 (6.5)	0	3	0	0	0	0
	Hyporeflexia	1 (2.2)	0	1	0	0	0	0
	Memory impairment	1 (2.2)	0	0	1	0	0	0
	Monoparesis	1 (2.2)	0	1	0	0	0	0
	Neurologic neglect syndrome	1 (2.2)	0	0	1	0	0	0
	Partial seizures	3 (6.5)	0	1	2	0	0	0
	Psychomotor hyperactivity	1 (2.2)	0	1	0	0	0	0
	Seizure	6 (13.0)	0	2	2	2	0	0
	Syncope	1 (2.2)	0	0	0	1	0	0
	Taste disorder	4 (8.7)	0	3	1	0	0	0
	Tremor	1 (2.2)	0	1	0	0	0	0
	VIth nerve disorder	1 (2.2)	0	0	1	0	0	0
	Visual field defect	1 (2.2)	0	0	1	0	0	0
Psychiatric disorders	Patients with one or more Events	11 (23.9)	0	6	5	0	0	0
	Depression	4 (8.7)	0	1	3	0	0	0
	Insomnia	3 (6.5)	0	2	1	0	0	0
	Psychomotor retardation	1 (2.2)	0	1	0	0	0	0
	Sleep disorder	3 (6.5)	0	2	1	0	0	0
Renal and urinary disorders	Patients with one or more Events	2 (4.3)	0	0	2	0	0	0
	Glycosuria	1 (2.2)	0	0	1	0	0	0
	Micturition urgency	1 (2.2)	0	0	1	0	0	0
	Pollakiuria	1 (2.2)	0	1	0	0	0	0

The AE with the highest grading for each preferred term for each patient was taken. A patient who reports two or more Different preferred terms which are in the same system organ class, is counted only once in the system organ class total.

The highest grading of this patient in the respective system organ class will be taken.

Incidence and Severity of Adverse Events (All Causalities) - Full Analysis Set

AEs that occured on or after the date of first medication and before end of observation have been considered as treament emergent AEs.

		N=46						
			Severity					
System organ class (MedDRA 23.0)	Preferred Term (MedDRA 23.0)	Number of patients (%) *	Missing	Grade 1	Grade 2	Grade 3	Grade 4	Grade 5
Reproductive system and breast disorders	Patients with one or more Events	1 (2.2)	0	1	0	0	0	0
	Vulvovaginal burning sensation	1 (2.2)	0	1	0	0	0	0
Respiratory, thoracic and mediastinal disorders	Patients with one or more Events	13 (28.3)	0	8	5	0	0	0
	Cough	2 (4.3)	0	1	1	0	0	0
	Dysphonia	1 (2.2)	0	1	0	0	0	0
	Dyspnoea	2 (4.3)	0	1	1	0	0	0
	Epistaxis	2 (4.3)	0	2	0	0	0	0
	Hiccups	1 (2.2)	0	1	0	0	0	0
	Oropharyngeal pain	3 (6.5)	0	3	0	0	0	0
	Pneumonia aspiration	1 (2.2)	0	0	1	0	0	0
	Pneumonitis	1 (2.2)	0	0	1	0	0	0
	Pulmonary embolism	1 (2.2)	0	0	1	0	0	0
	Respiratory failure	1 (2.2)	0	0	1	0	0	0
Skin and subcutaneous tissue disorders	Patients with one or more Events	32 (69.6)	0	16	16	0	0	0
	Acne	5 (10.9)	0	2	3	0	0	0
	Alopecia	12 (26.1)	0	9	3	0	0	0
	Cold sweat	1 (2.2)	0	0	1	0	0	0
	Dermatitis acneiform	4 (8.7)	0	2	2	0	0	0
	Dermatitis allergic	1 (2.2)	0	1	0	0	0	0
	Drug eruption	1 (2.2)	0	0	1	0	0	0
	Dry skin	3 (6.5)	0	1	2	0	0	0
	Eczema	2 (4.3)	0	1	1	0	0	0
	Erythema	2 (4.3)	0	2	0	0	0	0
	Nail discolouration	1 (2.2)	0	1	0	0	0	0
	Night sweats	1 (2.2)	0	1	0	0	0	0
	Pain of skin	1 (2.2)	0	0	1	0	0	0

The AE with the highest grading for each preferred term for each patient was taken. A patient who reports two or more Different preferred terms which are in the same system organ class, is counted only once in the system organ class total.
The highest grading of this patient in the respective system organ class will be taken.

Section 13 Table 6.1.2.1

Incidence and Severity of Adverse Events (All Causalities) - Full Analysis Set

AEs that occured on or after the date of first medication and before end of observation have been considered as treament emergent AEs.

		N=46						
			Severity					
System organ class (MedDRA 23.0)	Preferred Term (MedDRA 23.0)	Number of patients (%) *	Missing	Grade 1	Grade 2	Grade 3	Grade 4	Grade 5
Skin and subcutaneous tissue disorders	Photosensitivity reaction	1 (2.2)	0	1	0	0	0	0
	Pruritus	5 (10.9)	0	4	1	0	0	0
	Rash	10 (21.7)	0	5	5	0	0	0
	Rash maculo-papular	1 (2.2)	0	0	1	0	0	0
	Rash papular	1 (2.2)	0	0	1	0	0	0
	Scar pain	1 (2.2)	0	1	0	0	0	0
	Skin fissures	1 (2.2)	0	0	1	0	0	0
	Skin irritation	3 (6.5)	0	1	2	0	0	0
	Skin ulcer	2 (4.3)	0	2	0	0	0	0
Surgical and medical procedures	Patients with one or more Events	1 (2.2)	0	0	1	0	0	0
	Endodontic procedure	1 (2.2)	0	0	1	0	0	0
Vascular disorders	Patients with one or more Events	11 (23.9)	0	4	5	2	0	0
	Embolism	1 (2.2)	0	0	1	0	0	0
	Haematoma	1 (2.2)	0	1	0	0	0	0
	Hypertension	4 (8.7)	0	1	2	1	0	0
	Hypertensive crisis	1 (2.2)	0	0	1	0	0	0
	Hypotension	1 (2.2)	0	1	0	0	0	0
	Thrombophlebitis	1 (2.2)	0	1	0	0	0	0
	Thrombosis	2 (4.3)	0	0	1	1	0	0

The AE with the highest grading for each preferred term for each patient was taken. A patient who reports two or more Different preferred terms which are in the same system organ class, is counted only once in the system organ class total.
The highest grading of this patient in the respective system organ class will be taken.
