# Supplementary material for: Molecularly matched targeted therapies plus radiotherapy in glioblastoma: the phase 1/2a N2M2 umbrella trial
Source: Nat Med. 2025 Sep 5;31(10):3534–41. doi: 10.1038/s41591-025-03928-9 (PMC12532562; doi:10.1038/s41591-025-03928-9)
Supplement: Supplementary file 2 — Reporting Summary [file 41591_2025_3928_MOESM2_ESM.pdf]

nature portfolio

Corresponding author(s): Wolfgang Wick

Last updated by author(s): Feb 19, 2025

Reporting Summary

Nature Portfolio wishes to improve the reproducibility of the work that we publish. This form provides structure for consistency and transparency in reporting. For further information on Nature Portfolio policies, see our Editorial Policies and the Editorial Policy Checklist.

Statistics

For all statistical analyses, confirm that the following items are present in the figure legend, table legend, main text, or Methods section.

n/a	Confirmed	
☐	☒	The exact sample size (*n*) for each experimental group/condition, given as a discrete number and unit of measurement
☐	☒	A statement on whether measurements were taken from distinct samples or whether the same sample was measured repeatedly
☐	☒	The statistical test(s) used AND whether they are one- or two-sided<br>*Only common tests should be described solely by name; describe more complex techniques in the Methods section.*
☐	☒	A description of all covariates tested
☐	☒	A description of any assumptions or corrections, such as tests of normality and adjustment for multiple comparisons
☐	☒	A full description of the statistical parameters including central tendency (e.g. means) or other basic estimates (e.g. regression coefficient) AND variation (e.g. standard deviation) or associated estimates of uncertainty (e.g. confidence intervals)
☐	☒	For null hypothesis testing, the test statistic (e.g. *F*, *t*, *r*) with confidence intervals, effect sizes, degrees of freedom and *P* value noted<br>*Give P values as exact values whenever suitable.*
☒	☐	For Bayesian analysis, information on the choice of priors and Markov chain Monte Carlo settings
☒	☐	For hierarchical and complex designs, identification of the appropriate level for tests and full reporting of outcomes
☒	☐	Estimates of effect sizes (e.g. Cohen's *d*, Pearson's *r*), indicating how they were calculated

Our web collection on statistics for biologists contains articles on many of the points above.

Software and code

Policy information about availability of computer code

Data collection	Clincase Version 2.7 (EDC System)
Data analysis	SAS Version 9.4 (Software used for statistical analysis)

For manuscripts utilizing custom algorithms or software that are central to the research but not yet described in published literature, software must be made available to editors and reviewers. We strongly encourage code deposition in a community repository (e.g. GitHub). See the Nature Portfolio guidelines for submitting code & software for further information.

Data

Policy information about availability of data

All manuscripts must include a data availability statement. This statement should provide the following information, where applicable:

- Accession codes, unique identifiers, or web links for publicly available datasets
- A description of any restrictions on data availability
- For clinical datasets or third party data, please ensure that the statement adheres to our policy

These are patient outcomes data from the clinical study. These will be aasonable request to the corresponding author from the N2M2 study CRFs.
Molecular raw data that support the findings of this study in Fig. 2, 3, ED Fig. 3, 4 as well as ED Table 1-4 are available from the corresponding author upon reasonable request.
Sequence and methylation data have been deposited at the European Genomephenome Archive (EGA), which is hosted by the European Bioinformatics Institute

and the Centre for Genomic Regulation under accession number EGAS00001008033 (https://ega-archive.org; RRID: SCR_004944).

Raw data for the sequencing and methylation analyses have been deposited at GEO xyyxyx.

Research involving human participants, their data, or biological material

Policy information about studies with human participants or human data. See also policy information about sex, gender (identity/presentation), and sexual orientation and race, ethnicity and racism.

Reporting on sex and gender	The trial reports the sex distribution, but does not have had access to gender information.
Reporting on race, ethnicity, or other socially relevant groupings	The trial has not captured social groupings and reports ethnic groups in ED Table 1.
Population characteristics	These data are integral for the N2M2 trial as such and are reported in detail in Fig. 2, ED Table 1 and 3
Recruitment	All trial sites screened all patients with the principal diagnosis of a newly glioblastoma and kept a screening list. Principally eligible patients have then been consented to the molecular analyses. Patients have been enrolled in 13 Neurooncology Working Group of the German Cancer Society (NOA) trial sites in Germany. Based on molecular findings ("match" / "no match") patients have been allocated in seven different subtrials or the control group.<br>For the "match" / "no match" decision fresh tumor tissue and blood from glioblastoma patients with an non-hypermethylated MGMT promoter was widely examined by neuropathological analysis. Results were available within a maximum of 3 weeks postoperatively allowing a dedicated bioinformatics evaluation which forms the basis for the final treatment decision by the MTB and afterwards a timely initiation (≤6 weeks) of postoperative treatments. The workflow and timelines of molecular diagnostics and treatment decisions have been summarized in the published study protocol18.
Ethics oversight	The trial was conducted in accordance with the standards of Good Clinical Practice, the Declaration of Helsinki and local legal and regulatory requirements. The study protocol has been approved by the lead Ethics Committee (AFmu-207/2017) in Heidelberg and all regional ethics committees as well as the competent federal authority (Vorlagennummer 3051/01, Paul-Ehrlich-Institute in Langen, Germany).<br>For this trial, the EudraCT number 2015-002752-27 has been obtained. The trial has been registered at NCT03158389 Monitoring and pharmacovigilance was performed by the Coordination Center for Clinical Trials (KKS) Heidelberg.

Note that full information on the approval of the study protocol must also be provided in the manuscript.

Field-specific reporting

Please select the one below that is the best fit for your research. If you are not sure, read the appropriate sections before making your selection.

☒ Life sciences ☐ Behavioural & social sciences ☐ Ecological, evolutionary & environmental sciences

For a reference copy of the document with all sections, see nature.com/documents/nr-reporting-summary-flat.pdf

Life sciences study design

All studies must disclose on these points even when the disclosure is negative.

Sample size	In the phase I parts, patients were enrolled depending on observed toxicities. In the phase IIa parts a maximum of 40 patients in each subtrial were to be accrued for evaluation, wherein 6 to 9 patients of the according dose of an eventual phase I part were included. The exact number depends on early stopping for toxicity or futility or overshooting in cases of rapid enrolment. The "non-matching" group was anticipated to include approximately 35% of all screened study patients. Therefore, 12% of all screened patients were expected to be enrolled in the control group receiving TMZ.
Data exclusions	All treated patients were analyzed. Patients who received the final dose were part of the Full Analysis Set for Phase IIa, all other patients were analyzed for Phase I. Patients without treatment are not further analyzed.
Replication	Raw data, derived datasets and programs used for statistical analysis at the NCT study center are stored permanently and are accessible on demand. The SAS software is permanently available for reuse.
Randomization	Stratification for treatment was performed in a trial-specific molecular tumor board in five subtrials, including alectinib, idasanutlin, palbociclib, vismodegib and temsirolimus as targeted therapies (details on the molecules are provided in the Supplement), according to the best matching molecular alteration. Patients without matching alterations have been randomized between subtrials without strong biomarkers using atezolizumab and asunercept (APG101), and the standard of care (SOC), TMZ using a web based tool (www.randomizer.at).
Blinding	Because of the principal differences in the applications of the different treatments in the 8 trial arms blinding was not manageble.

Reporting for specific materials, systems and methods

We require information from authors about some types of materials, experimental systems and methods used in many studies. Here, indicate whether each material, system or method listed is relevant to your study. If you are not sure if a list item applies to your research, read the appropriate section before selecting a response.

Materials & experimental systems

n/a	Involved in the study
☒	☐ Antibodies
☒	☐ Eukaryotic cell lines
☒	☐ Palaeontology and archaeology
☒	☐ Animals and other organisms
☐	☒ Clinical data
☒	☐ Dual use research of concern
☒	☐ Plants

Methods

n/a	Involved in the study
☒	☐ ChIP-seq
☒	☐ Flow cytometry
☐	☒ MRI-based neuroimaging

Clinical data

Policy information about clinical studies

All manuscripts should comply with the ICMJE guidelines for publication of clinical research and a completed CONSORT checklist must be included with all submissions.

Clinical trial registration	EudraCT number 2015-002752-27 and NCT03158389
Study protocol	The protocol and the subprotocols for the different trial arms are submitted and available foree review.
Data collection	From May 2018 through July 2022, 301 patients were enrolled, 249 allocated to treatments and 228 treated. data collection ended

Outcomes

Assessments of safety and efficacy

All adverse events that occurred during the trial after the first experimental treatment have been recorded, graded according to the Common Terminology Criteria for Adverse Events [CTCAE] Version 5.0 at every study visit and followed-up until resolution or stabilization. Safety endpoints were assessed by frequency of AEs and on the number of laboratory values that fall outside of pre-determined ranges. AEs were described by event, duration, seriousness, intensity, and relationship to the investigational medicinal product, actions taken, and clinical outcome and reported as tables of frequencies at Preferred Term (PT) and MedDRA System Organ Class.

Phase I:

The primary safety endpoint was the determination of posterior probability of dose limiting toxicity (DLT), defined as all adverse events (AEs) coded using Medical Dictionary for Regulatory Activities (MedDRA) ≥ Grade 3 according to the National Cancer Institute Common Terminology Criteria for AE (CTCAE) v5.0 that are definitely, probably or possibly related to the administration of the investigational medical product in combination with radiotherapy.

The secondary safety endpoint was regimen-limiting toxicity (RLT), defined as any toxicity that meets the criteria of a DLT, but is observed after the end of the combination therapy in phase I or during phase IIa of the trial for patients recruited for phase I.

The secondary efficacy endpoint was progression-free survival at six months (PFS-6) according to response assessment in neurooncology (RANO) criteria as binary endpoint. See also the primary efficacy endpoint for phase IIa for more information.

Phase IIa:

The primary efficacy endpoint was the progression-free survival at six months (PFS-6) according to RANO criteria as binary endpoint. Response was defined as the proportion of patients without progression at six months after study entry. Basis for the baseline assessment of the disease progression was initial MRI ≤ 2 weeks before start of therapy (for radiotherapy planning).

Secondary efficacy endpoints were PFS and OS. PFS defined as time from study entry (day of attribution=baseline) until the day of first documentation of clinical or radiographic tumor progression or death of any cause (whichever occurs first). Patients without an event relevant for PFS (progression or death) at the time of analysis were censored at the last disease assessment showing no progression or at baseline if the patient has no post-baseline disease assessments. OS was defined as the time from study entry (day of attribution) until death due to any cause. Patients still alive or lost to follow-up at the time of the analysis were censored at the last date they were known to be alive.

Secondary safety endpoint was RLT, defined as any toxicity that meets the criteria of a DLT, but is observed after the end of the combination therapy in phase I (for patients recruited at the final dose of phase I) or during phase IIa of the trial.

Plants

Seed stocks	*Report on the source of all seed stocks or other plant material used. If applicable, state the seed stock centre and catalogue number. If plant specimens were collected from the field, describe the collection location, date and sampling procedures.*
Novel plant genotypes	*Describe the methods by which all novel plant genotypes were produced. This includes those generated by transgenic approaches, gene editing, chemical/radiation-based mutagenesis and hybridization. For transgenic lines, describe the transformation method, the number of independent lines analyzed and the generation upon which experiments were performed. For gene-edited lines, describe the editor used, the endogenous sequence targeted for editing, the targeting guide RNA sequence (if applicable) and how the editor was applied.*
Authentication	*Describe any authentication procedures for each seed stock used or novel genotype generated. Describe any experiments used to assess the effect of a mutation and, where applicable, how potential secondary effects (e.g. second site T-DNA insertions, mosiacism, off-target gene editing) were examined.*

Magnetic resonance imaging

Experimental design

Design type	not applicable, clinical study with structural MRI
Design specifications	not applicable, clinical study with structural MRI
Behavioral performance measures	not applicable, clinical study with structural MRI

Acquisition

Imaging type(s)	Clinical protocol including structural sequences, diffusion weighted MRI and perfusion-weighted MRI (DSC)
Field strength	3 Ts
Sequence & imaging parameters	FLAIR:<br>0.9×0.9×5.0 mm<br>TR 8500,0 ms<br>TE 136 ms<br>FoV 230 mm<br>FoV Phase 75,0 %<br>MPRAGE:<br>0.8×0.8×1.0 mm<br>TR 1750,0 ms<br>TE 3,65 ms<br>FoV 256 mm<br>FoV Phase 96,9 %<br>DWI:<br>1.1×1.1×5.0 mm<br>TR 3700 ms<br>TE 1 63 ms<br>TE 2 101 ms<br>FoV 220 mm<br>FoV Phase 100,0 %<br>SWI:<br>0.7×0.7×2.5 mm<br>TR 27,0 ms<br>TE 19,70 ms<br>FoV 230 mm<br>FoV Phase 75,0 %<br>T2ax<br>0.6×0.6×5.0 mm<br>TR 5180,0 ms<br>TE 90 ms<br>FoV 230 mm<br>FoV Phase 84,4 %<br>PWI:<br>1.9×1.9×5.0 mm<br>TR 2220 ms<br>TE 37,0 ms<br>FoV 240 mm<br>FoV Phase 100,0 %
Area of acquisition	Whole brain imaging

Diffusion MRI ☒ Used ☐ Not used

Parameters:

DWI:
b=0, 500, 1000
TA: 2:18
PM: ISO
Voxelgröße: 1.1×1.1×5.0 mm
PAT: 2
Schichtdicke 5,0 mm
TR 3700 ms
TE 1 63 ms
TE 2 101 ms
Verknüpfungen 1
FoV Auslese 220 mm
FoV Phase 100,0 %

Preprocessing

Preprocessing software	not applicable, clinical study with structural MRI
Normalization	not applicable, clinical study with structural MRI
Normalization template	not applicable, clinical study with structural MRI
Noise and artifact removal	not applicable, clinical study with structural MRI
Volume censoring	not applicable, clinical study with structural MRI

Statistical modeling & inference

Model type and settings	not applicable, clinical study with structural MRI
Effect(s) tested	not applicable, clinical study with structural MRI

Specify type of analysis: ☒ Whole brain ☐ ROI-based ☐ Both

Statistic type for inference (See Eklund et al. 2016)	not applicable, clinical study with structural MRI
Correction	not applicable, clinical study with structural MRI

Models & analysis

n/a	Involved in the study
☒	☐ Functional and/or effective connectivity
☒	☐ Graph analysis
☒	☐ Multivariate modeling or predictive analysis
